# Comprehensive biomarker profiles and chemometric filtering of urinary metabolomics for effective discrimination of prostate carcinoma from benign hyperplasia

**DOI:** 10.1038/s41598-022-08435-2

**Published:** 2022-03-14

**Authors:** Eleonora Amante, Andrea Cerrato, Eugenio Alladio, Anna Laura Capriotti, Chiara Cavaliere, Federico Marini, Carmela Maria Montone, Susy Piovesana, Aldo Laganà, Marco Vincenti

**Affiliations:** 1 Department of Chemistry, University of Turin, Via P. Giuria 7, 10125 Turin, Italy; 2 Department of Chemistry, Università di Roma “La Sapienza”, Sapienza University of Rome, Piazzale Aldo Moro 5, 00185 Rome, Italy; 3 Centro Regionale Antidoping e di Tossicologia “A. Bertinaria”, Orbassano, Turin, Italy

**Keywords:** Biomarkers, Oncology, Chemistry, Metabolomics

## Abstract

Prostate cancer (PCa) is the most commonly diagnosed cancer in male individuals, principally affecting men over 50 years old, and is the leading cause of cancer-related deaths. Actually, the measurement of prostate-specific antigen level in blood is affected by limited sensitivity and specificity and cannot discriminate PCa from benign prostatic hyperplasia patients (BPH). In the present paper, 20 urine samples from BPH patients and 20 from PCa patients were investigated to develop a metabolomics strategy useful to distinguish malignancy from benign hyperplasia. A UHPLC-HRMS untargeted approach was carried out to generate two large sets of candidate biomarkers. After mass spectrometric analysis, an innovative chemometric data treatment was employed involving PLS-DA classification with repeated double cross-validation and permutation test to provide a rigorously validated PLS-DA model. Simultaneously, this chemometric approach filtered out the most effective biomarkers and optimized their relative weights to yield the highest classification efficiency. An unprecedented portfolio of prostate carcinoma biomarkers was tentatively identified including 22 and 47 alleged candidates from positive and negative ion electrospray (ESI+ and ESI−) datasets. The PLS-DA model based on the 22 ESI+ biomarkers provided a sensitivity of 95 ± 1% and a specificity of 83 ± 3%, while that from the 47 ESI− biomarkers yielded an 88 ± 3% sensitivity and a 91 ± 2% specificity. Many alleged biomarkers were annotated, belonging to the classes of carnitine and glutamine metabolites, C21 steroids, amino acids, acetylcholine, carboxyethyl-hydroxychroman, and dihydro(iso)ferulic acid.

## Introduction

Molecular biomarkers can be classified as preventive, diagnostic, and prognostic. In particular, a diagnostic biomarker may have several potential targets, among which the detection of asymptomatic and/or early-stage cancers^1,2^ and the differentiation between benign and malignant disease^3–5^.

Studies performed on tumorous prostate cells revealed that they show a distinct metabolic profile, typically characterized by altered production of prostate-specific antigen (PSA), citrate, and polyamines^6^. Current prostate carcinoma (PCa) screening mainly relies on determining the PSA serum levels and digital rectal examination (DRE). Based on the results of these screening tests, trans-rectal ultrasound (TRUS)-guided prostate biopsy is commonly performed to confirm the diagnosis^7^. Unfortunately, increased PSA values are scarcely specific for PCa^1,8–10^, contributing to over-diagnosis and unnecessary biopsies^3,11–14^. These drawbacks support the need for better-performing biomarkers and inherent ongoing research.

A critical line of research for improving PSA diagnostic proficiency focused on its derived biomarkers, including PSA density, PSA velocity, and the ratio of free to total PSA^11^. The urinary dosage of the prostate cancer antigen 3 (PCA3) was overexpressed in more than 95% of the PCa. Higher scores apparently correlate with tumor aggressiveness, sustaining this biomarker's prognostic significance^15^. PCA3, alone or in combination with PSA^7^, is currently used and generally accepted by most urological societies^13,14,16^.

Metabolomics can be defined as the comprehensive and quantitative analysis of all the biological system metabolites under study^17^. Changes in metabolite concentration in biological fluids are frequently symptomatic of alterations in the physiological status of individuals^7^, making them valuable markers of pathological conditions^18,19^.

Among PCa metabolomics studies, a correlation was found between low spermine levels and citrate in prostate tissue and tumor aggressiveness^2,20^. Reduced levels of citrate, spermine, and myoinositol were found in prostatic secretion and seminal fluid from PCa patients^21^. Serum citrate, in combination with other metabolites (sarcosine, alanine, glycine, or polyamines), was proposed to differentiate PCa from benign prostatic hyperplasia (BPH)^22^. Another widely studied PCa potential biomarker is sarcosine, detected in prostate tissue, serum, and urine^7,21–28^. Its augmented concentration in urine^21,27^ and serum^26^ of PCa patients were further increased in case of metastatic tumors. However, a recent study questioned the use of sarcosine as PCa biomarker since reduced levels of sarcosine were found in PCa patients, and no correlation with tumor grade was observed^28^.

Altered levels of free amino acids in urine and serum samples were detected in PCa patients, with particularly relevant alterations observed for ethanolamine, arginine, and branched-chain amino acids^9^. Decreased urinary glycine levels, threonine, and alanine were noticed in a different study^8^. A multiplatform untargeted metabolomics study revealed the possible role of amino acids, urea, purine, and tricarboxylic acids metabolisms in prostate carcinoma pathogenesis^11^. A recent metabolomics study from our laboratories tentatively identified various amino acid and carnitine derivatives as potential PCa biomarkers^29^. The correlation between altered steroidal biosynthesis and PCa is also well established^30–35^. Previous studies were carried out in our laboratories using partial least square discriminant analysis (PLS-DA) to differentiate PCa from BPH based on an endogenous steroids panel quantified in urine^34^. Lastly, a recent metabolomics study based on serum samples collected from PCa and BPH patients and healthy controls highlighted that lipids and lipid-related metabolites may play a crucial role in the recognition of prostate malignancies^36^.

The present study compares the urinary metabolomics of a population of PCa patients with an analogous population of individuals afflicted by benign prostatic hyperplasia (BPH). This comparison is based on an untargeted UHPLC-HRMS (Orbitrap®) approach followed by in-depth statistical data analysis involving meticulous variable selection, PLS-DA classification modelling^37^, and repeated double cross-validation (r-dCV) of the classification model^38^. Such a statistically rigorous methodology allowed us to identify tens of promising biomarkers of different classes for PCa differential diagnostics and uncover some potential biochemical mechanisms underlying PCa metabolomics.

A few examples in the scientific literature investigate PCa metabolomics with an untargeted approach and different model computation and validation strategies. They include a study by Andras et al*.*^14^ in which a PLS-DA model was calculated after dividing the samples in a training and test set, an investigation by Zhang et al*.*^39^ that used orthogonal PLS-DA, and a study by Xu et al*.*^36^ in which a PLS-DA model was developed on 18 variables selected by variable importance in projection (VIP) and permutation tests. Similarly, Dereziński et al*.*^9^ and Kumar et al.^22^ used a test set to validate discriminant function analysis (DFA) models. Tanzeela et al.^40^ used the r-dCV to validate a random forest—linear discriminant analysis model to assess the diagnostic potential of urinary volatile organic compounds (VOCs). Lastly, a work published in 2019 by MacKinnon et al*.* applied the dCV on a large set of urine samples, combined with different variables selection strategies, including the variable importance in projection (VIP), the regression coefficients of the PLS model, and the competitive adaptive reweighted sampling (CARS)^41^.

## Results

The 22 (ESI+) plus 47 (ESI–) candidate biomarkers arising from the original UPLC-HRMS data's statistical analysis are reported in Tables 1 and 2, respectively.

**Table 1:** List of ESI + metabolites, together with their MW, chromatographic retention time, Kegg and HMDB IDs and NMDB classification, when available.

ID	Compound	Molecular weight	RT	HMDB ID	KEGG ID	Classification
1	Pyroglutamic acid	129.0427	7.48	HMDB0000267	C02237	Alpha aminoacids and derivatives
2	Methoxy benzaldehyde	136.0527	8.76	HMDB0029686	C10761	Benzoyl derivative
3	Acetylcholine	145.1104	0.82	HMDB0000895	C01996	Organonitrogen compound
4	Guanine	151.0496	1.02	HMDB0000132	C00242	Imidazopyrimidines
5	N-acetyl threonine	161.0690	0.88	HMDB0062557	N.A	Threonine metabolite
6	(N1)-Acetylspermidine	188.1764	0.64	HMDB0001276	C00612	Polyamines derivative
7	Amino hydroxydecanoic acid	203.1524	7.39	N.A	N.A	
8	4-methoxy-2-(3-methylbut-2-en-1-yl)benzene-1,3-diol	208.1102	13.62	N.A	N.A	
9	Dihydroxyl Indole O-Sulfate	229.0047	2.64	N.A	N.A	
10	Androstenone/etiocholanolone	272.2141	14.01	HMDB0000031 /HMDB0000490	C00523/C04373	C21 steroids
11	N-(indol-3-acetyl) glutamine	303.1220	8.24	HMDB0013240	N.A	Aminoacids, peptides, and analogues
12	Methoxyphenylacetyl carnitine	309.1578	8.01	N.A	N.A	Carnitine cycle
13	Carnitine azelaic acid	331.1996	9.27	N.A	N.A	Carnitine cycle
14	Dihydrocortisol	364.2250	11.97	HMDB0003259	C05471	Hydroxysteroids
15	Xanthurenate-8-O-beta-d-Glucoside	367.0905	7.05	HMDB0013118	N.A	Tryptophan derivative
16	Dihydro(iso)ferulic acid glucuronide	372.1066	8.33	HMDB0041723	N.A	Organooxygen compounds
17	Dihydro(iso)ferulic acid glucuronide	372.1067	8.75	HMDB0041723	N.A	Organooxygen compounds
18	Carnitine derivative	399.2621	11.44	N.A	N.A	Carnitine cycle
19	Dodecanedioyl glucuronic acid	406.1838	11.22	N.A	N.A	
20	5-Alpha-Dihydrotestosterone glucuronide	466.2566	14.01	HMDB0006203	N.A	C21 steroids
21	Tridecenoyl carnitine glucuronide	531.3056	13.88	N.A	N.A	Carnitine cycle
22	Urobilin	590.3110	12.03	HMDB0004160	C05794	Bilirubins

**Table 2:** List of ESI– metabolites, together with their molecular weights, chromatographic retention times, Kegg and HMDB IDs, and HMDB classification, when available.

ID	Compound	Molecular Weight	RT	HMDB ID	KEGG ID	Classification
1	Malic acid	134.0217	0.77	HMDB0000156	C00149	Hydroxyacid
2	Imidazolelactic acid	156.0537	0.77	HMDB0002320	C05132	Imidazoles
3	Hexanoylglycine	173.1055	8.81	HMDB0000701	N.A	N-acyl-alpha aminoacid
4	Dihydroxy-5-methylthio-4-pentenoic acid (DMTPA)	178.0302	1.73	HMDB0240388	N.A	Fatty acids and conjugated
5	Sulfooxybutanoic acid	184.0044	0.88	HMDB0130137	N.A	Fatty acids
6	Heptanoylglycine	187.1212	10.33	HMDB0013010	N.A	N-acyl-alpha aminoacid
7	N-lactoylvaline	189.1004	7.53	HMDB0062181	N.A	N-acyl-alpha aminoacid
8	Phenylacetylglycine	193.0743	7.43	HMDB0000821	C05598	N-acyl-alpha aminoacid
9	Ethylphenyl sulfate	202.0304	9.18	HMDB0062721	N.A	Arylsulfates
10	3-Hydroxy-3-(4-hydroxy-3-methoxyphenyl) propanoic acid	212.069	7.24	HMDB0133486	N.A	Phenylpropanoic acids
11	[2-Hydroxy-5-(prop-2-en-1-yl)phenyl]oxidanesulfonic acid	230.0253	9.31	HMDB0135258	N.A	Arylsulfates
12	5-Aminoimidazole-4-carboxamide glutaric acid	240.0865	3.12	N.A	N.A	Imidazole derivative
13	Indolylacryloylglycine	244.0852	10.33	HMDB0006005	N.A	N-acyl-alpha aminoacid
14	2-[4-hydroxy-3-(sulfooxy) phenyl]acetic acid	247.9995	2.34	HMDB0125151	N.A	Arylsulfates
15	Benzoyl glutamic acid	251.0799	8.02	N.A	N.A	Glutamic acid and derivatives
16	Propyl hydroxyhippuric acid	253.0956	8.18	N.A	N.A	N-acyl-alpha aminoacids and derivatives
17	5-(Hydroxyphenyl)-gamma-valerolactone-O-sulphate	272.036	7.96	HMDB0059993	N.A	Arylsulfates
18	Hydroxybutyric acid glucuronide	280.0801	1.32	N.A	N.A	
19	Methylguanosine	297.1082	4.79	HMDB0001563	C04545	Purine nucleosides
20	4-Methylcatechol O-glucuronide	300.0854	8.21	HMDB0240460	N.A	
21	Octenedioyl glutamine	300.133	7.15	N.A	N.A	Glutamic acid and derivatives
22	N-(indol-3-acetyl) glutamine	303.1226	8.29	HMDB0013240	N.A	Glutamic acid and derivatives
23	Succinyl tryptophan	304.1068	9.52	N.A	N.A	Tryptophan derivative (aminoacid)
24	2-Methoxy-4-vinylphenol glucuronide	326.1010	8.51	N.A	N.A	
25	Pyr-Xle-Ser	329.1596	7.40	N.A	N.A	Peptides
26	Hydroxy methoxy indole glucuronide	339.0964	8.87	HMDB0010363	C03033	Carbohydrates and carbohydrate conjugates
27	Suberoyl glucuronic acid	350.1223	8.64	N.A	N.A	
28	Alpha-CEHC sulfate	358.1096	11.96	N.A	N.A	Vitamin E metabolite
29	Feruloyl-quinic acid	368.1118	8.31	HMDB0030669	C02572	Quinic acids and derivatives
30	(epi)Catechin sulfate	370.0368	8.41	HMDB0012467	N.A	Sulfated flavonoids
31	Dihydro(iso)ferulic acid glucuronide	372.1066	8.38	HMDB0041723	N.A	Phenolic glycosides
32	Dihydro(iso)ferulic acid glucuronide	372.1067	8.80	HMDB0041723	N.A	Phenolic glycosides
33	Dimethylene suberic acid glucoronide	374.1222	9.37	N.A	N.A	Medium chain fatty acids
34	Dimethylene suberic acid glucoronide	374.1224	9.24	N.A	N.A	Medium chain fatty acids
35	Decenedioyl glucuronic acid	376.1379	10.40	N.A	N.A	
36	Methylcathecol glucuronide sulfate	380.0424	5.72	N.A	N.A	
37	Methyl(epi)catechin sulfate	384.0525	8.89	N.A	N.A	
38	Hydroxyandrosterone sulfate isomer	386.1773	12.93	N.A	N.A	C21 steroids
39	Dodecanedioyl glucuronic acid	406.1847	11.28	N.A	N.A	
40	Androstenol glucuronide	450.2626	15.77	N.A	N.A	C21 steroids
41	Alpha-CEHC glucuronide	454.1848	11.94	HMDB0062445	N.A	Vitamin E metabolite
42	Uroerythrin (biotrypirrin A)	465.1910	13.84	HMDB0003323	N.A	Pyrroles
43	(epi)Catechin glucuronide	466.1121	8.82	HMDB0240435	N.A	Flavonoids
44	Trihydroxyoctadecenoic acid glucuronide	506.2734	12.94	N.A	N.A	
45	Trihydroxyoctadecenoic acid glucuronide	506.2734	13.35	N.A	N.A	
46	Trihydroxycholanoic acid glucuronide	584.3209	14.77	N.A	N.A	
47	(3a,5b,7a,12a)-24-[(Carboxymethyl)amino]-1,12-dihydroxy-24-oxocholan-3-yl-b-d-Glucopyranosiduronic acid	641.3428	12.61	HMDB0002472	N.A	Oximes

The numerical IDs in the first column correspond to those used in Fig. 4B.

**Figure 1:**
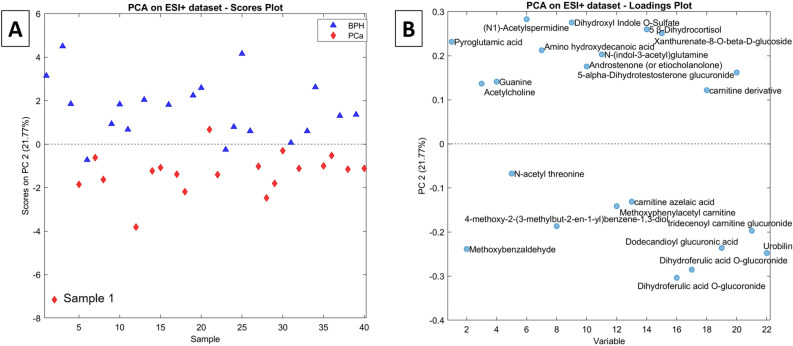
(**A**) scores plot and (**B**) loadings plot of the PCA model for the autoscaled ESI + dataset.

The PCA model performed on the ESI + final dataset (of dimensions 40 × 22) showed good separation between PCa and BPH patients classes in the second principal component (PC2 in Fig. 1A). In particular, positive PC2 values are recorded for 18 out of 20 BPH patients, while negative PC2 values are observed for 19 out of 20 patients affected by PCa. The corresponding loadings plot (Fig. 1B) shows a strong polarization of the variables along the same direction. Sample 1 apparently overexpresses the PC2 score, as is confirmed in the Q-residuals vs Hotelling’s T^2^ plot (Supplementary Fig. S5A), in which sample 1 shows a high T^2^ value (equal to 4.2) but < 1 Q-residual. A possible explanation for this overexpression may rely on the anomalously low urinary creatinine value (close to 5 mg dL^−1^). Since the contribution plot (Fig. S5B,C) did not reveal any specific inconsistency for sample 1, it was decided not to discard it from the dataset.

**Figure 2:**
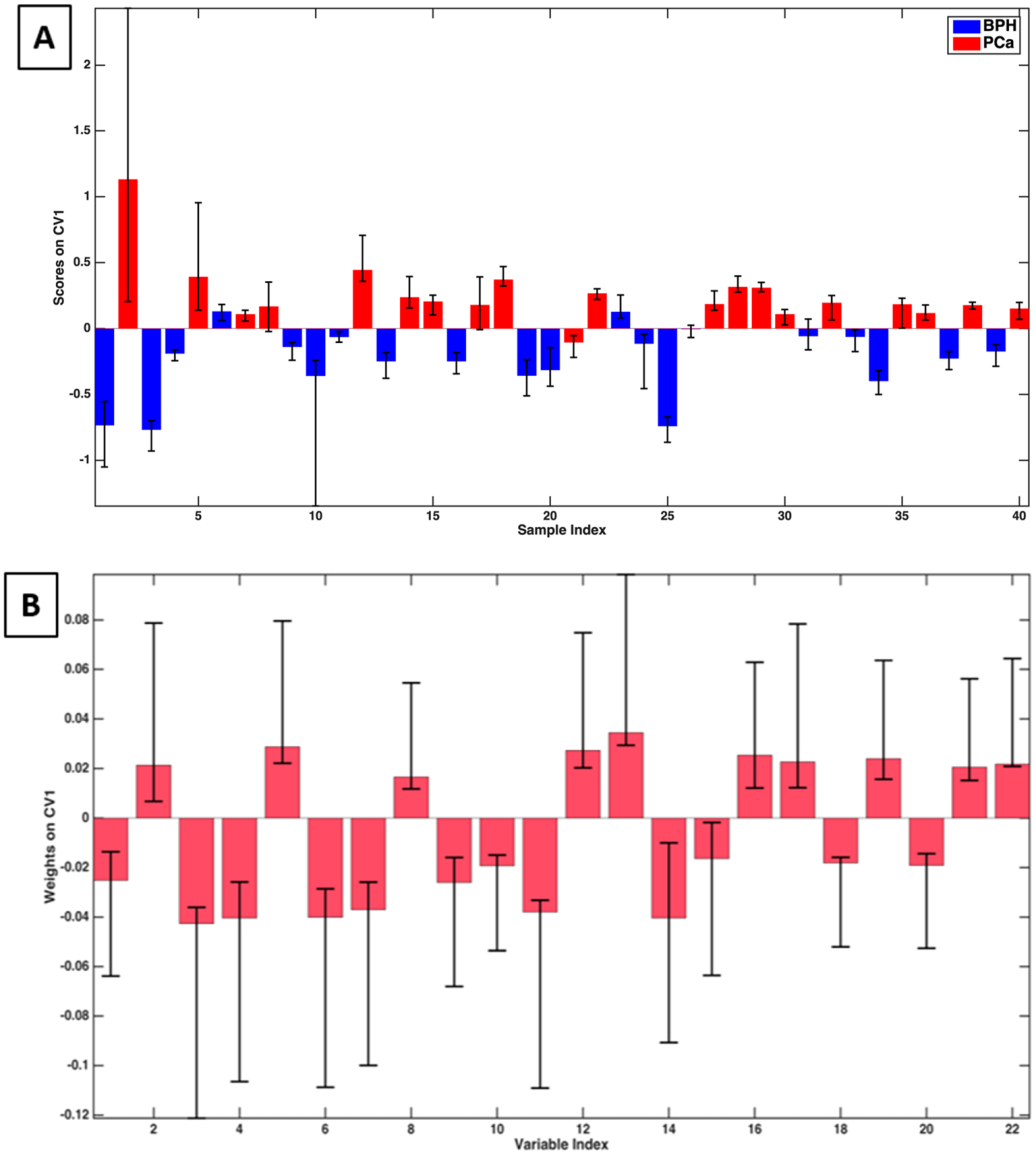
Graphical representation of the PLS-DA r-dCV model obtained for the ESI + dataset. (**A**) Scores of the samples along the first canonical variable. (**B**) Weights of the variables along the first canonical variable.

The PLS-DA r-dCV model provided a sensitivity of 95 ± 1% (corresponding to the correct prediction rate for PCa population) and a specificity of 83 ± 3% (corresponding to BPH correct prediction rate). The classification error rate is equal to 9 ± 1%. The accuracy of the model is 89 ± 2%. The sample scores along the canonical variable are reported in Fig. 2.

The bars represent the average values obtained during the r-dCV process for samples (A) and variables (B), while the interval ranges correspond to the confidence intervals (at 95% confidence, estimated non parametrically from the distributions obtained by r-dCV). For most of the samples, the scores maintain the same sign during the r-dCV process, confirming the stability of the model. For four samples (n. 8 (PCa), 17 (PCa), 26 (BPH), and 31 (BPH)), the confidence interval of the corresponding score crosses the zero-line, resulting in a more uncertain classification. However, it should be stressed that, in all of the cases, the largest part of the confidence interval falls within the correct side of the plot (negative scores for BPH and positive for PCa). On the other hand, there are three samples (nr. 6 (BPH), 21 (PCa), and 23 (BPH)) that are consistently mispredicted and mostly responsible for the observed classification error. Similarly, all variable weights along the canonical variable (Fig. 2B) keep the same sign across all the r-dCV procedures, providing further confirmation of the model robustness, even if they show a relatively high confidence interval. This means that during the entire cross-validation process all the variables consistently proved to be overexpressed for one of the two categories (PCa or BPH, corresponding to positive and negative sign, respectively—see Fig. 2A) no matter which samples were selected in each step of the reiterated validation.

**Figure 3:**
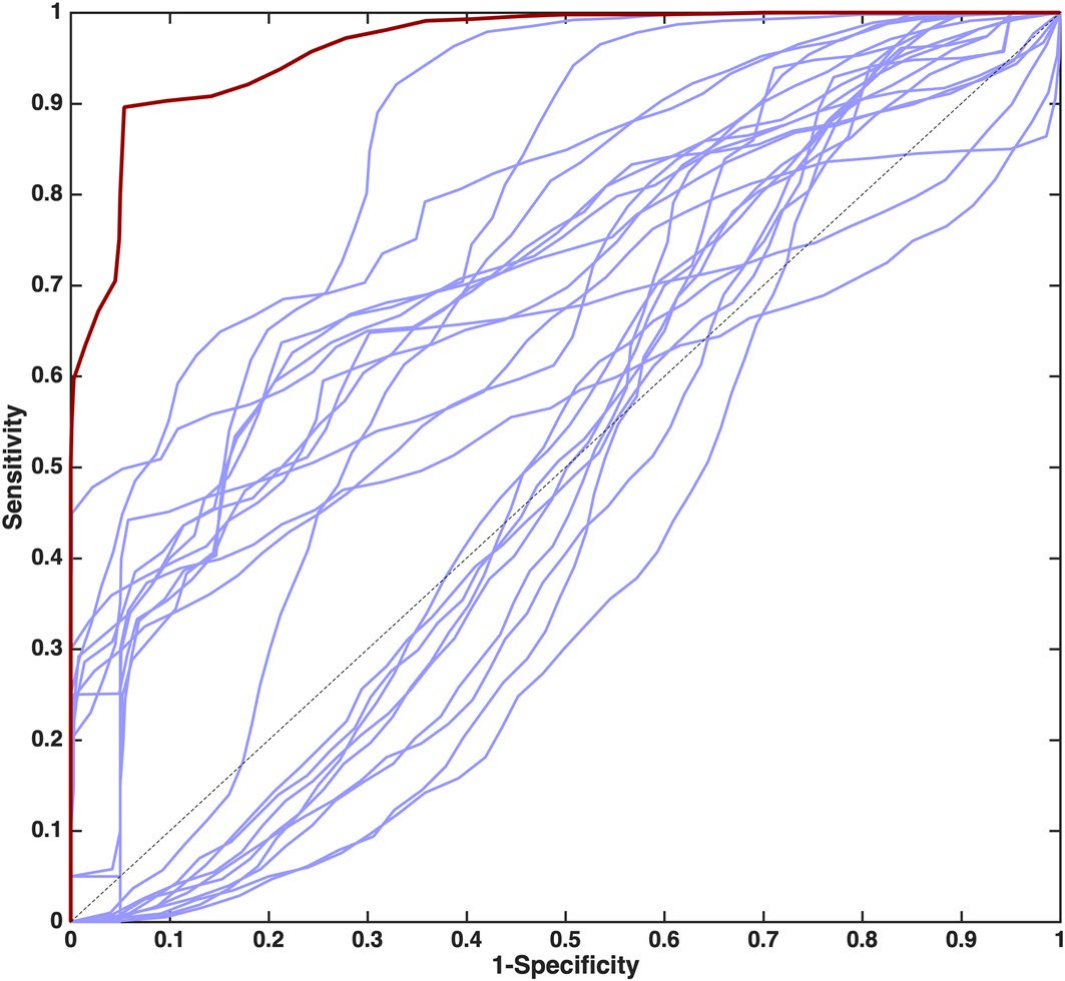
Receiver operating characteristic (ROC) curve for the PLS-DA model calculated on the ESI + data set (dark red line) and for the individual metabolites (blue lines). Each curve is the mean of the 50 curves obtained on the outer loop samples in r-dCV.

The diagnostic accuracy of the model can also be graphically visualized through a receiver operating characteristic (ROC) curve, i.e., a plot of the sensitivity *vs.* 1-specificity, and summarized by the value of the area under the curve (AUROC): the closer the value of the AUROC is to 1, the better the classification model. Taking advantage of the r-dCV procedure, analogously to what already discussed for the other figures of merit, it was possible to calculate, based on the outer loop samples, as many ROC curves as the number of dCV repetitions (50), so to estimate the mean and CI for the curves on external validation samples and, consequently of the AUROC values. The mean ROC curve for the PLS-DA model built on the ESI + variables is displayed in Fig. 3. The corresponding value of the AUROC is 0.963 ± 0.011.

The importance of building a multivariate model can be highlighted by comparing the performances of the PLS-DA model built on the ESI + variables with those of individual metabolites, which are summarized in Supplementary Table S1. Indeed, it can be observed how the best performing individual metabolite, i.e., dihydrocortisol, has a predictive accuracy of 76 ± 2, corresponding to an AUROC of 0.810 ± 0.016. This can also be graphically visualized in Fig. 3, where the mean ROC curves estimated on the outer loop samples in r-dCV for the individual metabolites are displayed.

**Figure 4:**
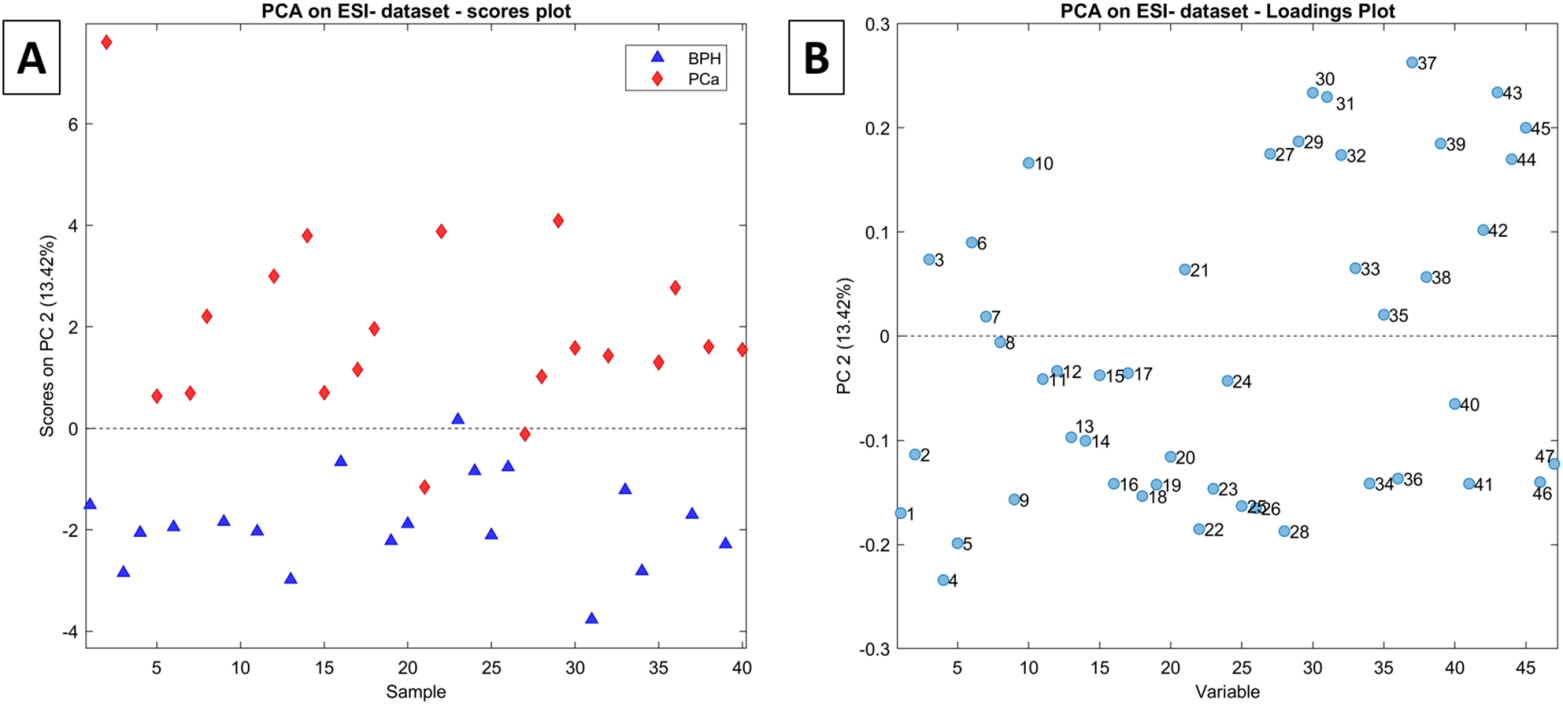
(**A**) scores plot and (**B**) loadings plot of the PCA model for the autoscaled ESI– dataset. The loadings labels are reported in Table 2 (ID column).

Analogously to what was observed for the ESI + dataset, the PCA model built on the ESI– dataset showed good separation between the two groups of patients (BPH and PCa) along the PC2, where positive values are recorded for the PCa samples (18 out of 20) and negative values for BPH samples (19 out of 20) (Fig. 4). The PCA loadings depicted in Fig. 4B (the variables corresponding to the numerical labels are reported in Table 2 as “ID” column) show a less pronounced polarization along PC2 than is observed in Fig. 1B.

**Figure 5:**
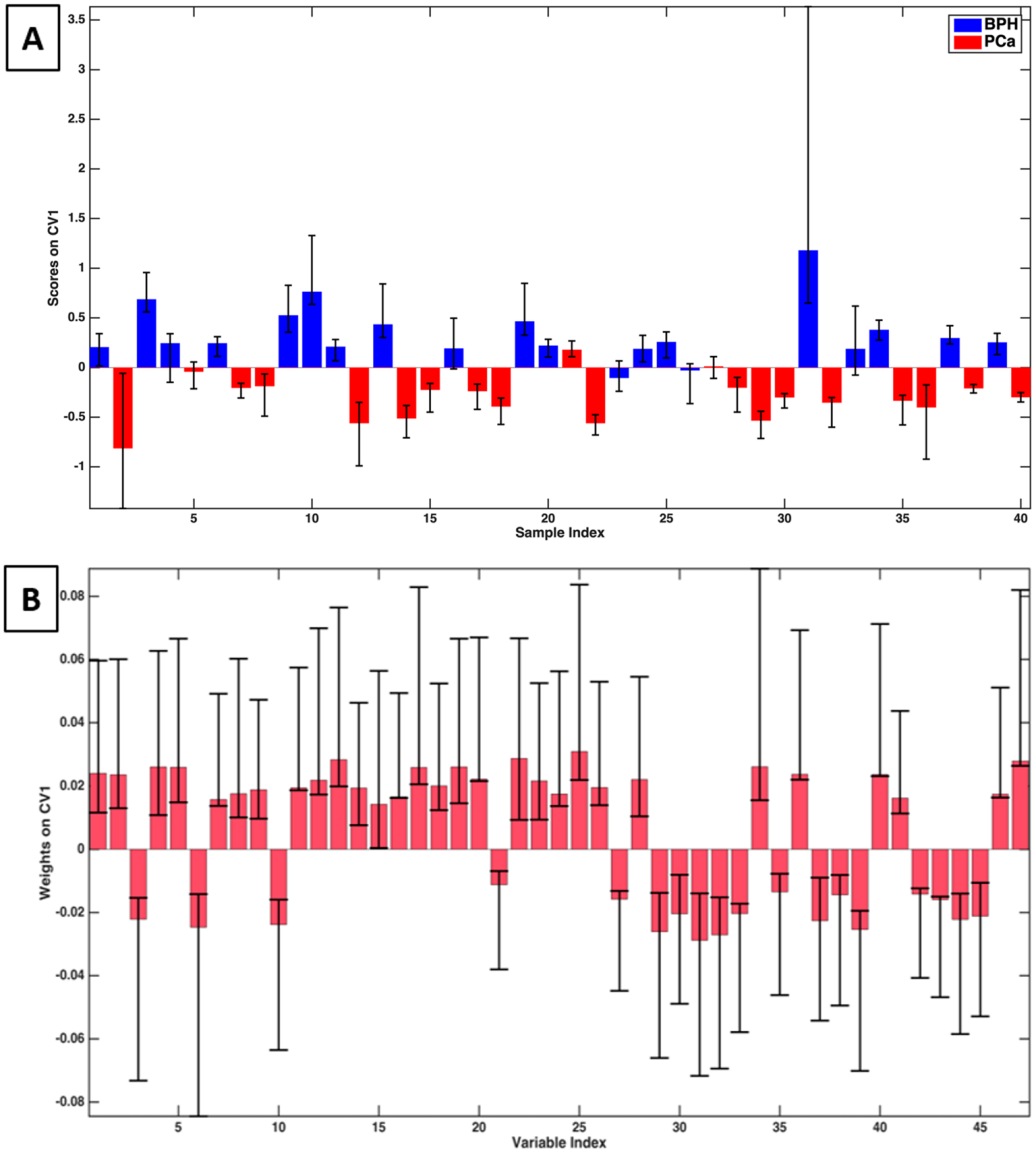
Graphical representation of the r-dCV model obtained for the ESI– dataset. (**A**) Scores of the samples along the first canonical variable. (**B**) Weights of the variables along the first canonical variable.

The r-dCV PLS-DA model developed on the 47 ESI– candidate biomarkers listed in Table 2 provided a sensitivity equal to 88 ± 3%, a specificity score of 91 ± 2%. The classification error rate was equal to 11 ± 2%. Finally, the accuracy of the model was 89 ± 2%. Figure 5 reports the scores and loadings values, together with their confidence bars, obtained during the r-dCV model computation. As far as the samples are concerned (Fig. 2A), for most of the individuals, the confidence interval of the scores falls consistently on the same side of the plot (negative for PCa and positive for BPH). The only exceptions are samples n. 4 (BPH), 5 (PCa), 16 (BPH), 23 (BPH), 26 (BPH 27 (PCa), and 33 (PCa), which show an uncertain classification. This may be due to them having a borderline character, for example, a PCa at an early stage of development or a BPH evolving toward a pre-cancerous state. These conditions may result in an incomplete expression of either class characteristic biomarkers. However, as also discussed in the case of the ESI+ data set, for most of these samples, the largest part of the confidence interval falls within the “correct” side of the plot. On the other hand, sample 21 (PCa) is consistently characterized by positive scores and therefore consistently mispredicted as BPH. This represents the most relevant contribution to the classification error, together with sample 23 (BPH), which is assigned to the wrong category the large majority of the time, and sample 26 (BPH), which was mispredicted in about half of the r-dCV repetitions.

As far as the variables are concerned, all the weights along the canonical variable (Fig. 5B) keep the same sign across all the r-dCV procedures, providing further confirmation of the model robustness, even if they show a relatively high confidence interval.

**Figure 6:**
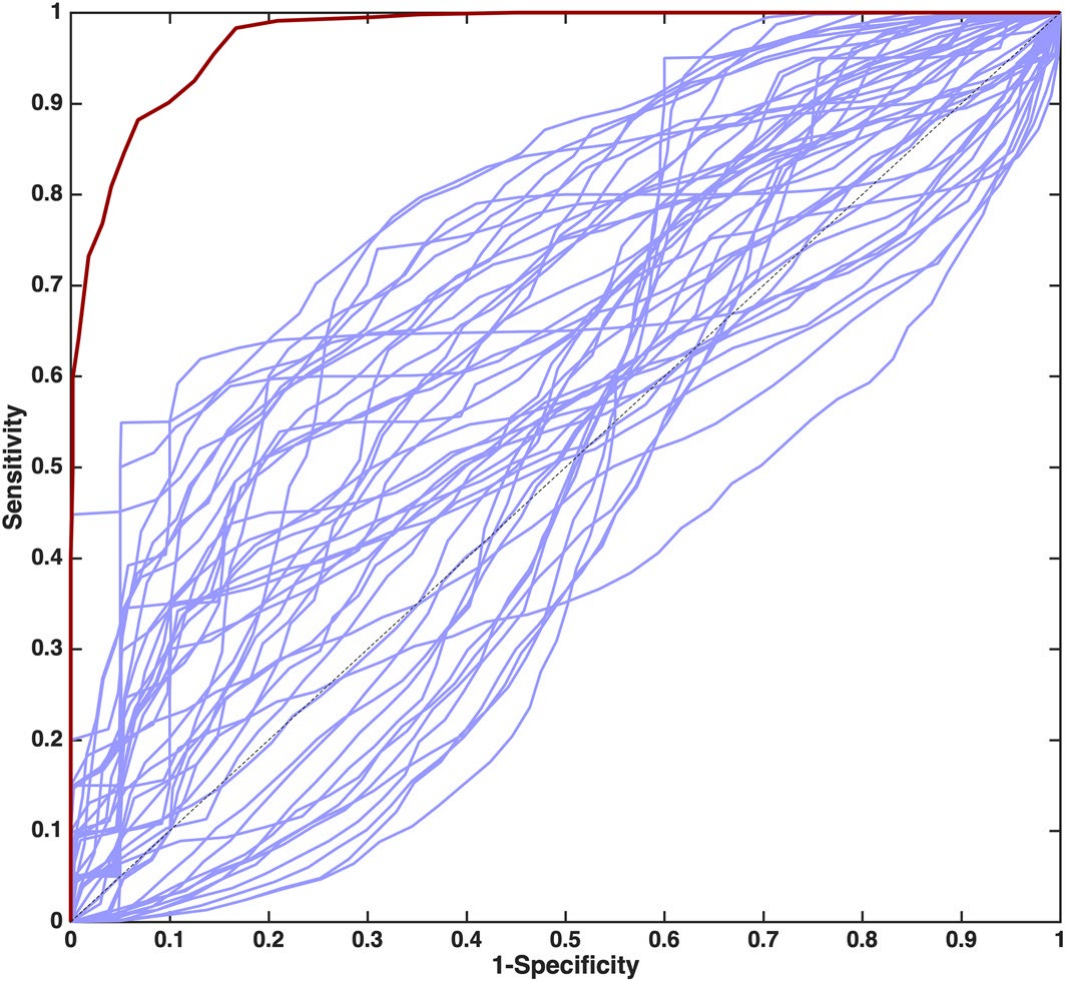
Receiver operating characteristic (ROC) curve for the PLS-DA model calculated on the ESI− data set (dark red line) and for the individual metabolites (blue lines). Each curve is the mean of the 50 curves obtained on the outer loop samples in r-dCV.

Also in this case, the diagnostic accuracy of the model can be graphically visualized through the mean receiver operating characteristic (ROC) curve, estimated on the outer loop validation samples in r-dCV, which is displayed in Fig. 6, together with those corresponding to the individual metabolites.

The value of the AUROC for the multivariate model built on ESI- variables is 0.975 ± 0.009, which is significantly higher than the highest value for the individual metabolites (0.802 ± 0.056, for decenedioyl glucuronic acid), thus confirming once again that building a multivariate model can significantly improve the diagnostic accuracy, as also confirmed by the inspection of the other figures of merits summarizing the predictive performances of individual variables, reported in Supplementary Table S2.

Furthermore, t-tests were conducted on the individual metabolites both for the ESI+ and the ESI− data sets (Supplementary Table S3) and the distribution of the values of the predictors for the two classes graphically inspected by means of box plots (reported in Supplementary Material). The compounds showing significant p-values (i.e., showing a non-null difference between the average values PCa and control groups) are relatively consistent with those indicated by PLS-DA weight plots (Figs. 2B and 5B).

Lastly, a multi-block analysis was tested by merging the two (ESI+ and ESI−) reduced datasets, resulting in 90 ± 3% value of sensitivity and 87 ± 4% of specificity. The performance of the latter model is almost indistinguishable from that obtained from the ESI– dataset. This is also confirmed by the value of the area under the ROC curve (0.979 ± 0.008). This similarity may be due to the effect of the larger number of variables included in the ESI– dataset compared to ESI+ , which was only partly compensated by scaling each block to unitary Frobenius’ norm prior to model building.

## Discussion

### Chemometric classification models

The present study recruited 40 patients equally distributed between PCa and BPH patients with the unequivocal diagnosis. The initial ESI+ and ESI– untargeted UHPLC-HRMS analyses resulted in detecting 2611 and 1610 potentially discriminant metabolites, respectively. The availability of such an unbalanced number of samples vs. variables justifies the choice of PLS-DA as a classification method, as is commonplace in metabolomics studies involving unbalanced datasets. The main risk of using a PLS-DA approach on datasets with a limited number of samples is represented by the potential occurrence of overfitting, yielding over-optimistic models. Two combined procedures consisting of a repeated double cross-validation process^38^ and applying a permutation test were carried out to overcome the mentioned threat and produce reliable and robust models (Fig. S3). The repeated-double cross-validation process involves an iterative approach in which all the samples are in turn included either in the calibration or in the validation set. This is a recommended strategy when, as in the present case, it is impossible to divide the dataset into a training and test set due to the relatively low number of samples. In an iterative permutation test, the samples' labels are randomly redistributed, and a new classification model is calculated each time (Fig. S4)^42^. Whenever the latest models' performances obtained from the permuted class labels are significantly and systematically lower than those obtained with the original one, then the original model can be assumed as robust and reliable. Another step in our strategy was to operate a primary variables selection to exclude those not carrying important information for the discrimination of the two classes. As a result, even the unsupervised exploration of PCA data provided good class separation (Figs. 1A and 4A). The high classification rates offered by both PLS-DA models built on the positive and negative ions datasets (ESI+ and ESI–) confirmed the occurrence of structured information in the data relative to the differences between the metabolic profiles of the two classes considered (BPH and PCa). Concurrently, the low standard deviations of classification rates obtained with the r-dCV approach corroborate the models' robustness. The scores and loading results depicted in Figs. 2 and 5 show that the iterative r-dCV process, while producing significant shifts in their absolute values, does not alter their sign, demonstrating that the samples maintain their original classification and the variables maintain their positive or negative correlation with each class. Quite obviously, all considerations about the under- or over-expression of biomarkers in PCa patients are identical in PCA and PLS-DA models. Further confirmation of the model's robustness arises from the permutation tests, which always resulted in classification rates close to 50%, i.e. extremely far from the two model rates. In practice, no random sample assignment is even vaguely able to simulate a correct classification, as expected for unbiased models. All these chemometric tests support the deduction that the selected metabolic biomarkers play a role in the correct classification of PCa and BPH patients. However, it is evident that the limited number of samples in each class (20) used to build the models does not allow us to draw undeniable conclusions about their actual effectiveness and their relative importance nor definitive non-error rates in classification PCa vs. BPH. Much larger patients’ populations will be analyzed to rank the detected biomarkers and interpret their role in the PCa etiology and/or metabolic effect, whose preliminary classification is reported in the subsequent chapters of the Discussion. Nevertheless, the present study anticipates a meaningful recognition of beneficial PCa biomarkers families and provides a practical chemometric approach for interpreting UHPLC-HRMS metabolomics data. On the other hand, many alleged biomarkers preliminarily identified in the present study are significant because they may cast light on the general underlying altered biochemical mechanisms comprehensively expressed in the biomarkers diversity.

### Biomarkers classification

The chemometric classification model discussed in the preceding chapter is founded on the multivariate interpretation of the statistically significant concentration differences of the candidate biomarkers reported in Tables 1 and 2 between the two populations of PCa and BPH patients. These candidate biomarkers can either be overexpressed or underexpressed in the urine of the two populations, resulting in positive/negative loadings of the upper/lower classes represented in Figs. 2 and 5. It was out of the scope of the present study to investigate in detail the underlying biochemical mechanisms that may justify these differences. Simultaneously, some considerations about the potential role of some groups of these substances in the carcinogenic processes can be made concerning the existing literature, as is reported in the subsequent paragraphs.

Four alleged biomarkers are involved in the *carnitine metabolic cycle* (i.e., tridecenoyl carnitine glucuronide, carnitine azelaic acid, methoxyphenylacetyl carnitine, and one carnitine derivative not univocally identified (Table 2). In particular, the urinary profiles of PCa patients appeared to be characterized by high levels of methoxyphenylacetyl carnitine, carnitine azelaic acid, and tridecenoyl carnitine glucuronide. In contrast, the unidentified carnitine derivative was underexpressed. The correlation between the carnitine cycle and cancer incidence was explained by Melone et al.^43^, which suggested that the carnitine derivatives are implicated in the bi-directional transportation of acyl moieties from the cytosol to mitochondria, so regulating the toggle between glucose and fatty acid metabolisms. Carnitine derivatives were singled out as valuable biomarkers for different tumors, including breast^44^ and renal^45^ cancers. Our previous metabolomics study on PCa and BPH samples also evidenced the role of some unidentified carnitine derivatives in the patients’ class discrimination^29^. N1-acetylspermidine is a metabolite of spermidine, one of the three principal polyamines involved in the human metabolism and its precursor putrescine and its metabolite spermine^46^. Decreased polyamines' values in the urine of PCa patients compared to BPH-affected subjects were observed in a study from Tsoi^47^. Similarly, in our study, the reduced presence of the acetylated form of spermidine was observed in PCa patients.

*C21 steroids* are known to be involved in prostate cancer cells' growth and proliferation. Several studies were carried out on this correlation, including our own^30–32,35^. In the present work, reduced levels of dihydrocortisol, 5α-DHT-glucuronide, androstenone (or etiocholanolone), androstanol glucuronide, and hydroxyandrosterone sulfate were found in the PCa population compared to BPH patients. The complex equilibria occurring between steroids production and excretion of their conjugated forms in PCa and BPH patients are expected to substantiate the present results.

The *glutamine metabolic pathway* is involved in several steps of cellular life and proliferation. A deficiency of glutamate, glutamine, and associated metabolites is observed in patients with cancer due to their malignant cells' depletion^48^. In the present ESI+ dataset, pyroglutamic acid and N-(indol-3-acetyl)glutamine urinary concentration appears to be reduced in PCa patients compared to BPH patients. The same trend was observed in the ESI– dataset for N-(indol-3-acetyl)glutamine and benzoyl glutamic acid, whereas the concentrations of octanedioyl glutamine were comparable for the two populations. The *acetylcholine* receptors play an essential role in developing a vast number of cancers, including bladder, gastric, lung, breast, ovarian cancers, melanoma, glioblastoma^49^. The muscarinic acetylcholine receptor M1, in particular, was found over the cell membrane and cytoplasm in prostate cancer cells. Furthermore, it appears to regulate cancer metastasis^49^. The *amino acids* play a vital role in cancer cell growth and reproduction^50^. Several research groups found a correlation between altered amino acids and PCa incidence levels, including our own^8,9,11,29^. Coherently with these findings, the present study confirms the importance of aminoacidic biomarkers in detecting PCa: twelve of the metabolites reported in Tables 1 and 2 belong to this class or are directly related to them. Two amino acids involved in the tryptophan cycle are of particular interest*,* namely succinyl tryptophan and xanthurenate-8-O-beta-d-glucoside, in agreement with a recent review underlining the role of tryptophan in cancer proliferation^51^). A protective effect against malignant tumors has been attributed to various endogenous metabolites, including vitamin E^52^. Under this evidence, we found an underexpression of the glucuronide and sulfate conjugated forms of *CEHC* (its most soluble metabolites) in PCa patients. Another metabolite with an alleged protective role against cancer is ferulic acid^53^. In our dataset, the glucuronide derivatives of the *dihydro(iso)ferulic acid* were detected in both ESI+ and ESI– ionization modes. Overexpression of these metabolites in the urines collected from PCa patients was recorded, possibly due to accelerated conjugation and excretion (i.e., accelerated metabolism) of ferulic acid, resulting in a lower bioavailability of the protective compound.

## Materials and methods

### Chemicals and reagents

Creatinine, taurine, putrescine, dopamine, guanosine, cystine, benzoic acid, formestane, dihydrotestosterone (DHT) glucuronide, taurocholic acid were purchased from Sigma Aldrich (Milan—Italy). Arginylphenylalanine (RF), serylhistidine (SH), and isoleucylprolylisoleucine (IPI) were provided by Thermo Fischer Scientific (Italy). Isotopically labeled caffeine, creatinine, phenylalanine, benzoic acid, estradiol, estradiol sulfate, and estradiol glucuronide were purchased from Sigma Aldrich. Ultrapure grade water and formic acid were from Fischer Scientific (Waltham, Massachusetts, USA), while ultrapure grade methanol (MeOH) was from Romil Pure Chemistry (Pozzuoli, Italy). The creatinine was determined with the photometric picrate technique, using an Architect C800 instrument and Abbott's kit (Italy). All standard stock solutions were prepared in methanol at 1 mg/mL and stored at – 20 °C until use. The external standard mix solution, containing creatinine, taurine, putrescine, dopamine, guanosine, cystine, benzoic acid, formestane, DHT glucuronide, taurocholic acid, RF, SH, and IPI, was prepared at the final concentration of 0.1–50 µg mL^−1^ in H_2_O/MeOH 80:20 (*v/v*) by appropriate dilution with ultrapure water and stored at − 20 °C until use. The internal standard mix solution, containing isotopically labeled caffeine, creatinine, phenylalanine, benzoic acid, estradiol, estradiol sulfate, and estradiol glucuronide, was prepared at the final concentration of 0.1–5 µg/mL in H_2_O/MeOH 80:20 (*v/v*) by appropriate dilution with ultrapure water and stored at − 20 °C until use.

### Patients recruitment and samples collection

The subjects involved in this study were recruited in the Department of Urology at the San Luigi Hospital of Orbassano (TO, Italy) after approval from the reference Ethical Committee of the hospital (protocol number 17942). All research was performed in accordance with the Declaration of Helsinki. Informed consent was signed by all patients enrolled in this study. Forty patients were enrolled, including 20 affected by PCa and 20 by benign prostatic hyperplasia (BPH). PCa was diagnosed employing untargeted systematic transrectal ultrasound-guided prostate biopsy (TRUS-GBx, 18–24 cores) and/or repeated multiparametric magnetic resonance imaging (mp-MRI) target biopsy (4–6 cores for single target lesion^54^). Urine samples were collected from the PCa patients before they started any specific treatment/therapy, either pharmacological, surgical, and/or radiologic.

Body mass index (BMI), previous medical therapy, PSA, and prostate volume were recorded for all patients. Biopsy Gleason Score (GS) was also reported for PCa patients. Table 3 reports the patients’ biometrics and the principal clinical data. Patients affected by diabetes, other carcinomas, and metabolic diseases were not included in the study. Furthermore, Student's t-tests was performed on the enrolled patients to investigate the occurrence of significant differences between the groups. As expected, only PSA and prostate volume variables (i.e., the peculiar features of PCa and BPH groups) provided a statistically significant p-value lower than 0.05 (95% level of significance).

**Table 3:** Characteristics and clinical data of the enrolled patients.

	PCa	BPH	t-test (p-value)
Age (mean ± std) years	66 ± 7	65 ± 6	0.48
Body weight (mean ± std) kg	82 ± 11	79 ± 11	0.51
Body height (mean ± std) cm	176 ± 6	172 ± 8	0.07
BMI (mean ± std) kg/m^2^	26.4 ± 3.1	27.0 ± 4.4	0.60
PSA (median–range) ng/mL	7.4 (3.0–22.0)	2.7 (0.3–19.8)	0.01*
Prostate volume (median–range) cm^3^	42 (20–98)	60 (30–200)	0.01*

Biopsy GS	Number of patients		
3 + 4	5	/	
4 + 3	7	/	
4 + 4	4	/	
4 + 5	2	/	
5 + 4	2	/	

Symbol (*) indicates the features showing a significant p-value (lower than 0.05) for the performed Student's t-tests.

The urine samples were collected during the outpatient activities, between 8 a.m. and 10 a.m., using test tubes of 50 mL; immediately after collection, five aliquots of 100 μL were separated and stored at − 80 °C temperature. Soon afterward, they were sent in dry ice by express courier to the Department of Chemistry of the University La Sapienza of Rome (Italy) for instrumental analysis. The remaining volume was stored at 4 °C until the creatinine test was performed (using the photometric picrate determination).

### Samples preparation

Before the analysis, the urine samples were thawed at room temperature and centrifuged for 10 min at 1000×*g*. The quality control (QC) samples were obtained by pooling 20 μL of each of the 40 samples included in the study. For each sample (samples, controls, and QCs), 25 μL of urine were diluted in 70 μL of ultrapure water and added 5 μL of the internal standard mixture (dilution 1:3, final solvent mixture: H_2_O/MeOH 99:1, *v/v*). External standard mixture samples were prepared by diluting 5 μL of the mixture in 95 μL of ultrapure water (final solvent mixture: H_2_O/MeOH 99:1, *v/v*), and the blank samples consisted of H_2_O/MeOH 99:1 (*v/v*) The UHPLC-HRMS analyses were performed after samples randomization.

### UHPLC-HRMS analysis

A Vanquish binary pump H (Thermo Fisher Scientific, Bremen, Germany), equipped with an autosampler and controlled temperature column compartment, was used for chromatographic separation on a Luna Omega Polar C18 (100 × 2.1 mm, 1.6 μm particle size, Phenomenex, Torrance, USA). The mobile phases were H_2_O/HCOOH (99.9:0.1, *v/v*; phase A) and MeOH/HCOOH (99.9:0.1, *v/v*; phase B) and were mixed with the following gradient: 1% phase B for 2 min; 1% phase B to 99% phase B in 15 min; 99% phase B for 5 min (washing step) and 1% phase B for 5 min (reconditioning step). The column was maintained at 40 °C with a constant flow of 400 μL min^−1^. The chromatographic system was coupled to a hybrid quadrupole-Orbitrap mass spectrometer Q Exactive (Thermo Fisher Scientific) with a heated ESI source, operating in both positive and negative ion modes under the following conditions: the capillary temperature was set at 220 °C and 180 °C for positive and negative polarity respectively, spray voltage at 3200 V (+) and 2800 V (−), auxiliary gas heater temperature at 280 °C (+) and 180 °C (−), sheath gas at 50 (arbitrary units), auxiliary gas at 25 (arbitrary units), sweep gas was 0 (arbitrary units), and S-Lens RF level was 50 (%).

Full scan acquisition mode was performed in the range m/z 70–1000 with a resolution of 70,000 (full width at half-height, FWHM, *m*/*z* 200). The automatic gain control (AGC) target value was 500,000 in full scan, with a maximum ion injection time set at 50 ms. The isolation window width was 2 m/*z*. For *identification-only* QCs, the top 5 data-dependent acquisition (DDA) mode was performed with the AGC target set at 100,000. Higher-energy collisional dissociation (HCD) was performed at 35% normalized collision energy with a resolution of 35,000 (FWHM @*m*/*z* 200). Dynamic exclusion was set to 3 s. The mass spectrometer was calibrated before analysis using a calibration solution provided by the manufacturer (external calibration).

Raw MS/MS data files were acquired by Xcalibur software (version 3.1, Thermo Fisher Scientific). The chromatographic worklist is schematized in Supporting Information Table S6. The column stability and performance were tested before and after each analytical section using blank samples and external standard solutions. A proper system conditioning preceded the blank sample injection for background subtraction, consisting of ten consecutive QCs sample injections. This procedure allowed to discard both the contaminants present in mobile phases and the HPLC–MS system and the compounds subjected to high carry-over effects (more than 10%), which may alter peak areas, possibly resulting in biased statistical analysis. After further system reconditioning with ten more QCs samples, randomized samples and controls were run in five groups, followed by a QC injection. In HRMS, the chromatogram is recorded in the digital format using each scan in full-scan mode as a point for each m/z analyzed. As the instrument's scan rate is fixed, tandem MS analysis, either in DDA or data-independent acquisition (DIA) mode, causes a drastic decrease in the number of points per chromatographic peak. Therefore, samples and controls were run in single-MS full-scan mode, to guarantee high-quality peak shapes for high- and low-abundance substances^55^. At the end of each sequence, three QC injections (*identification-only* QC) were run in top 5 DDA mode, consisting of one full-scan acquisition followed by 5 tandem MS scans to obtain MS/MS for subsequent feature identification. The external standard mixture was run at the start and the end of the acquisition sequence for a quick evaluation of the performance of the LC–MS methodology before and after data acquisition. Internal standard spiked in the samples were employed to rapidly check potential outliers or macroscopic damages during analysis, e.g., instrumental errors during sample injection or significant change in compound retention times, rather than used for sample normalization, which was later accomplished during data processing by QC-based normalization. Since untargeted MS data cannot be easily inspected, the rapid check with the internal standard mixture during data acquisition was needed for eventually re-running damaged samples before the end of the worklist or to stop and re-run the whole sequence if chromatographic or MS performance were unstable. Exemplary chromatograms of samples, controls, and QCs are reported in Figure S1-S2 in positive and negative ion mode, respectively.

### Data pre-processing

The .raw data obtained from the analysis of samples, QCs, and blanks were preprocessed using the software Compound Discoverer version 3.1 (Thermo Fisher Scientific). Feature alignment was obtained by the adaptive curve regression model; whenever the adaptive curve model failed, the linear model was automatically selected instead. Features were aligned and filtered to remove the compounds also present in the blank samples from the real samples and QCs, as they were attributed to either contaminants or carry-over artifacts. QC-based normalization of the features was carried out based on the peak area variations over time due to different instrumental fluctuations. Compound Discoverer allows performing QC-based area correction over time, meaning that for each individual feature, a linear regression of the peak area in the QC samples is built over time. The response of each feature is, in fact, susceptible to peak area enhancement (e.g., carry-over effects) and suppression (e.g., progressive accumulation of dirt on the ion source) over time. Therefore, each linear regression can be corrected so that the slope of each straight curve is zero and, eventually, each feature in the samples is corrected accordingly. Moreover, features not present in all QCs and those whose area in the QC presented a standard deviation higher than 25% were also filtered out. The remaining features undergoing fragmentation in the *identification-only* QC sample runs were exported for statistical analysis.

### Statistical analysis

The peak tables obtained in ESI+ (40 × 2611, samples × variables) and ESI– (40 × 1610) modes, which included the chromatographic areas of the peaks selected as described in Sect. 4.5, were normalized using the urinary creatinine values and then imported in Matlab (version 2019a). All the following steps were performed separately for the two datasets. A principal component analysis (PCA) model was initially built on the autoscaled data. The data points were colored based on their injection order to highlight any sequence effect's possible occurrence. These PCA models were examined to identify the potential occurrence of trends related to the patients' clinical classification.

The PLS-DA algorithm was applied to select the most effective classification variables and calculate the classification efficiency of the PLS-processed and reduced datasets. A repeated double-cross-validation (r-dCV) approach was applied, using an in-home modified version of a protocol previously developed^42,56,57^. In r-dCV, the available data are organized in two nested loops of cross-validation, the outer one, whose samples are left out to mimic an external test set, and the inner one, which is used for model selection and optimization of (meta-)parameters. In the present study, the inner and the outer loops were characterized by 8 (inner) and 10 (outer) deletion groups, respectively. The term repeated suggests that, to avoid a relevant impact of the composition of the cancelation groups on the final model performances, the whole procedure is repeated a stipulated number of times (*runs*, here 30), each time changing the distribution of the individuals within the cross-validation splits. This procedure not only allows the evaluation of classification figures of merit on samples that are external to the model building and model selection stages (i.e., those in the outer loop) but, by involving repeating the dCV calculation multiple times, provides a reliable estimate of their confidence intervals. Therefore, r-dCV approach allowed to deeply investigate the collected data and, at the current stage, no external validation of the developed models was performed to avoid any data interpretation bias related to population heterogeneity. Moreover, as further validation, to rule out the possibility of obtaining good classification results just because of chance correlations, permutation tests (with 1000 randomizations)^57^ were used to non-parametrically evaluate the null distribution of the main classification figures of merit, to be able to assess their statistical significance and, if needed, obtaining corresponding p-values. In this context, identification of potential biomarkers was conducted through the following filter variable selection strategy based on the calculation of rank product (RP) and further comparison with VIP scores. Having implemented an r-dCV with 30 runs and 8 cancelation groups in the inner loop, a total of 240 models have been built on each data set. At the end of each model computation, a rank label was attributed to each variable depending on its contribution to the model, evaluated based on the absolute value of its associated PLS regression coefficient, the most contributing predictor being given a rank of 1 and so on. Then, for each variable, the overall contribution to the 240 calculated models was summarized by its rank product (RP), i.e., the geometric mean of its ranks across all the sub-models. Accordingly, variables were sorted in increasing order of RP and all the ones having a value lower than the geometric mean of the RP across all the predictors were identified as significant and selected as putative biomarkers. As a further form of validation, the selected variables were compared to those identified as relevant based on the calculation of the VIP scores^58^, and only the matching ones were retained. The selected variables were then allegedly identified using their MS/MS spectra. For metabolites present in the mzCloud database, MS/MS spectra matching was automatically performed by Compound Discoverer software. All other metabolites were tentatively identified by matching the experimental MS and MS/MS spectra to the available spectral libraries, spectra reported in the literature, and the predicted spectra reported in the Human Metabolome Database (HMDB)^59^. Identification data are reported in Supplementary Material Table S4 and S5 for ESI+ and ESI–, respectively. All the variables corresponding to exogenous metabolites or not identified were discarded. The final dimensions of ESI+ and ESI– datasets were 40 × 22 and 40 × 47, respectively. Hence, new r-dCV PLS-DA models were computed, using the two datasets (ESI+ and ESI–) independently and then merging them.

### Ethics approval

The subjects involved in this study were recruited in the Department of Urology at the San Luigi Hospital of Orbassano (TO, Italy), after approval from the reference Ethical Committee (protocol number 17942).

## Conclusions

The wide variety of biomarkers proposed in the scientific literature to provide a precocious diagnosis of prostate cancer somehow demonstrates that none of them fully attains the proposed objective. Several combinations of multiple biomarkers may improve the overall diagnostic efficiency of single metabolites, primarily if a multivariate interpretation of their results is accomplished. However, clear comprehension of the underlying biochemical processes that generate their variety is still missing. The actual perspective goal of metabolomics approaches is to identify multiple biomarkers; the present study is intended to recognize large sets of urinary metabolites whose average concentration is significantly modified by the onset of the neoplastic pathology.

The UHPLC-HRMS approach, data treatment, and chemometric interpretation developed in this study proved to achieve the planned task of identifying a large number of potential PCa biomarkers, even using a limited number of samples to discriminate PCa from BPH patients. The whole procedure of MS data filtration, variable selection, and PLS-DA classification modeling with repeated-cross validation progressively reduced the 2611 and 1610 metabolites initially selected from ESI+ and ESI− data, respectively, to the final sets of 22 and 47 alleged biomarkers, most of which has been hypothetically identified by careful comparison with libraries, literature data, predicted high- and low-resolution mass spectra, and theoretical fragmentation rules for structure-related classes of compounds, i.e., peptides and carnitines. Notably, some of these substances have been filtered out from the noisy backgrounds of both ESI+ and ESI− chromatograms, underlining their relevance in the discrimination of PCa from BPH patients. Further confirmations of the effectiveness of the chemometric approach developed in this study rely on the coherence of PCA and PLS-DA modeling outcomes and the stability of these results under the iterative r-dCV procedure. A final notable consideration is that several identified PCa alleged biomarkers of the same or different classes support the hypothetical neoplastic activation of fewer merging biochemical processes, such as the accelerated metabolism of protecting substances (i.e., ferulic acid) and the altered biosynthesis of steroid hormones.

## Supplementary Information

Supplementary Information 1.

Supplementary Information 2.

Supplementary Table S4.

Supplementary Table S5.

Supplementary Table S6.
