# Supplementary material for: Comprehensive biomarker profiles and chemometric filtering of urinary metabolomics for effective discrimination of prostate carcinoma from benign hyperplasia
Source: Sci Rep. 2022 Mar 14;12:4361. doi: 10.1038/s41598-022-08435-2 (PMC8921285; doi:10.1038/s41598-022-08435-2)
Supplement: Supplementary file 1 — Supplementary Information 1. [file 41598_2022_8435_MOESM1_ESM.docx]

**Supplementary Materials**

**Comprehensive biomarker profiles and chemometric filtering of urinary metabolomics for effective discrimination of prostate carcinoma from benign hyperplasia**

Authors: Eleonora Amante^1,ǁ^, Andrea Cerrato^2,ǁ^_,_ , Eugenio Alladio^1,3^, Anna Laura Capriotti^2,*^, Chiara Cavaliere^2^, Federico Marini^2^ , Carmela Maria Montone^2^, Susy Piovesana^2^, Aldo Laganà^2,4^, Marco Vincenti^1,3^

^1^ Department of Chemistry, University of Turin, Via P. Giuria 7, 10125 Turin, Italy

^2^ Department of Chemistry, Sapienza University of Rome, Piazzale Aldo Moro 5, 00185 Rome, Italy

^3^ Centro Regionale Antidoping e di Tossicologia “A. Bertinaria”, Orbassano, Turin, Italy

^4^ CNR NANOTEC, Campus Ecotekne, University of Salento, Via Monteroni, 73100 Lecce, Italy

^ǁ^ These authors contributed equally.

***Corresponding author**

Department of Chemistry

Università di Roma “La Sapienza”

Piazzale Aldo Moro 5

00185 Rome, Italy

E-mail: [annalaura.capriotti@uniroma1.it](mailto:annalaura.capriotti@uniroma1.it)

tel: +39 06 4991 3945


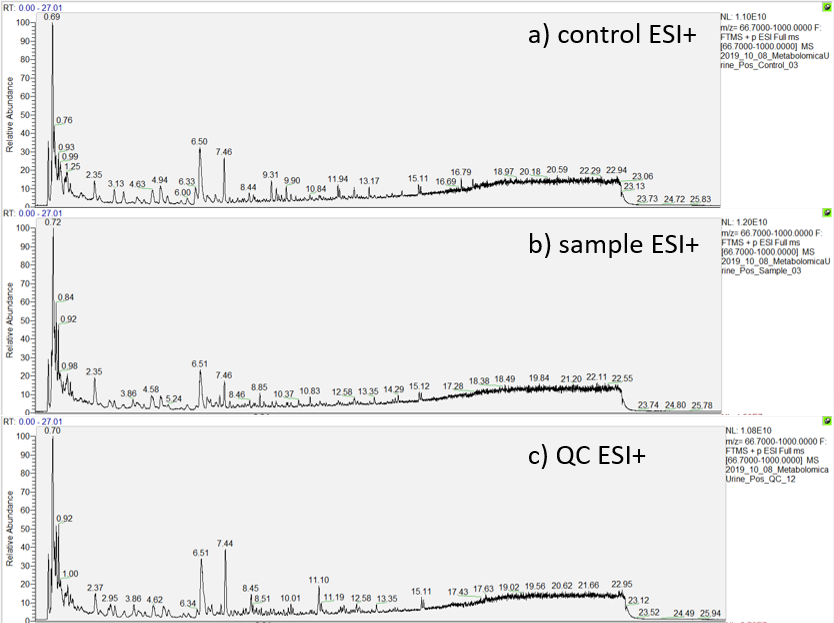


**Figure S1:** Exemplary TIC chromatograms of a control (a), sample (b), and QC (b) acquirated in positive ion mode.


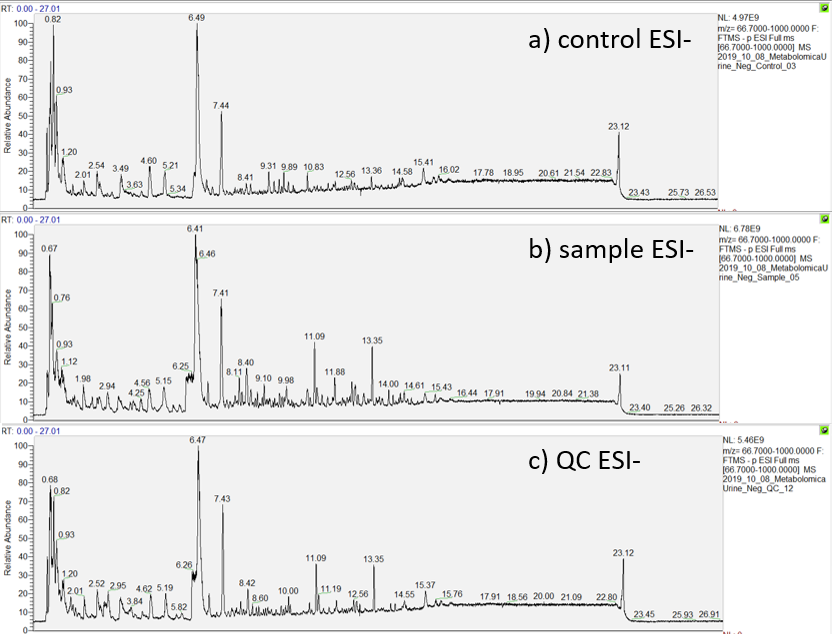


**Figure S2:** Exemplary TIC chromatograms of a control (a), sample (b), and QC (c) acquirated in negative ion mode.


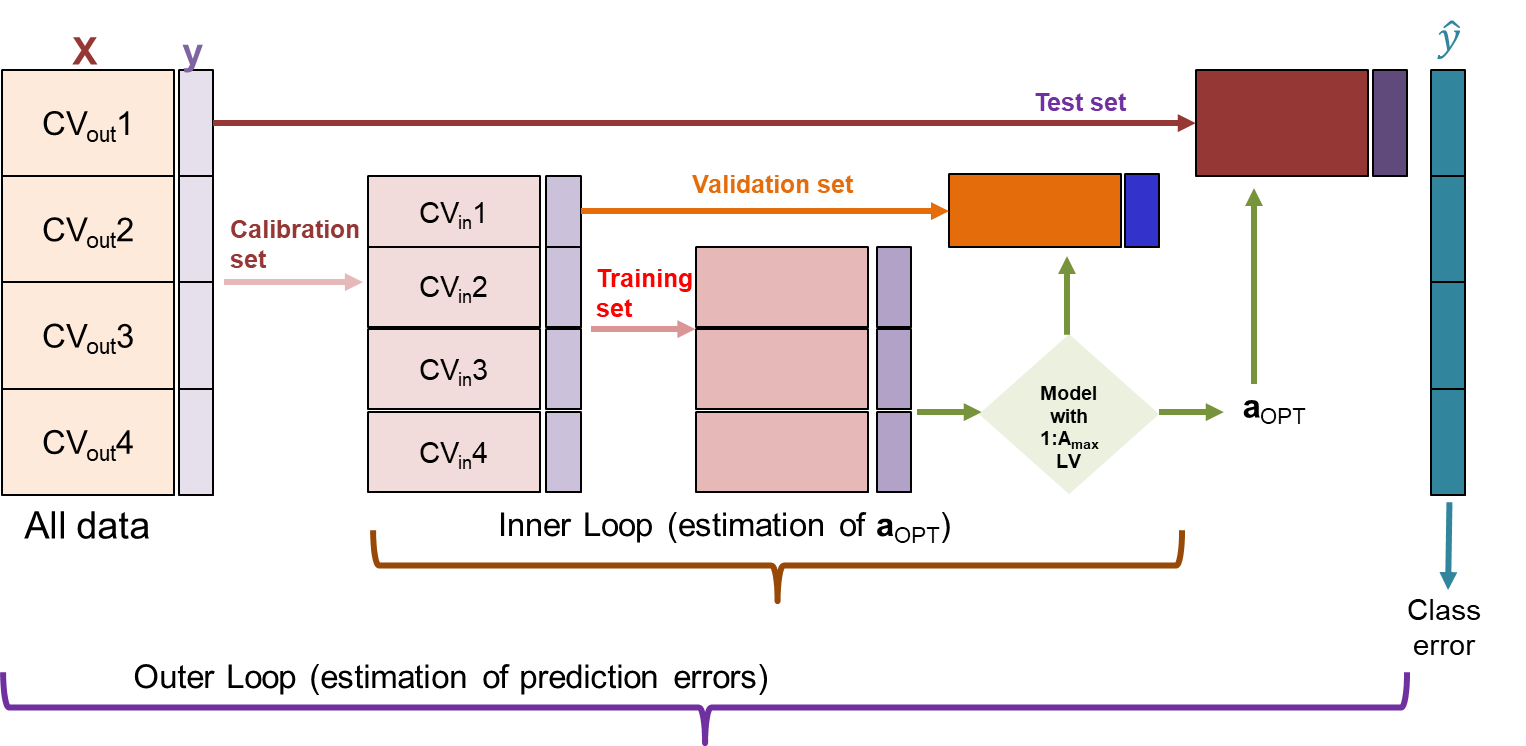


**Figure S3:** Graphical representation of the double cross-validation process. «aOPT» is the optimal number of latent variables selected to build the model. Amax is the maximum number of latent variables used to construct the model. The repetition of this process (with randomization of the outer and inner groups) originates the repeated double cross-validation process.


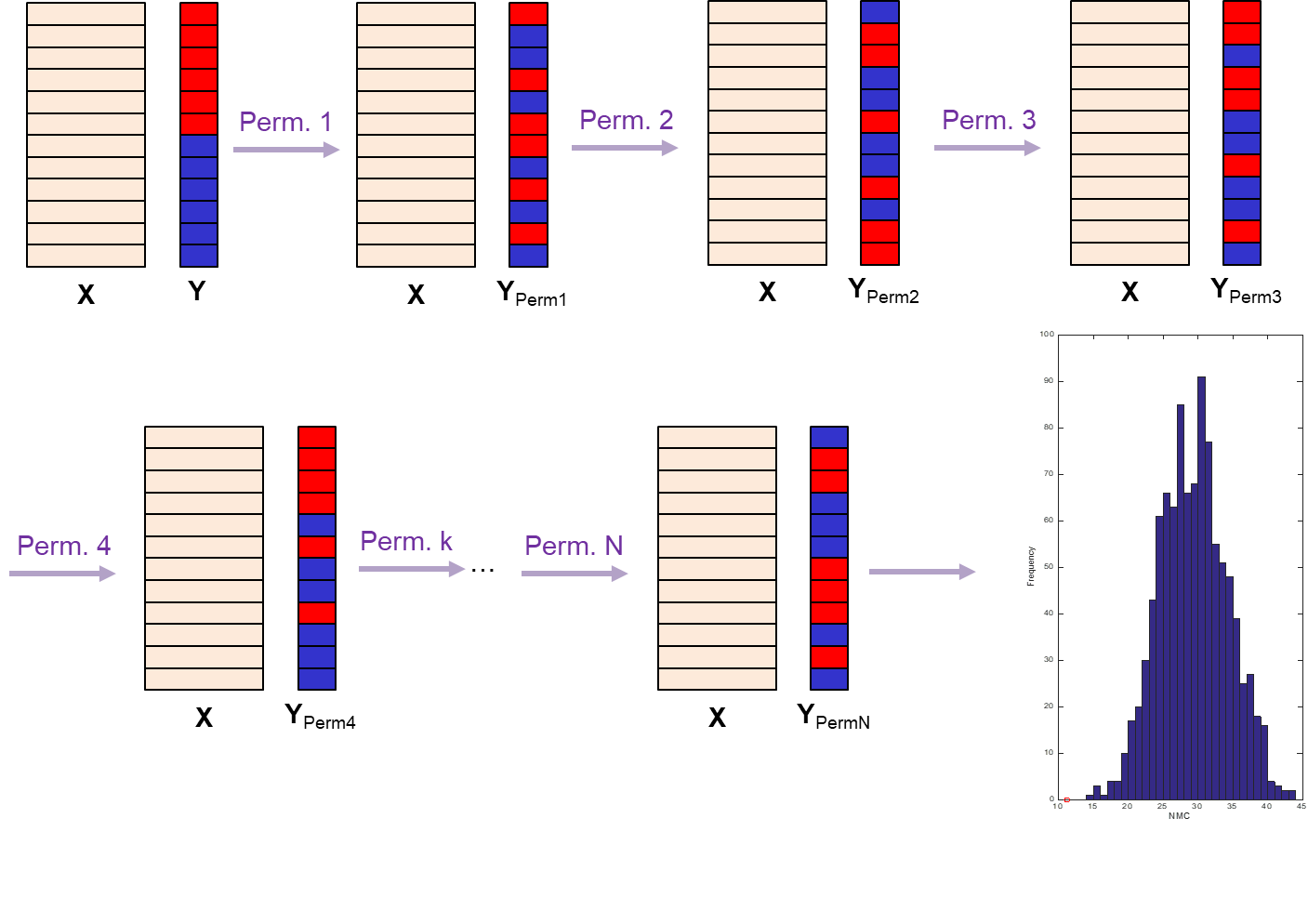


**Figure S4**: Graphical representation of a permutation test. It consists of the class vector's random permutation followed by the computation and validation of the classification model. After having performed this process a reasonable number of times, the distribution of the null hypothesis (i.e., “has the classifier found a significant class structure, that is, a real connection between the data and the class labels?”) is generated. It is finally compared with the outcomes obtained on the real data. ”NMC” on the x-axis in the figure is the number of misclassifications. Further details can be found at doi 10.1007/s11306-011-0330-3.


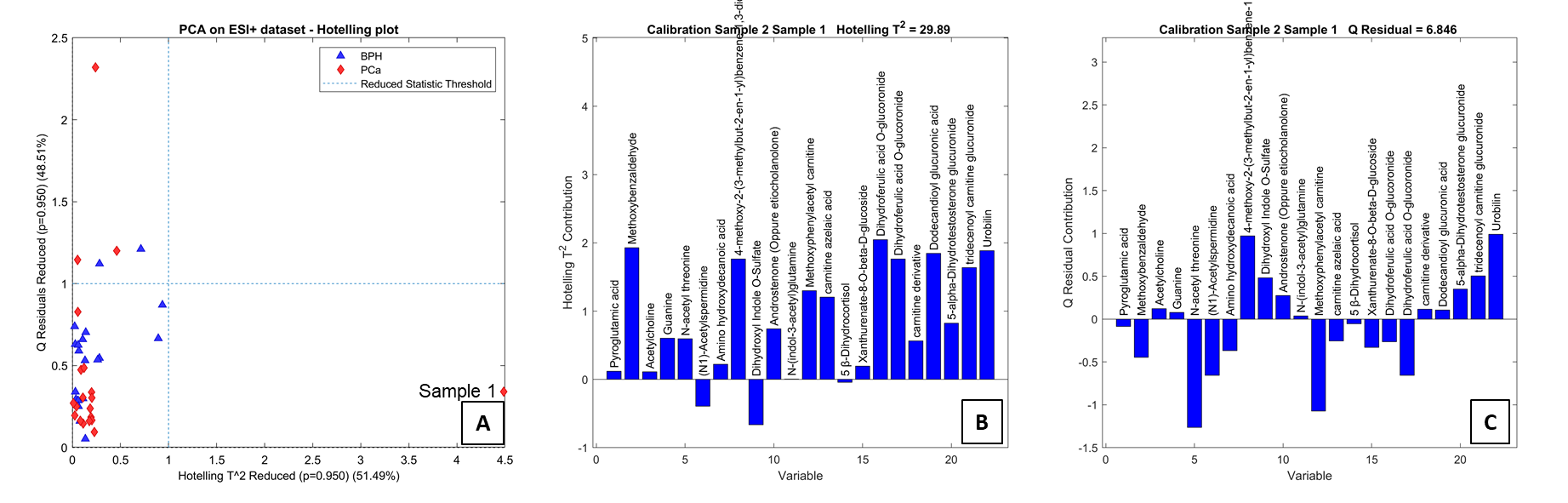


**Figure S5**: (A) Hotelling’s plot for the PCA computed on the ESI+ dataset after variables selection. (B) and (C) represent the contribution plots in T^2^ and Q for Sample 1, respectively.


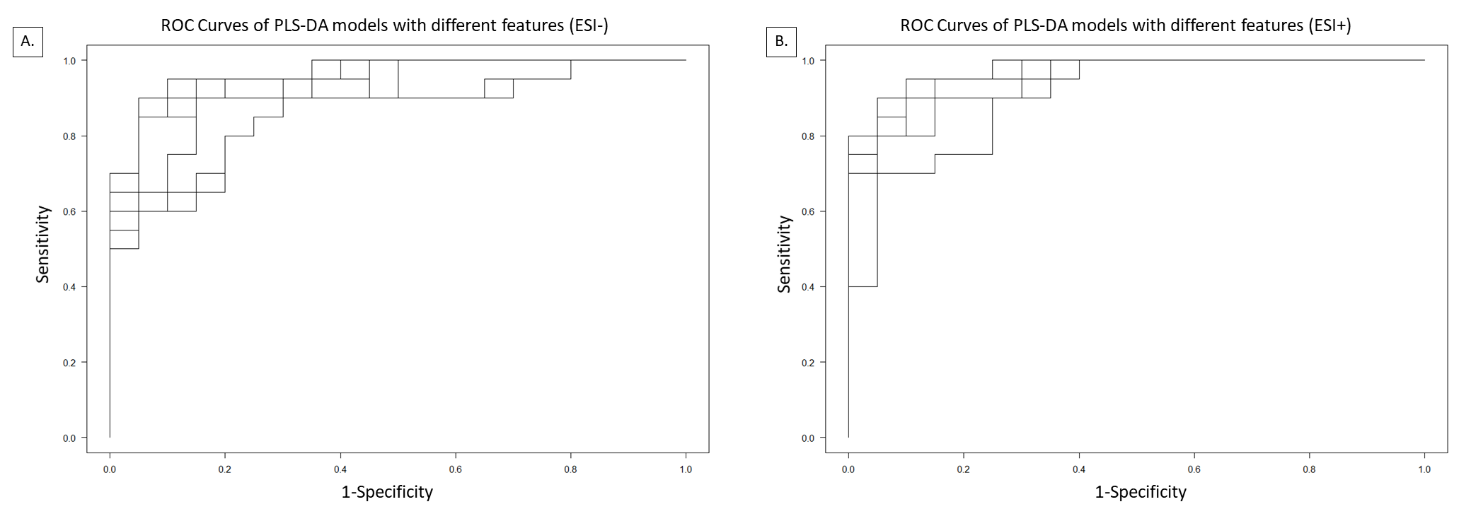


**Figure S6.** ROC Curves for the PLS-DA models obtained using an iterative forward selection approach on the VIP scores from ESI- (A) and ESI + (B) datasets

**Table S1**: Classification performances of individual metabolites (ESI+ data set) as evaluated through the same rDCV procedure as the multivariate models. Results refer to the outer loop (external validation) samples.

| **Metabolite** | **Sensitivity (%)** | **Specificity**  **(%)** | **Classification error (%)** | **Accuracy (%)** | **AUROC** |
| --- | --- | --- | --- | --- | --- |
| Pyroglutamic acid | 48.9±3.5 | 82.3±3.1 | 34.4±2.5 | 65.6±2.5 | 0.720±0.021 |
| Methoxy benzaldehyde | 70.0±3.4 | 35.9±1.9 | 47.1±1.9 | 52.9±1.9 | 0.460±0.075 |
| Acetylcholine | 57.7±2.5 | 75.0±0.0 | 33.6±1.3 | 66.3±1.3 | 0.643±0.025 |
| Guanine | 65.0±0.0 | 79.3±1.8 | 27.9±0.9 | 72.2±0.9 | 0.780±0.018 |
| N-acetyl threonine | 85.0±0.0 | 25.8±3.3 | 44.6±1.6 | 55.4±1.6 | 0.455±0.102 |
| (N1)-Acetylspermidine | 67.1±2.5 | 80.1±1.9 | 26.4±1.4 | 73.6±1.4 | 0.846±0.014 |
| Amino hydroxydecanoic acid | 65.0±0.0 | 74.4±1.6 | 30.3±0.8 | 69.7±0.8 | 0.744±0.015 |
| 4-methoxy-2-(3-methylbut-2-en-1-yl)benzene-1,3-diol | 80.2±2.8 | 19.9±3.4 | 50.0±2.2 | 50.0±2.2 | 0.674±0.103 |
| Dihydroxyl Indole O-Sulfate | 50.0±0.0 | 80.6±4.5 | 34.7±2.2 | 65.3±2.2 | 0.655±0.027 |
| Androstenone/Etiocholanolone | 60.3±2.3 | 84.5±1.5 | 27.6±1.6 | 72.4±1.6 | 0.698±0.022 |
| N-(indol-3-acetyl) glutamine | 58.6±2.3 | 75.0±0.0 | 33.2±1.1 | 66.8±1.1 | 0.709±0.018 |
| Methoxyphenylacetyl carnitine | 74.3±1.8 | 35.8±3.7 | 44.9±2.2 | 55.0±2.2 | 0.511±0.095 |
| Carnitine azelaic acid | 67.6±2.5 | 43.2±3.3 | 44.6±2.0 | 55.4±2.0 | 0.511±0.043 |
| Dihydrocortisol | 68.6±2.3 | 83.5±2.3 | 23.9±1.7 | 76.0±1.7 | 0.810±0.016 |
| Xanthurenate-8-O-beta-D-glucoside | 59.4±1.6 | 65.7±1.8 | 37.5±1.2 | 62.5±1.2 | 0.677±0.021 |
| dihydro(iso)ferulic acid glucuronide | 84.9±0.7 | 39.9±0.7 | 37.6±0.7 | 62.4±0.7 | 0.509±0.037 |
| dihydro(iso)ferulic acid glucuronide | 83.4±2.4 | 35.0±0.0 | 40.8±1.2 | 59.2±1.2 | 0.522±0.044 |
| Carnitine derivative | 43.3±3.1 | 93.3±2.4 | 31.7±2.0 | 68.3±2.0 | 0.564±0.041 |
| Dodecanedioyl glucuronic acid | 75.2±1.0 | 35.8±3.8 | 44.5±2.0 | 55.5±2.0 | 0.503±0.052 |
| 5-alpha-Dihydrotestosterone glucuronide | 62.5±3.1 | 85.0±0.0 | 26.3±1.5 | 73.8±1.5 | 0.703±0.024 |
| Tridecenoyl carnitine glucuronide | 76.7±3.1 | 43.5±2.3 | 39.9±2.3 | 60.1±2.3 | 0.512±0.071 |
| Urobilin | 83.2±2.8 | 30.9±1.9 | 42.9±1.6 | 57.0±1.6 | 0.414±0.052 |

**Table S2**: Classification performances of individual metabolites (ESI- data set) as evaluated through the same rDCV procedure as the multivariate models. Results refer to the outer loop (external validation) samples.

| **Metabolite** | **Sensitivity (%)** | **Specificity**  **(%)** | **Classification error (%)** | **Accuracy (%)** | **AUROC** |
| --- | --- | --- | --- | --- | --- |
| Malic Acid | 57.5±2.5 | 87.7±3.1 | 27.4±2.1 | 72.6±2.1 | 0.750±0.034 |
| Imidazolelactic acid | 64.9±0.7 | 70.0±0.0 | 32.5±0.4 | 67.5±0.4 | 0.681±0.019 |
| Hexanoylglycine | 69.6±3.6 | 42.7±3.8 | 43.9±2.5 | 56.1±2.5 | 0.578±0.062 |
| Dihydroxy-5-methylthio-4-pentenoic acid (DMTPA) | 56.4±2.5 | 82.9±2.9 | 30.4±2.1 | 69.7±2.1 | 0.728±0.016 |
| Sulfooxybutanoic acid | 56.3±2.2 | 90.0±0.0 | 26.9±1.1 | 73.2±1.1 | 0.652±0.020 |
| Heptanoylglycine | 73.7±2.4 | 40.0±0.0 | 43.2±1.2 | 56.8±1.2 | 0.541±0.058 |
| N-lactoylvaline | 60.0±0.0 | 74.9±1.9 | 32.5±0.9 | 67.5±0.9 | 0.527±0.093 |
| Phenylacetylglycine | 35.0±0.0 | 79.3±1.8 | 42.9±0.9 | 57.1±0.9 | 0.552±0.082 |
| Ethylphenyl sulfate | 37.6±2.5 | 85.0±0.0 | 38.7±1.3 | 61.3±1.3 | 0.567±0.045 |
| 3-hydroxy-3-(4-hydroxy-3-methoxyphenyl) propanoic acid | 81.1±2.5 | 32.6±2.5 | 43.2±1.5 | 56.8±1.5 | 0.459±0.046 |
| [2-hydroxy-5-(prop-2-en-1-yl)phenyl]oxidanesulfonic acid | 40.5±1.5 | 79.8±1.0 | 39.8±0.9 | 60.2±0.9 | 0.530±0.040 |
| 5-Aminoimidazole-4-carboxamide glutaric acid | 50.3±1.2 | 70.0±0.0 | 39.8±0.6 | 60.2±0.6 | 0.618±0.034 |
| Indolylacryloylglycine | 41.7±2.6 | 89.7±1.2 | 34.3±1.3 | 65.7±1.3 | 0.645±0.040 |
| 2-[4-hydroxy-3-(sulfooxy) phenyl]acetic acid | 50.0±0.0 | 70.0±0.0 | 40.0±0.0 | 60.0±0.0 | 0.631±0.029 |
| Benzoyl glutamic acid | 25.1±0.7 | 79.8±2.0 | 47.6±1.1 | 52.4±1.1 | 0.474±0.119 |
| Propyl Hydroxyhippuric acid | 59.2±3.7 | 75.0±0.0 | 32.9±1.8 | 67.1±1.8 | 0.695±0.017 |
| 5-(Hydroxyphenyl)-gamma-valerolactone-O-sulphate | 25.9±1.9 | 89.3±1.8 | 42.4±1.3 | 57.6±1.3 | 0.414±0.056 |
| Hydroxybutyric acid glucuronide | 43.9±3.8 | 78.1±2.5 | 39.0±2.3 | 61.0±2.3 | 0.558±0.030 |
| Methylguanosine | 70.5±1.5 | 70.0±0.0 | 29.8±0.8 | 70.2±0.8 | 0.737±0.014 |
| 4-Methylcatechol O-glucuronide | 45.0±0.0 | 83.7±2.2 | 35.6±1.1 | 64.3±1.1 | 0.606±0.037 |
| Octanedioyl glutamine | 52.7±10.7 | 39.1±7.1 | 54.1±7.3 | 45.9±7.3 | 0.774±0.068 |
| N-(indol-3-acetyl) glutamine | 60.0±0.0 | 80.0±0.0 | 30.0±0.0 | 70.0±0.0 | 0.756±0.021 |
| Succinyl Tryptophan | 50.0±0.0 | 85.0±0.0 | 32.5±0.0 | 67.5±0.0 | 0.696±0.028 |
| 2-Methoxy-4-vinylphenol glucuronide | 34.3±4.0 | 74.2±3.3 | 45.7±3.1 | 54.2±3.1 | 0.553±0.108 |
| Pyr-Xle-Ser | 62.3±2.7 | 88.3±2.8 | 24.7±1.8 | 75.3±1.8 | 0.735±0.017 |
| Hydroxy methoxy indole glucuronide | 39.9±0.7 | 85.0±2.9 | 37.5±1.5 | 62.5±1.5 | 0.581±0.039 |
| Suberoyl glucuronic acid | 63.1±2.8 | 45.2±3.9 | 45.8±2.7 | 54.2±2.7 | 0.566±0.047 |
| alpha-CEHC sulfate | 55.0±0.0 | 75.2±1.0 | 34.9±0.5 | 65.1±0.5 | 0.712±0.021 |
| Feruloyl-quinic acid | 79.9±0.7 | 40.0±0.0 | 40.0±0.4 | 60.0±0.4 | 0.500±0.039 |
| (epi)Catechin sulfate | 90.0±0.0 | 37.5±2.5 | 36.2±1.3 | 63.7±1.3 | 0.497±0.039 |
| dihydro(iso)ferulic acid glucuronide | 90.0±0.0 | 40.0±0.0 | 35.0±0.0 | 65.0±0.0 | 0.537±0.036 |
| dihydro(iso)ferulic acid glucuronide | 83.9±2.3 | 35.4±1.4 | 40.4±1.3 | 59.7±1.3 | 0.507±0.047 |
| dimethylene suberic acid glucoronide | 79.7±1.2 | 40.0±0.0 | 40.1±0.6 | 59.9±0.6 | 0.533±0.041 |
| dimethylene suberic acid glucoronide | 52.5±2.5 | 84.6±1.4 | 31.4±1.5 | 68.5±1.5 | 0.662±0.026 |
| decenedioyl glucuronic acid | 61.1±9.3 | 36.8±5.3 | 51.1±4.9 | 48.9±4.9 | 0.802±0.056 |
| methylcathecol glucuronide sulfate | 48.9±2.1 | 85.0±0.0 | 33.0±1.0 | 67.0±1.0 | 0.669±0.043 |
| Methyl(epi)catechin sulfate | 88.8±2.6 | 40.8±1.9 | 35.2±1.6 | 64.8±1.6 | 0.491±0.026 |
| Hydroxyandrosterone sulfate isomer | 52.9±4.9 | 41.6±5.6 | 52.8±4.0 | 47.2±4.0 | 0.654±0.061 |
| Dodecanedioyl glucuronic acid | 71.3±2.2 | 40.7±2.7 | 44.0±1.7 | 56.0±1.7 | 0.517±0.047 |
| Androstenol glucuronide | 39.8±3.2 | 89.5±1.5 | 35.3±1.8 | 64.6±1.8 | 0.552±0.040 |
| alpha-CEHC glucuronide | 55.1±0.7 | 64.0±2.0 | 40.5±1.1 | 59.6±1.1 | 0.637±0.023 |
| Uroerythrin (biotrypirrin A) | 58.7±3.0 | 32.5±4.3 | 54.4±3.0 | 45.6±3.0 | 0.645±0.062 |
| (epi)Catechin glucuronide | 80.0±0.0 | 30.2±1.0 | 44.9±0.5 | 55.1±0.5 | 0.451±0.046 |
| Trihydroxyoctadecenoic acid glucuronide | 70.0±0.0 | 39.5±2.9 | 45.2±1.5 | 54.8±1.5 | 0.513±0.052 |
| Trihydroxyoctadecenoic acid glucuronide | 69.9±0.7 | 35.2±1.0 | 47.5±0.6 | 52.5±0.6 | 0.470±0.041 |
| Trihydroxycholanoic acid glucuronide | 38.8±2.2 | 83.0±2.7 | 39.1±1.7 | 60.9±1.7 | 0.522±0.036 |
| (3a,5b,7a,12a)-24-[(carboxymethyl)amino]-1,12-dihydroxy-24-oxocholan-3-yl-b-D-Glucopyranosiduronic acid | 60.0±0.0 | 84.8±1.4 | 27.6±0.7 | 72.4±0.7 | 0.659±0.018 |

**Table S3**: Results (p-values) of the t-tests performed on the compounds of the ESI- and ESI+ datasets. The (*) symbol indicates a significant p-value lower than 0.05.

| **Compound** | **ESI mode** | **p-value** | **Significance** |
| --- | --- | --- | --- |
| Malic Acid | ESI- | 1.078E-02 | * |
| Imidazolelactic acid | ESI- | 1.081E-02 | * |
| Hexanoylglycine | ESI- | 2.911E-01 |  |
| Dihydroxy-5-methylthio-4-pentenoic acid (DMTPA) | ESI- | 1.758E-03 | * |
| Sulfooxybutanoic acid | ESI- | 3.526E-03 | * |
| Heptanoylglycine | ESI- | 2.177E-01 |  |
| N-lactoylvaline | ESI- | 1.482E-01 |  |
| Phenylacetylglycine | ESI- | 2.180E-01 |  |
| Ethylphenyl sulfate | ESI- | 3.260E-02 | * |
| 3-hydroxy-3-(4-hydroxy-3-methoxyphenyl) propanoic acid | ESI- | 1.131E-01 |  |
| [2-hydroxy-5-(prop-2-en-1-yl)phenyl]oxidanesulfonic acid | ESI- | 1.073E-01 |  |
| 5-Aminoimidazole-4-carboxamide glutaric acid | ESI- | 6.956E-02 |  |
| Indolylacryloylglycine | ESI- | 1.546E-02 | * |
| 2-[4-hydroxy-3-(sulfooxy) phenyl]acetic acid | ESI- | 2.697E-02 | * |
| Benzoyl glutamic acid | ESI- | 2.044E-01 |  |
| Propyl Hydroxyhippuric acid | ESI- | 2.049E-02 | * |
| 5-(Hydroxyphenyl)-gamma-valerolactone-O-sulphate | ESI- | 1.361E-01 |  |
| Hydroxybutyric acid glucuronide | ESI- | 5.147E-02 |  |
| Methylguanosine | ESI- | 3.704E-03 | * |
| 4-Methylcatechol O-glucuronide | ESI- | 3.166E-02 | * |
| Octanedioyl glutamine | ESI- | 7.689E-01 |  |
| N-(indol-3-acetyl) glutamine | ESI- | 2.293E-03 | * |
| Succinyl Tryptophan | ESI- | 1.516E-02 | * |
| 2-Methoxy-4-vinylphenol glucuronide | ESI- | 3.002E-01 |  |
| Pyr-Xle-Ser | ESI- | 1.902E-03 | * |
| Hydroxy methoxy indole glucuronide | ESI- | 2.336E-02 | * |
| Suberoyl glucuronic acid | ESI- | 2.864E-01 |  |
| alpha-CEHC sulfate | ESI- | 7.818E-03 | * |
| Feruloyl-quinic acid | ESI- | 6.590E-02 |  |
| (epi)Catechin sulfate | ESI- | 5.131E-02 |  |
| dihydro(iso)ferulic acid glucuronide | ESI- | 3.071E-02 | * |
| dihydro(iso)ferulic acid glucuronide | ESI- | 5.560E-02 |  |
| dimethylene suberic acid glucoronide | ESI- | 2.156E-01 |  |
| dimethylene suberic acid glucoronide | ESI- | 1.606E-02 | * |
| decenedioyl glucuronic acid | ESI- | 7.584E-01 |  |
| methylcathecol glucuronide sulfate | ESI- | 2.904E-02 | * |
| Methyl(epi)catechin sulfate | ESI- | 3.848E-02 | * |
| Hydroxyandrosterone sulfate isomer | ESI- | 5.316E-01 |  |
| Dodecanedioyl glucuronic acid | ESI- | 1.277E-01 |  |
| Androstenol glucuronide | ESI- | 6.106E-02 |  |
| alpha-CEHC glucuronide | ESI- | 2.291E-02 | * |
| Uroerythrin (biotrypirrin A) | ESI- | 4.016E-01 |  |
| (epi)Catechin glucuronide | ESI- | 9.643E-02 |  |
| Trihydroxyoctadecenoic acid glucuronide | ESI- | 1.701E-01 |  |
| Trihydroxyoctadecenoic acid glucuronide | ESI- | 1.277E-01 |  |
| Trihydroxycholanoic acid glucuronide | ESI- | 6.086E-02 |  |
| (3a,5b,7a,12a)-24-[(carboxymethyl)amino]-1,12-dihydroxy-24-oxocholan-3-yl-b-D-Glucopyranosiduronic acid | ESI- | 8.882E-03 | * |
| Pyroglutamic acid | ESI+ | 5.919E-03 | * |
| Methoxy benzaldehyde | ESI+ | 1.713E-01 |  |
| Acetylcholine | ESI+ | 6.764E-03 | * |
| Guanine | ESI+ | 2.095E-03 | * |
| N-acetyl threonine | ESI+ | 2.181E-01 |  |
| (N1)-Acetylspermidine | ESI+ | 2.907E-04 | * |
| Amino hydroxydecanoic acid | ESI+ | 1.432E-03 | * |
| 4-methoxy-2-(3-methylbut-2-en-1-yl)benzene-1,3-diol | ESI+ | 3.391E-01 |  |
| Dihydroxyl Indole O-Sulfate | ESI+ | 7.618E-03 | * |
| Androstenone/Etiocholanolone | ESI+ | 1.035E-02 | * |
| N-(indol-3-acetyl) glutamine | ESI+ | 3.033E-03 | * |
| Methoxyphenylacetyl carnitine | ESI+ | 1.963E-01 |  |
| Carnitine azelaic acid | ESI+ | 1.261E-01 |  |
| Dihydrocortisol | ESI+ | 3.041E-04 | * |
| Xanthurenate-8-O-beta-D-glucoside | ESI+ | 6.339E-03 | * |
| dihydro(iso)ferulic acid glucuronide | ESI+ | 5.036E-02 |  |
| dihydro(iso)ferulic acid glucuronide | ESI+ | 5.654E-02 |  |
| Carnitine derivative | ESI+ | 5.496E-02 |  |
| Dodecanedioyl glucuronic acid | ESI+ | 1.230E-01 |  |
| 5-alpha-Dihydrotestosterone glucuronide | ESI+ | 1.235E-02 | * |
| Tridecenoyl carnitine glucuronide | ESI+ | 2.088E-01 |  |
| Urobilin | ESI+ | 1.373E-01 |  |
