# Supplementary material for: Comprehensive biomarker profiles and chemometric filtering of urinary metabolomics for effective discrimination of prostate carcinoma from benign hyperplasia
Source: Sci Rep. 2022 Mar 14;12:4361. doi: 10.1038/s41598-022-08435-2 (PMC8921285; doi:10.1038/s41598-022-08435-2)
Supplement: Supplementary file 2 — Supplementary Information 2. [file 41598_2022_8435_MOESM2_ESM.docx]

**Supplementary Material**

**Comprehensive biomarker profiles and chemometric filtering of urinary metabolomics for effective discrimination of prostate carcinoma from benign hyperplasia**

Authors: Eleonora Amante^1,ǁ^, Andrea Cerrato^2,ǁ^_,_ , Eugenio Alladio^1,3^, Anna Laura Capriotti^2,*^, Chiara Cavaliere^2^, Federico Marini^2^ , Carmela Maria Montone^2^, Susy Piovesana^2^, Aldo Laganà^2,4^, Marco Vincenti^1,3^

^1^ Department of Chemistry, University of Turin, Via P. Giuria 7, 10125 Turin, Italy

^2^ Department of Chemistry, Sapienza University of Rome, Piazzale Aldo Moro 5, 00185 Rome, Italy

^3^ Centro Regionale Antidoping e di Tossicologia “A. Bertinaria”, Orbassano, Turin, Italy

^4^ CNR NANOTEC, Campus Ecotekne, University of Salento, Via Monteroni, 73100 Lecce, Italy

^ǁ^ These authors contributed equally.

***Corresponding author**

Department of Chemistry

Università di Roma “La Sapienza”

Piazzale Aldo Moro 5

00185 Rome, Italy

E-mail: [annalaura.capriotti@uniroma1.it](mailto:annalaura.capriotti@uniroma1.it)


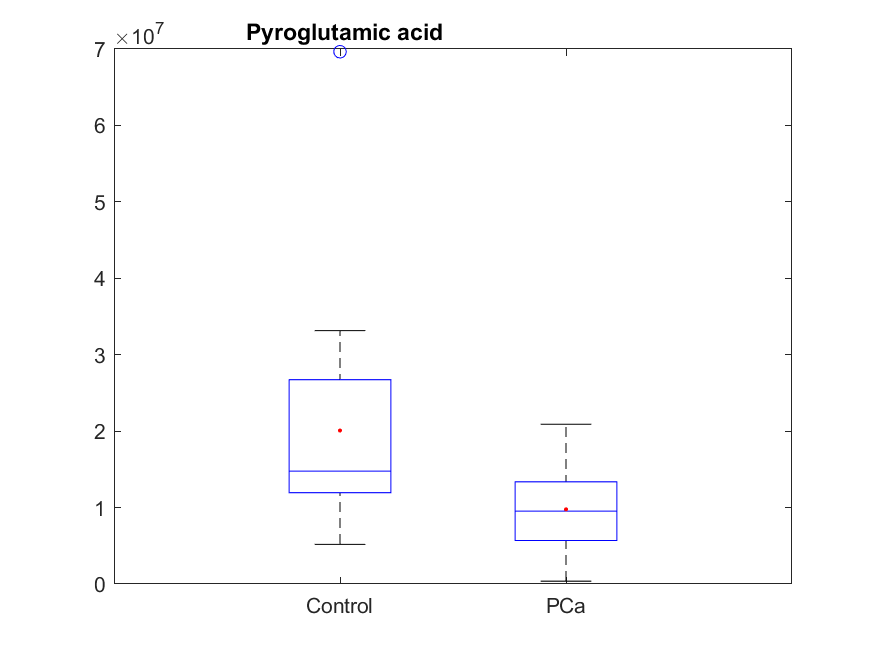


**Figure S6.** Box plot for compound 1 ESI+ (pyroglutamic acid)


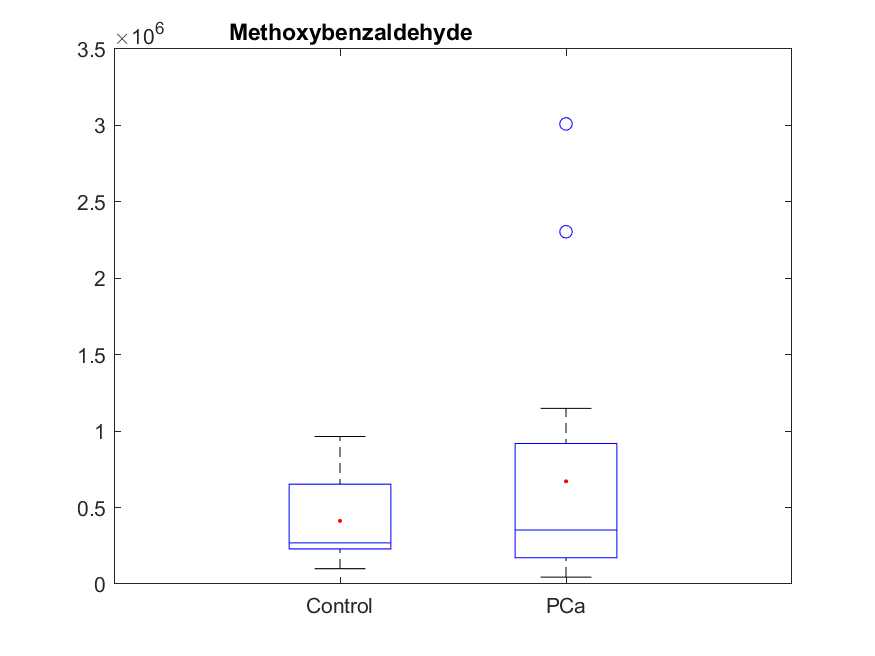


**Figure S7.** Box plot for compound 2 ESI+ (methoxybenzaldehyde)


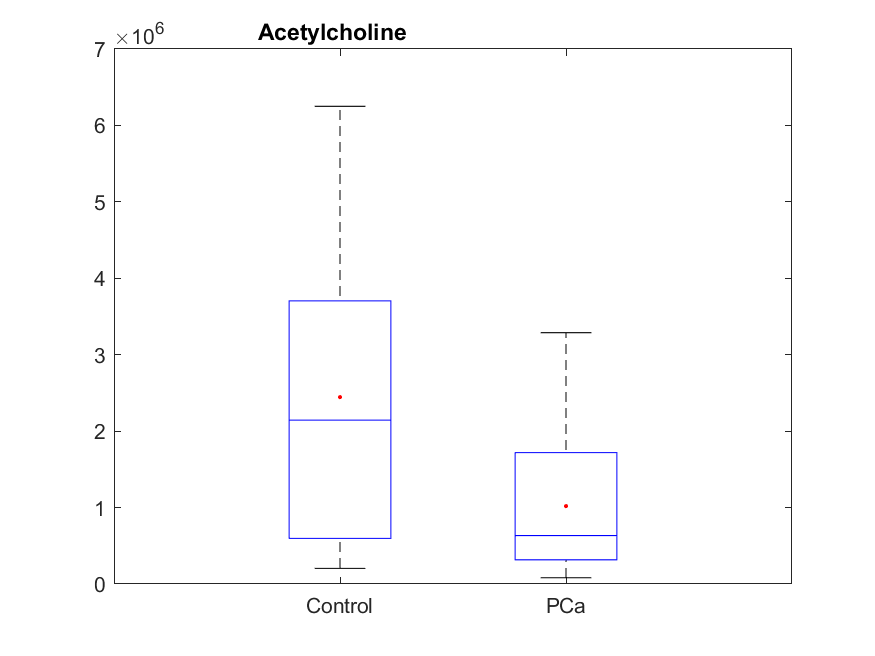


**Figure S8.** Box plot for compound 3 ESI+ (acetylcholine)


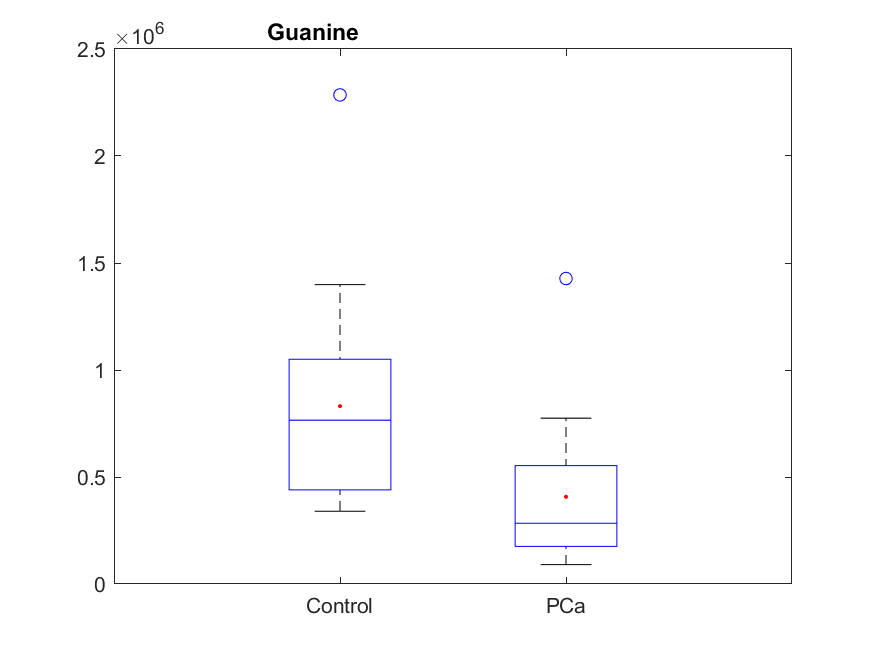


**Figure S9.** Box plot for compound 4 ESI+ (guanine)


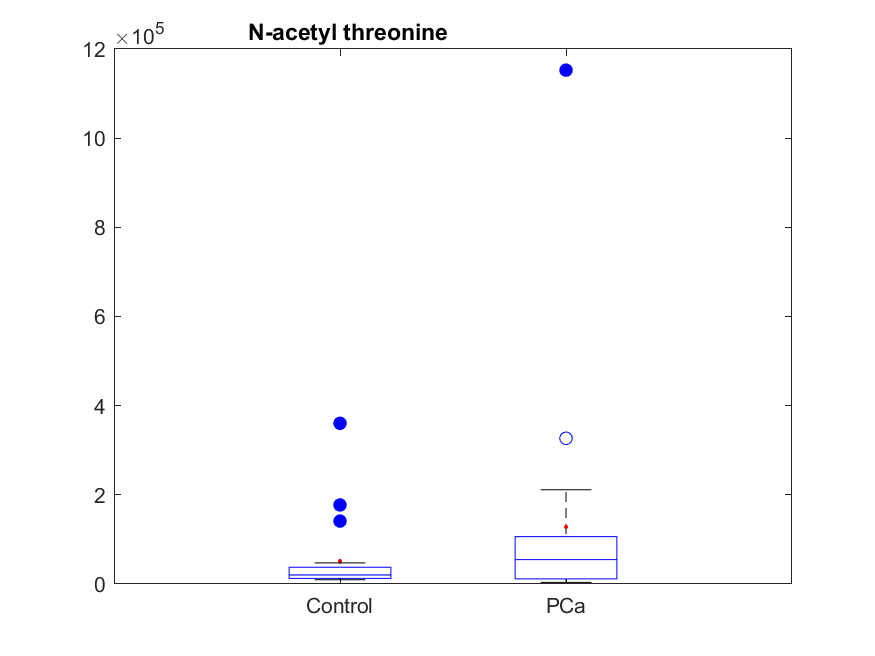


**Figure S10.** Box plot for compound 5 ESI+ (N-acetyl threonine)


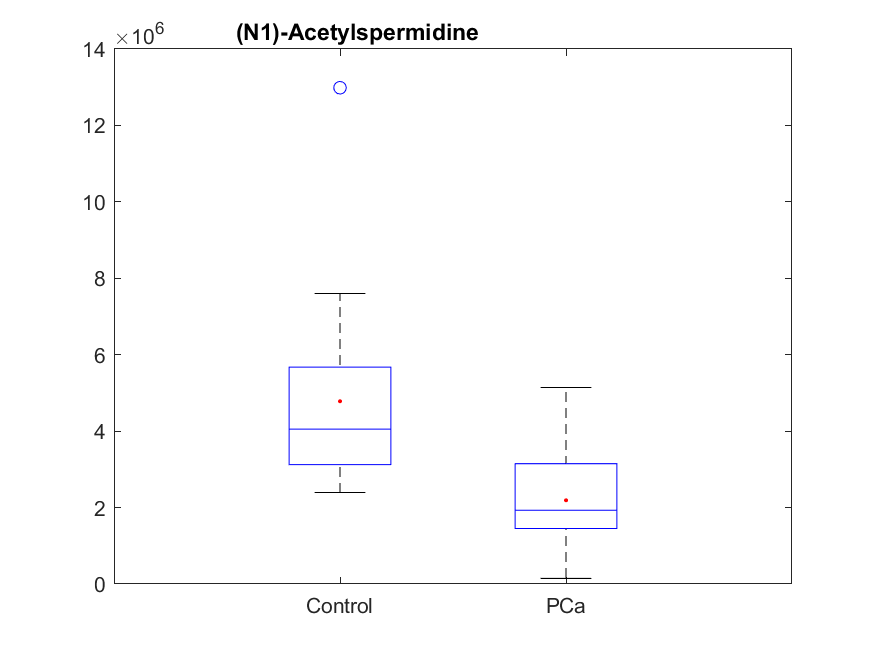


**Figure S11.** Box plot for compound 6 ESI+ ((N1)-Acetylspermidine)


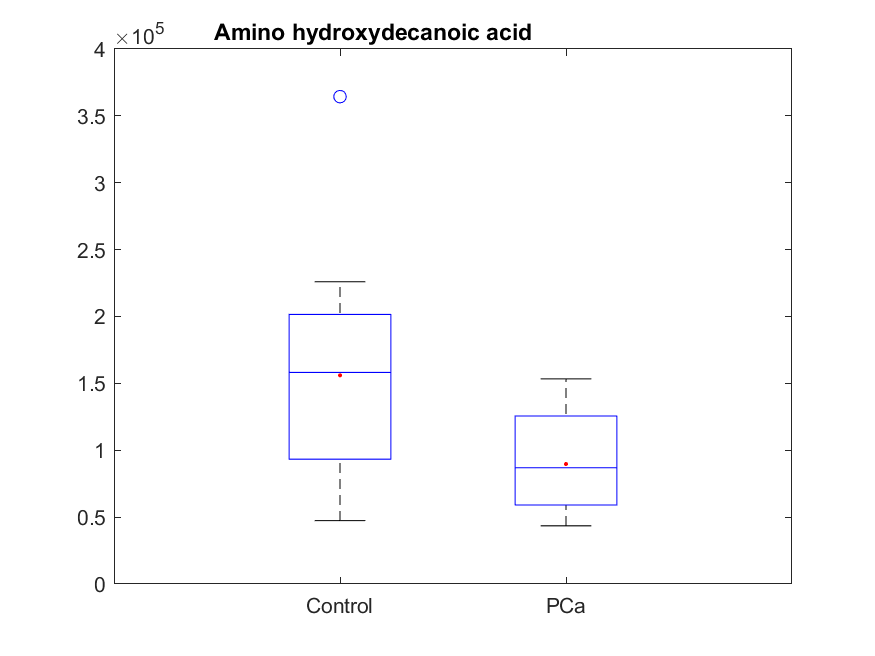


**Figure S12.** Box plot for compound 7 ESI+ (Amino hydroxydecanoic acid)


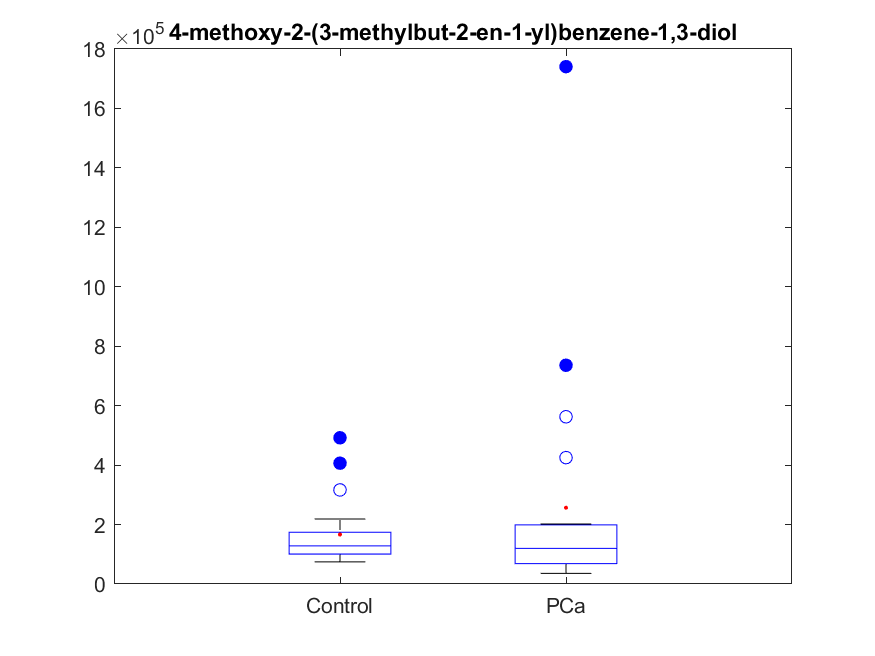


**Figure S13.** Box plot for compound 8 ESI+ (4-methoxy-2-(3-methylbut-2-en-1-yl)benzene-1,3-diol)


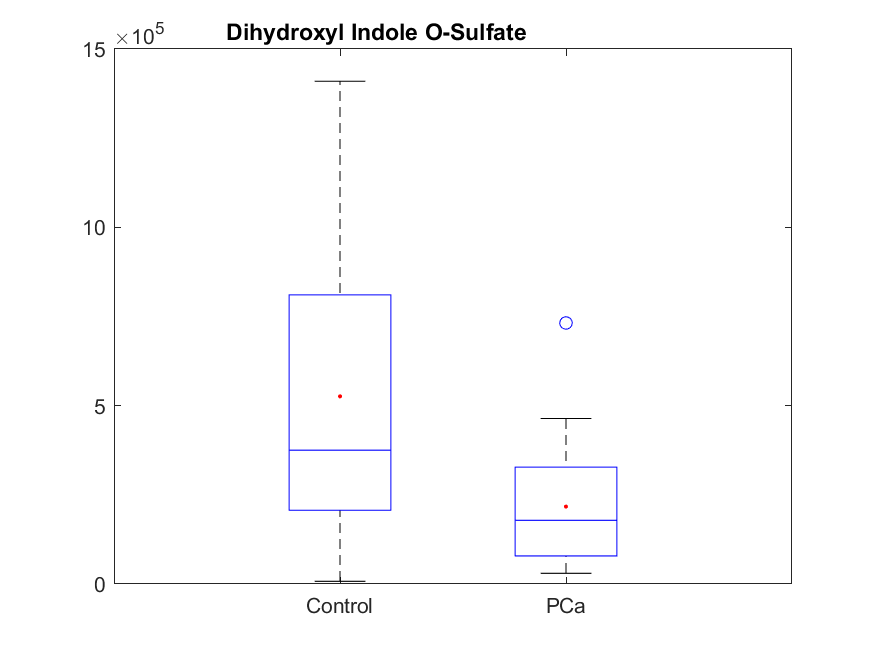


**Figure S14.** Box plot for compound 9 ESI+ (Dihydroxyl Indole O-Sulfate)


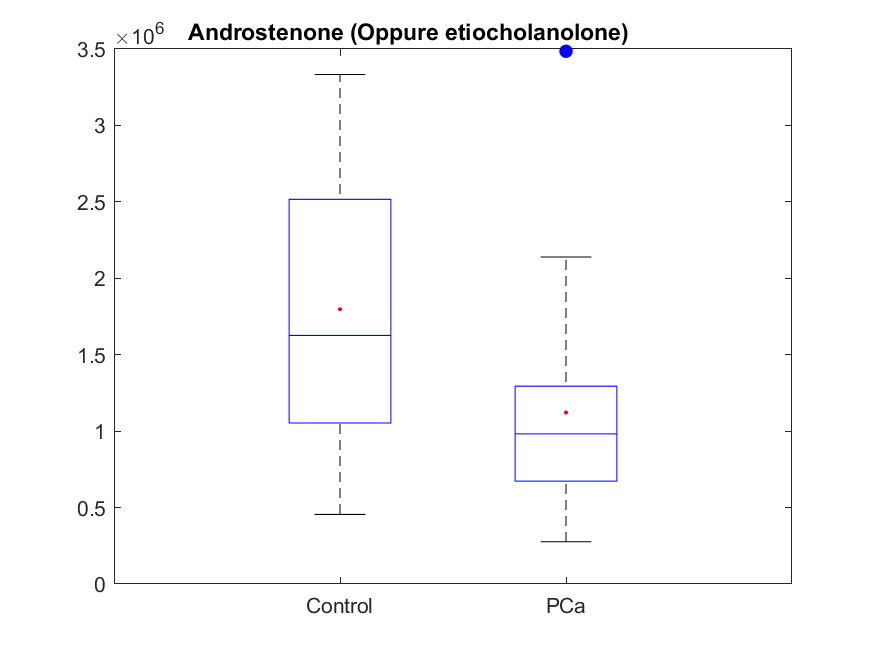


**Figure S15.** Box plot for compound 10 ESI+ (Androstenone/Etiocholanolone)


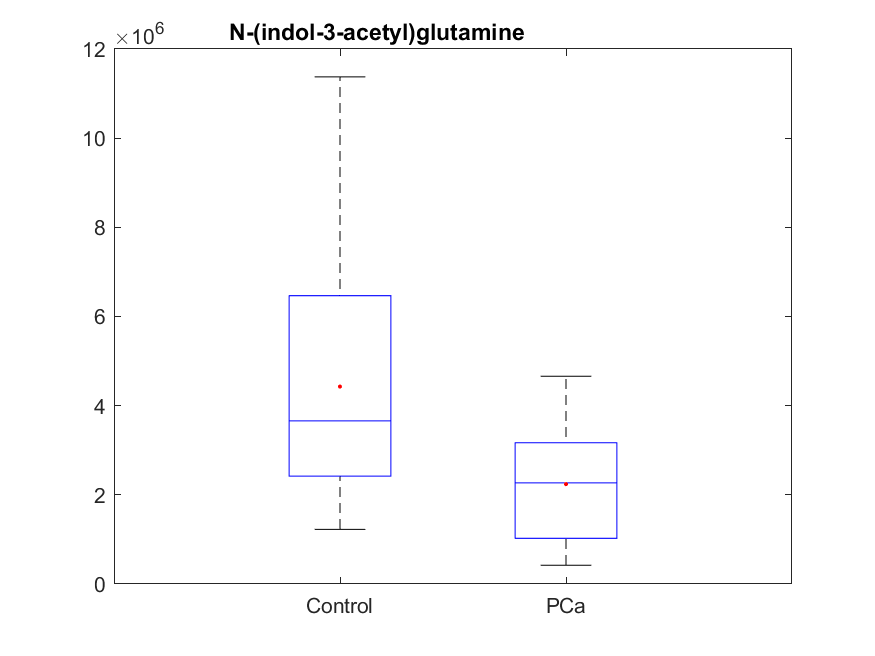


**Figure S16.** Box plot for compound 11 ESI+ (N-(indol-3-acetyl) glutamine)


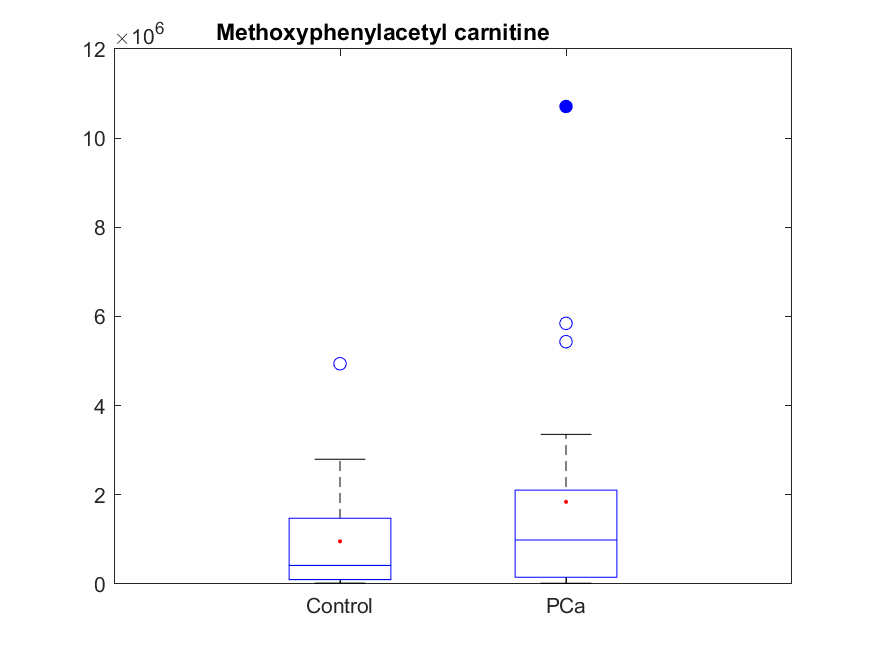


**Figure S17.** Box plot for compound 12 ESI+ (Methoxyphenylacetyl carnitine)


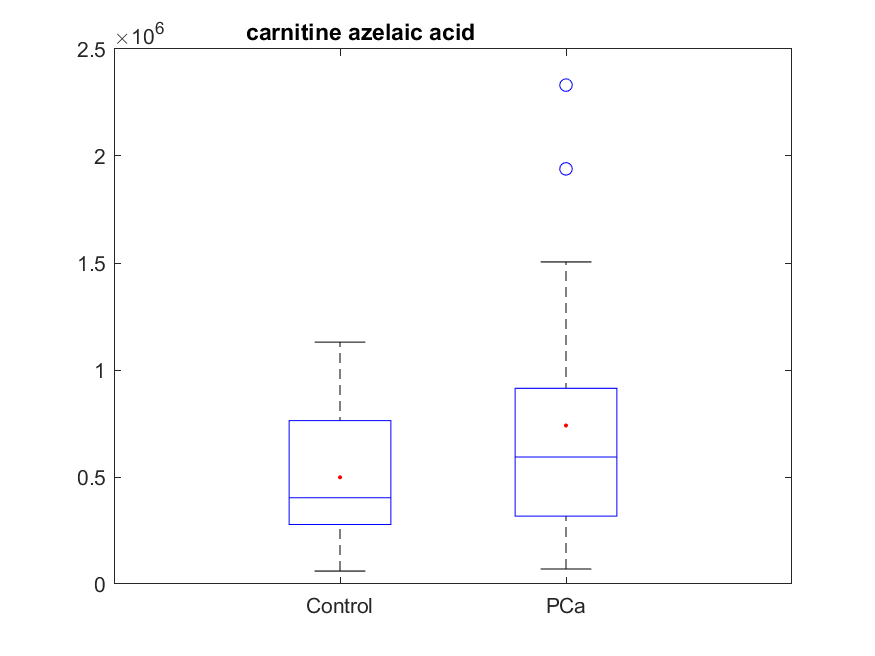


**Figure S18.** Box plot for compound 13 ESI+ (Carnitine azelaic acid)


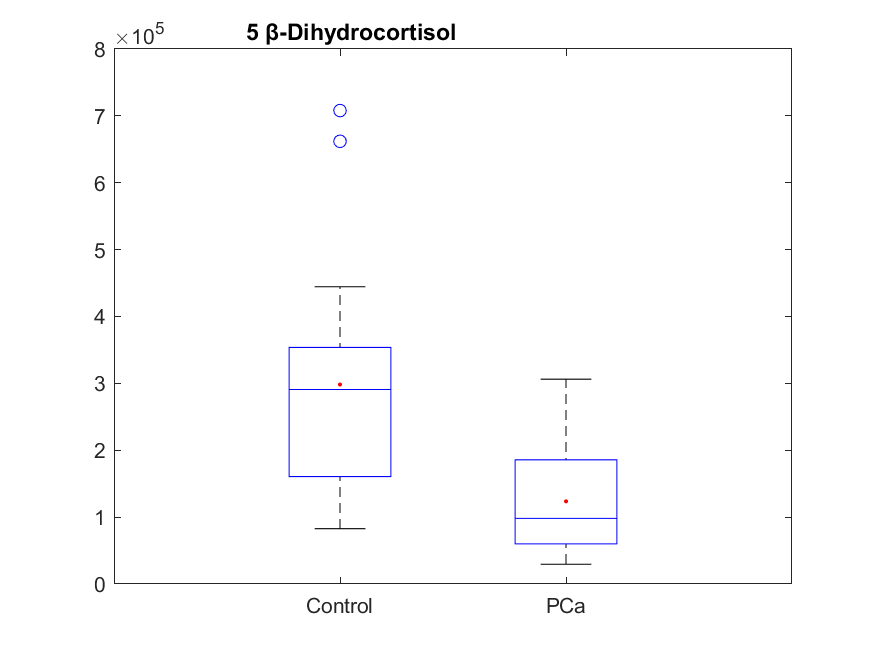


**Figure S19.** Box plot for compound 14 ESI+ (Dihydrocortisol)


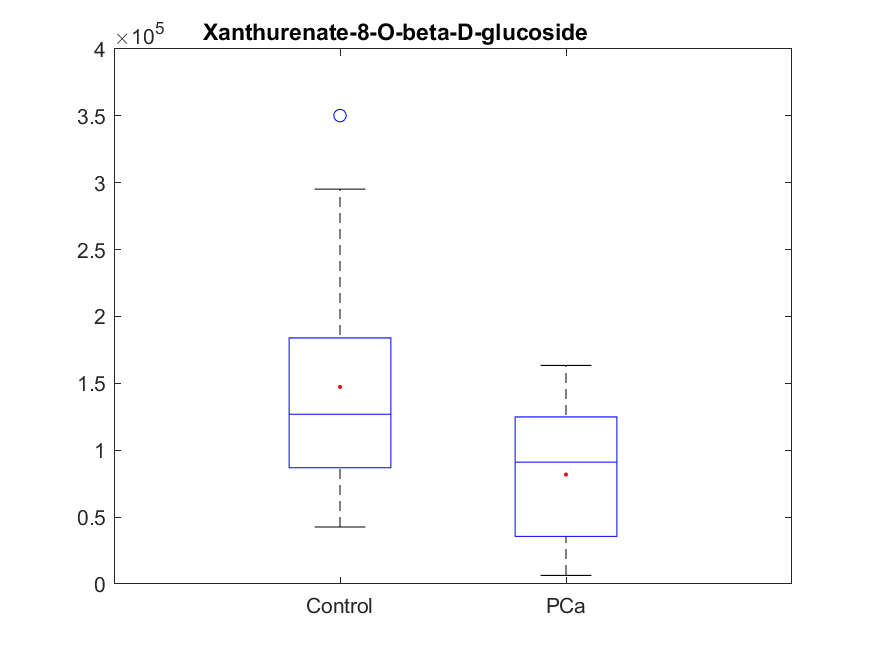


**Figure S20.** Box plot for compound 15 ESI+ (Xanthurenate-8-O-beta-D-glucoside)


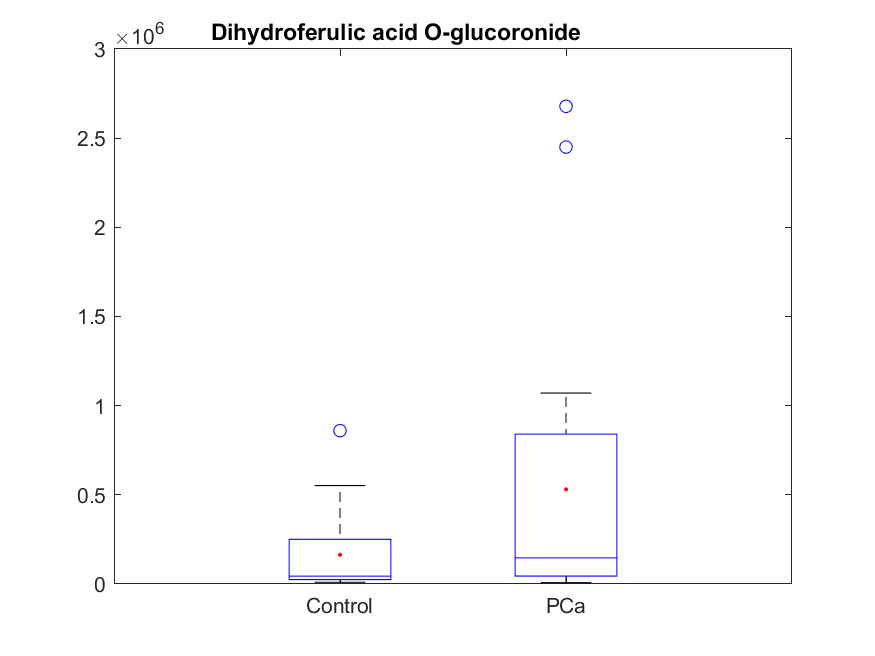


**Figure S21.** Box plot for compound 16-17 ESI+ (dihydro(iso)ferulic acid glucuronide)


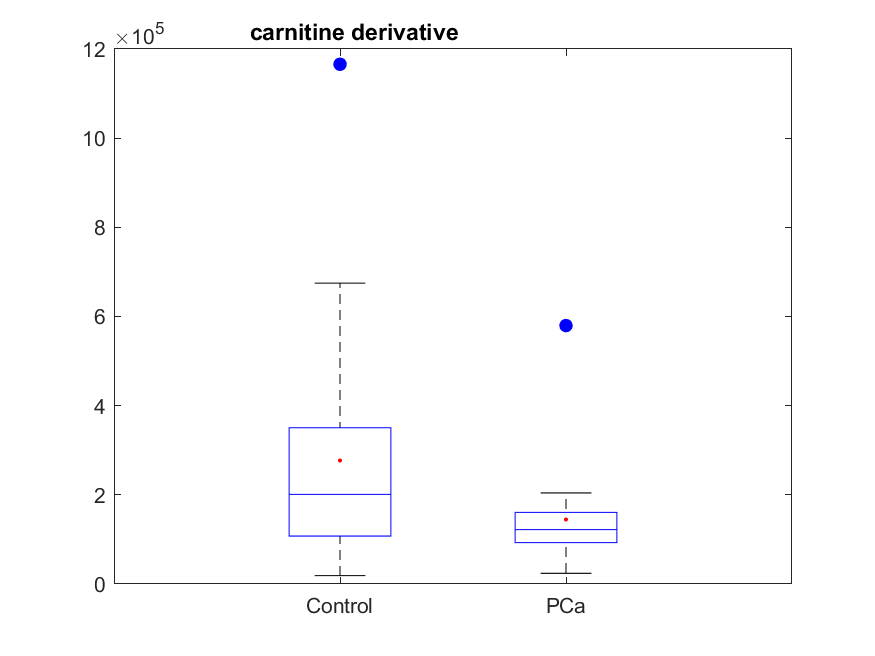


**Figure S22.** Box plot for compound 18 ESI+ (Carnitine derivative)


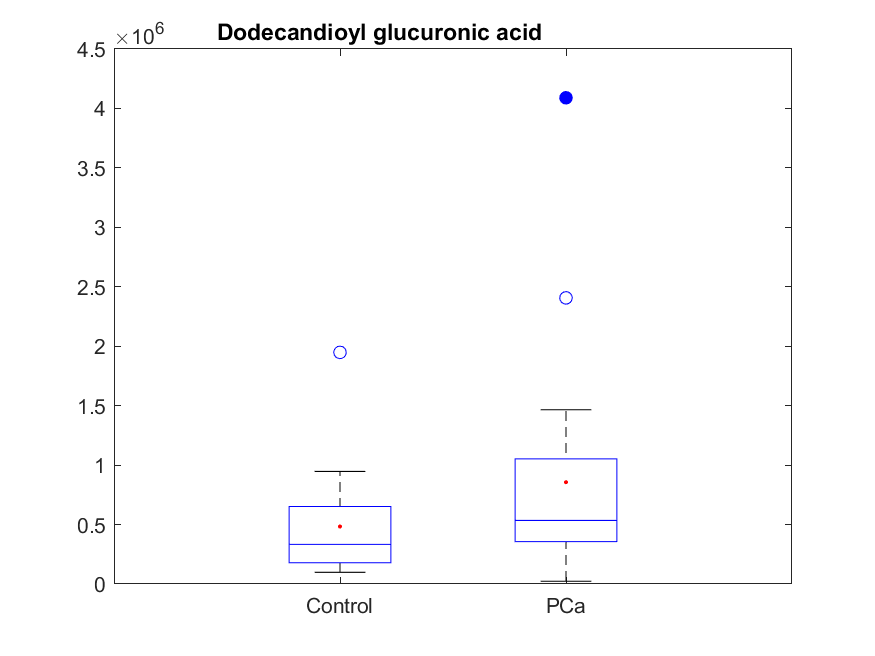


**Figure S23.** Box plot for compound 19 ESI+ (Dodecanedioyl glucuronic acid)


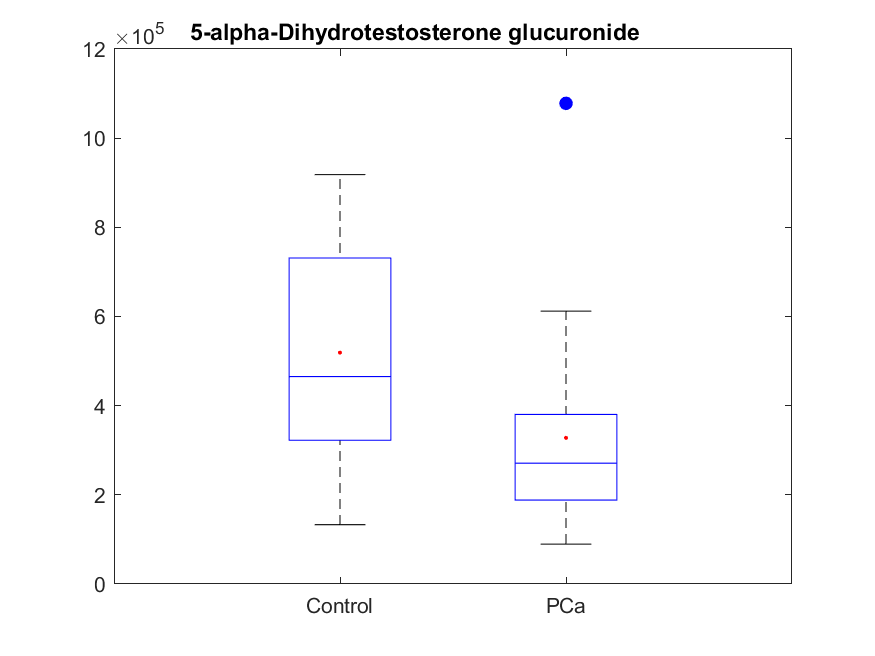


**Figure S24.** Box plot for compound 20 ESI+ (5-alpha-Dihydrotestosterone glucuronide)


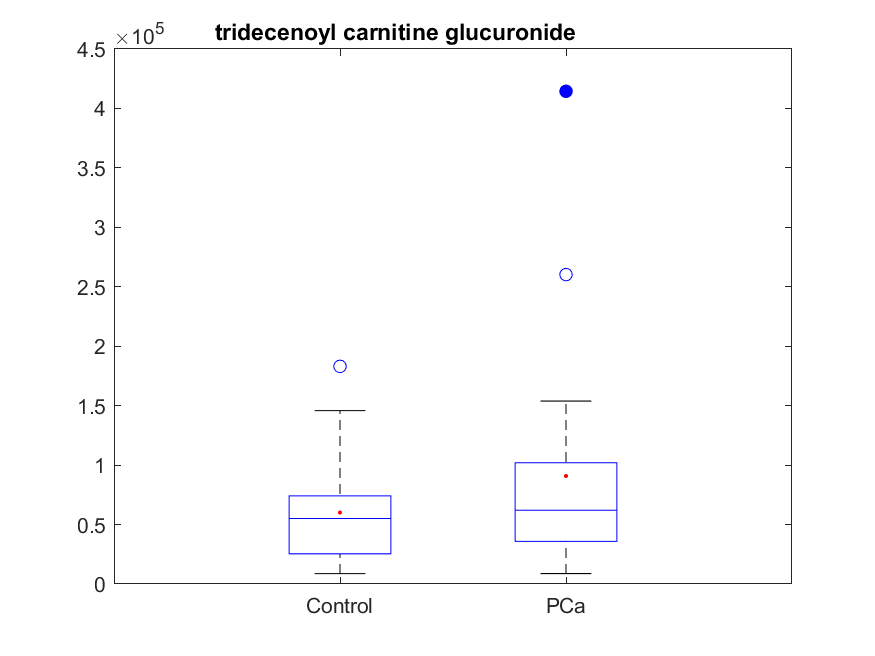


**Figure S25.** Box plot for compound 21 ESI+ (tridecenoyl carnitine glucuronide)


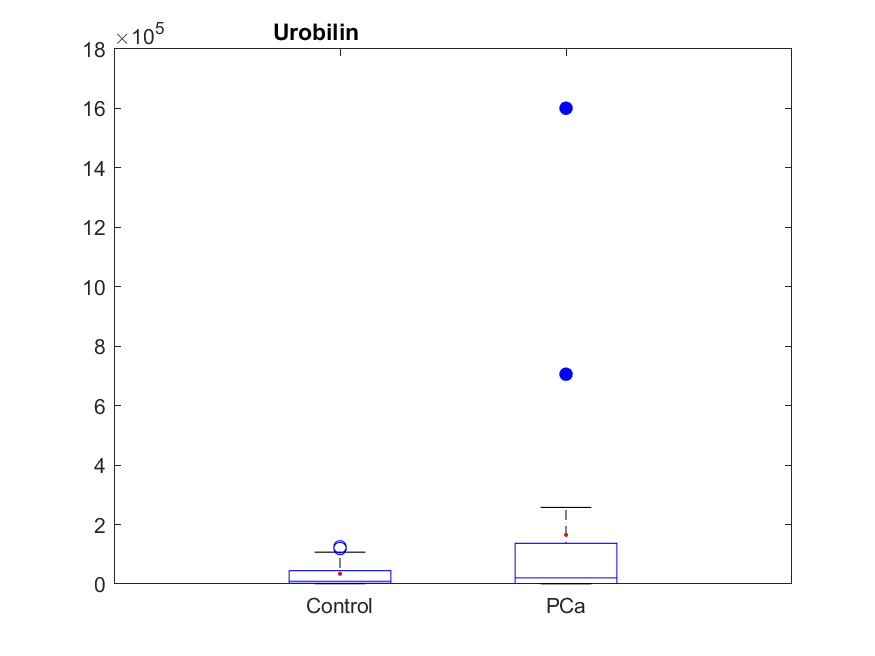


**Figure S26.** Box plot for compound 22 ESI+ (Urobilin)

**ESI-**

**
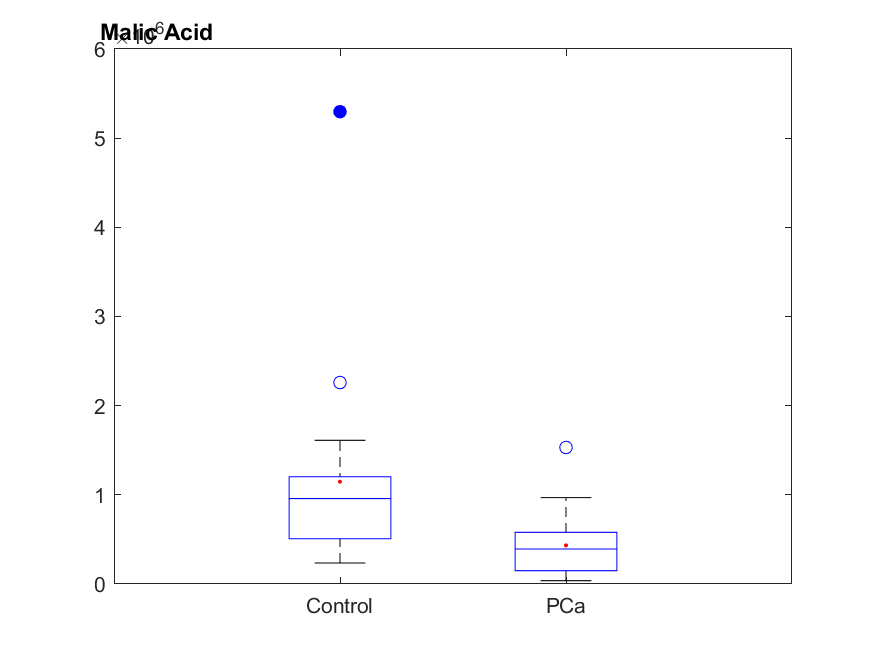
**

**Figure S27.** Box plot for compound 1 ESI- (Malic Acid)


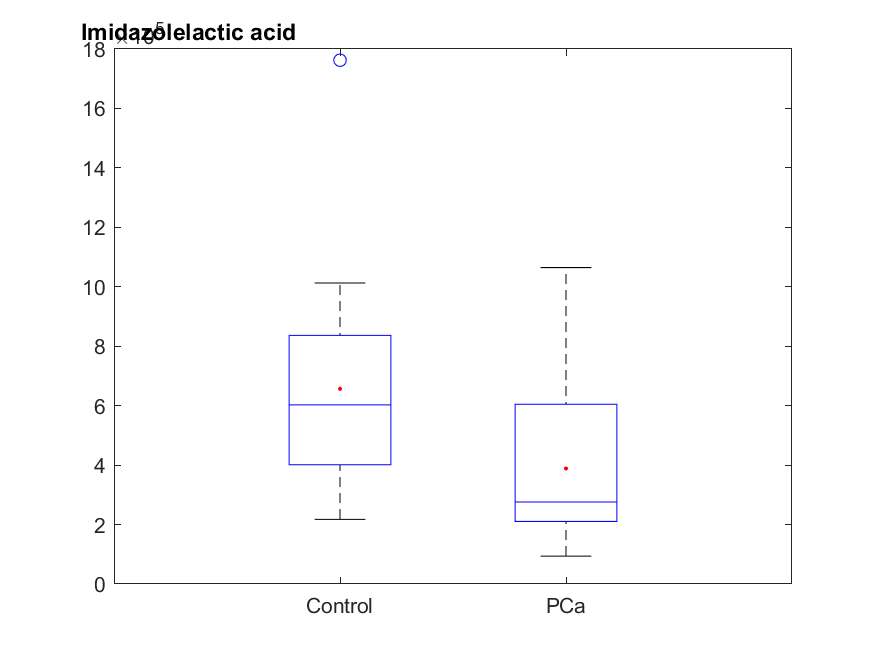


**Figure S28.** Box plot for compound 2 ESI- (Imidazolelactic acid)


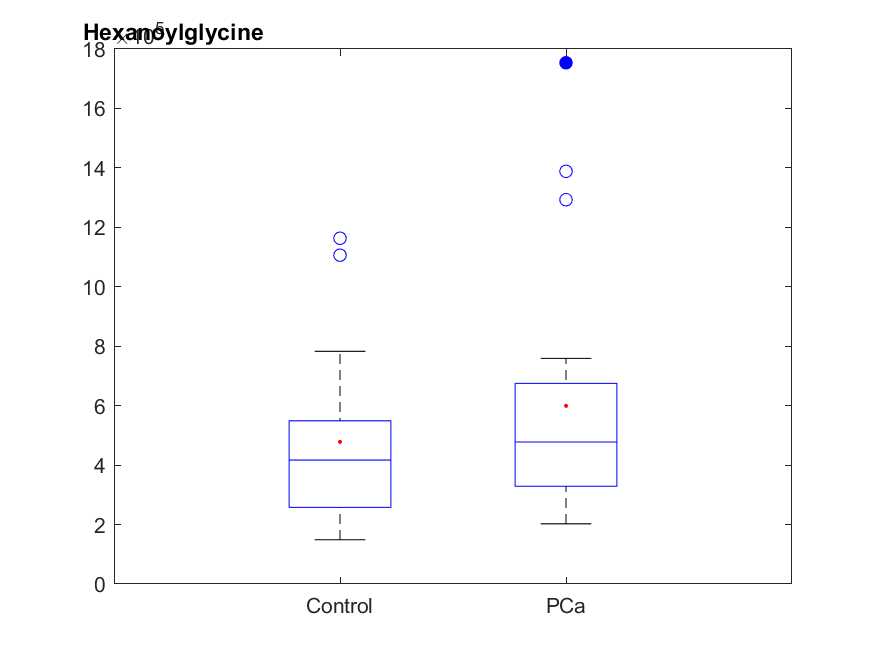


**Figure S29.** Box plot for compound 3 ESI- (Hexanoylglycine)


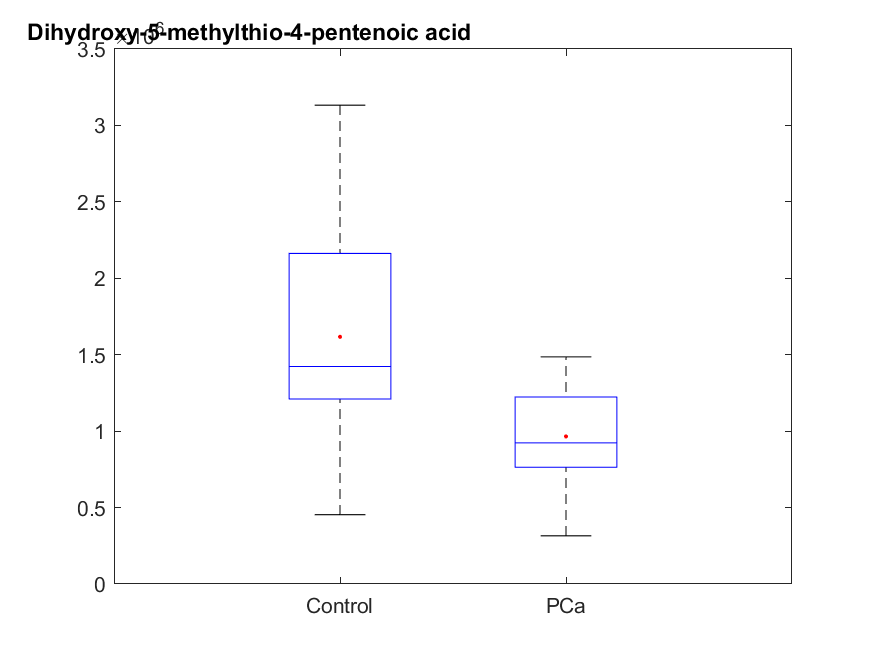


**Figure S30.** Box plot for compound 4 ESI- (Dihydroxy-5-methylthio-4-pentenoic acid (DMTPA))


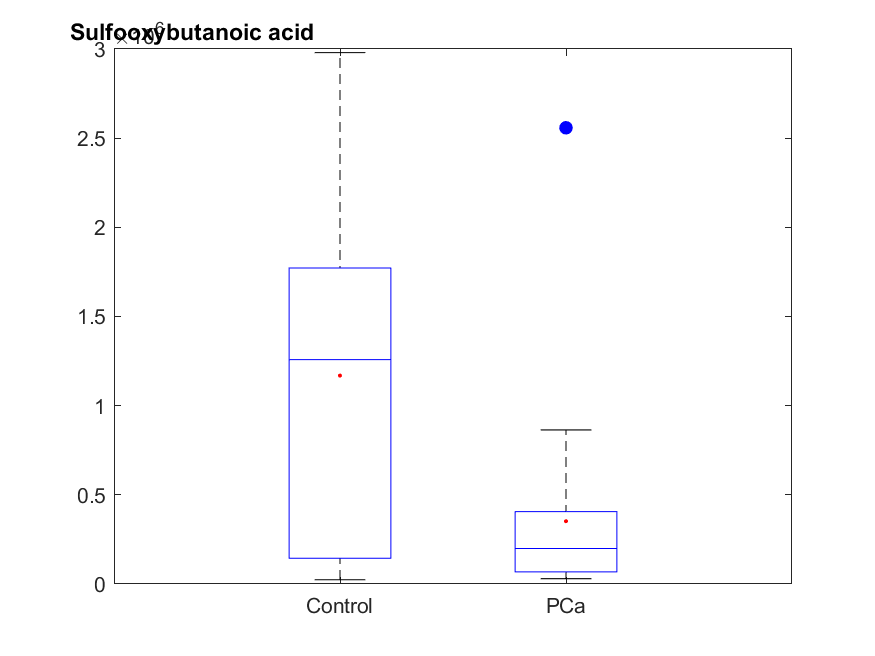


**Figure S31.** Box plot for compound 5 ESI- (Sulfooxybutanoic acid)


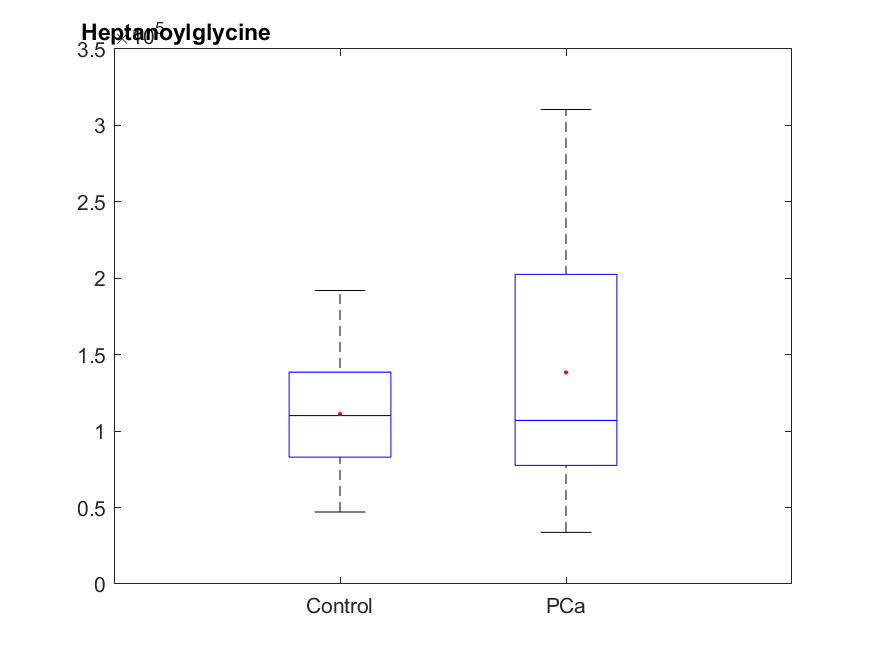


**Figure S32.** Box plot for compound 6 ESI- (Heptanoylglycine)


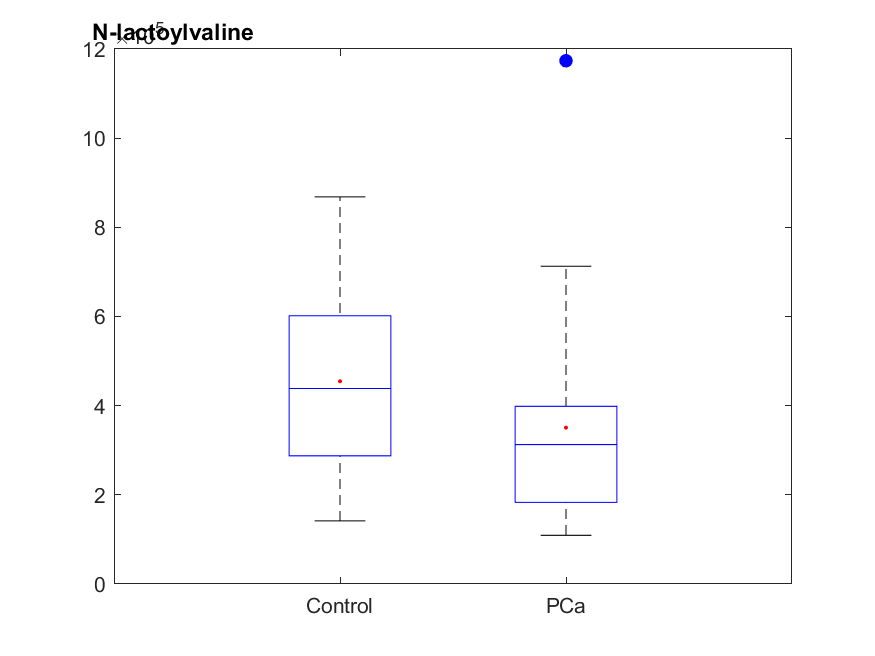


**Figure S33.** Box plot for compound 7 ESI- (N-lactoylvaline)


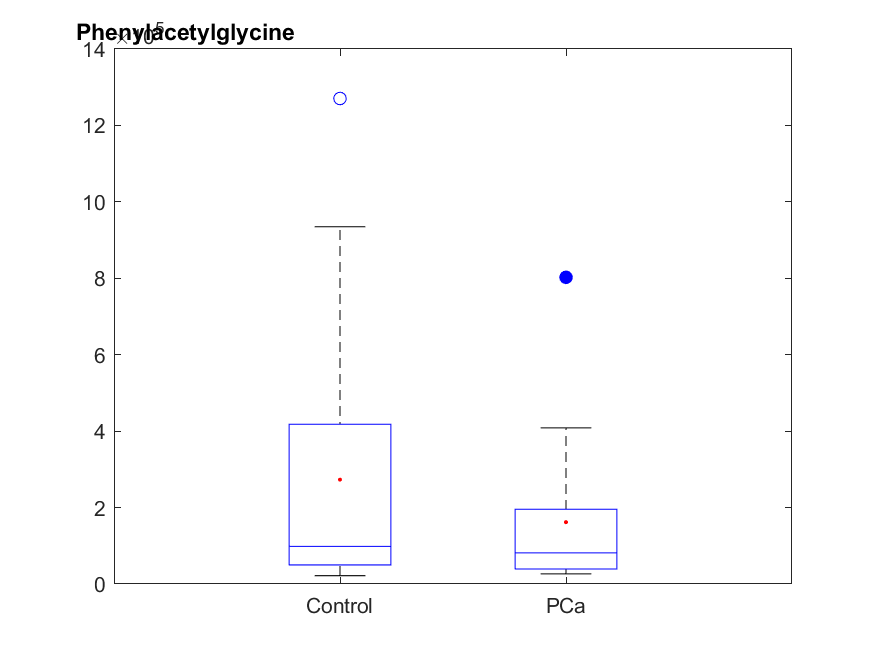


**Figure S34.** Box plot for compound 8 ESI- (Phenylacetylglycine)


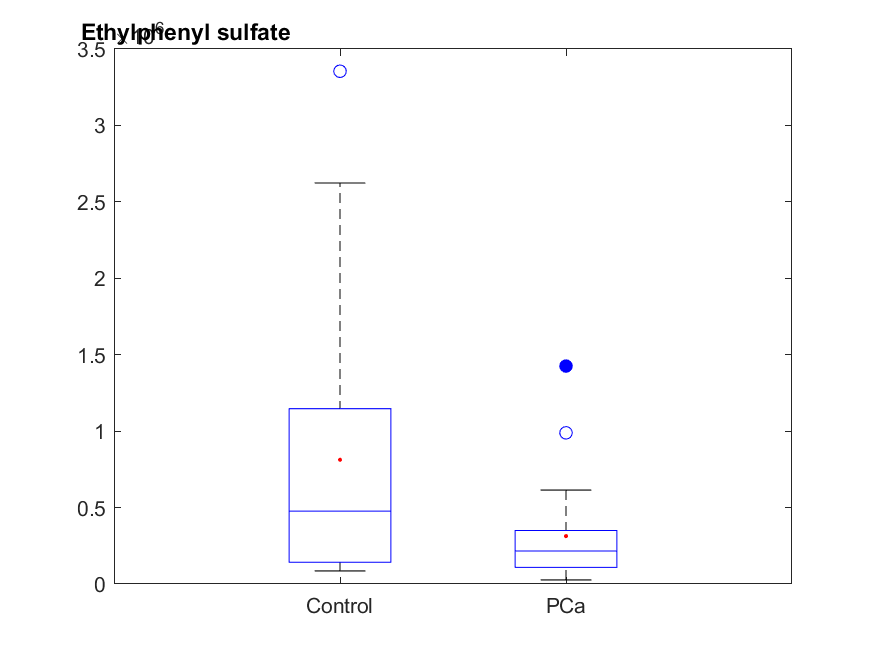


**Figure S35.** Box plot for compound 9 ESI- (Ethylphenyl sulfate)


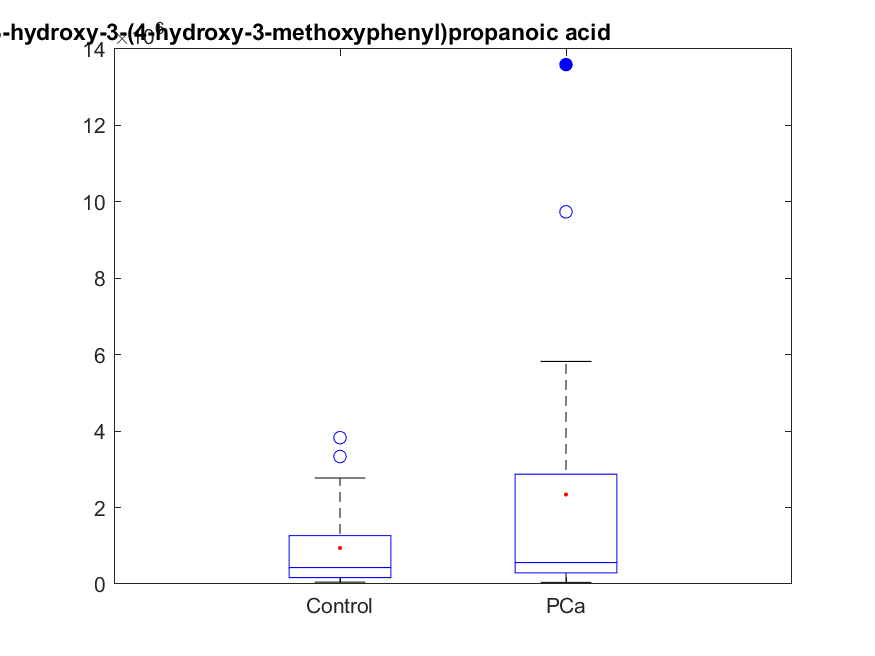


**Figure S36.** Box plot for compound 10 ESI- (3-hydroxy-3-(4-hydroxy-3-methoxyphenyl) propanoic acid)


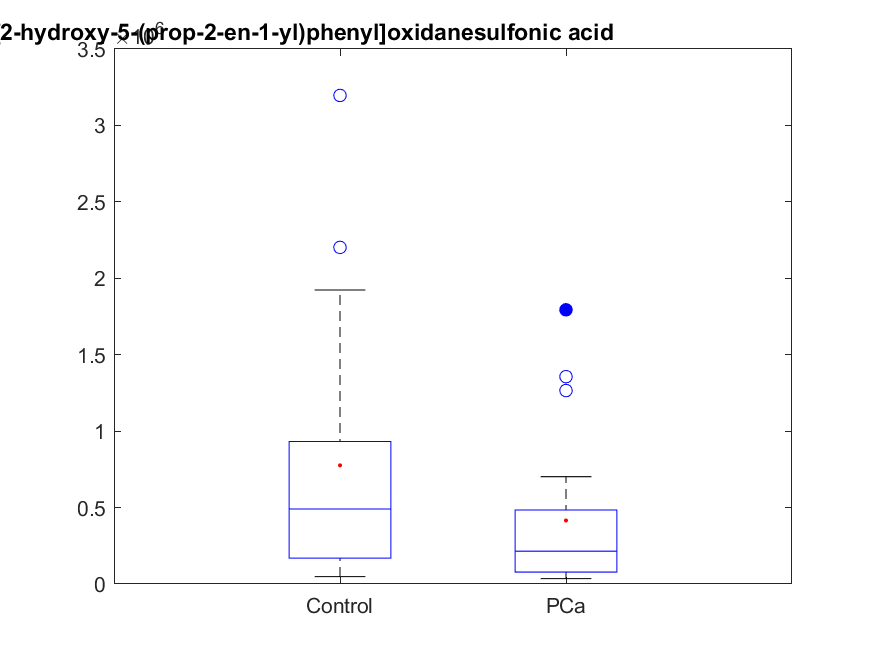


**Figure S37.** Box plot for compound 11 ESI- ([2-hydroxy-5-(prop-2-en-1-yl)phenyl]oxidanesulfonic acid)


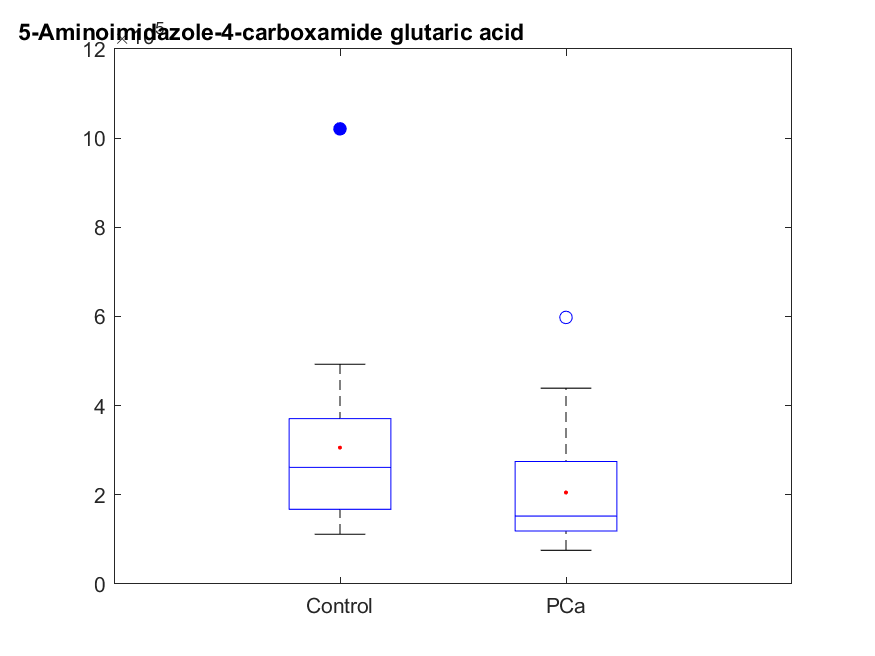


**Figure S38.** Box plot for compound 12 ESI- (5-Aminoimidazole-4-carboxamide glutaric acid)


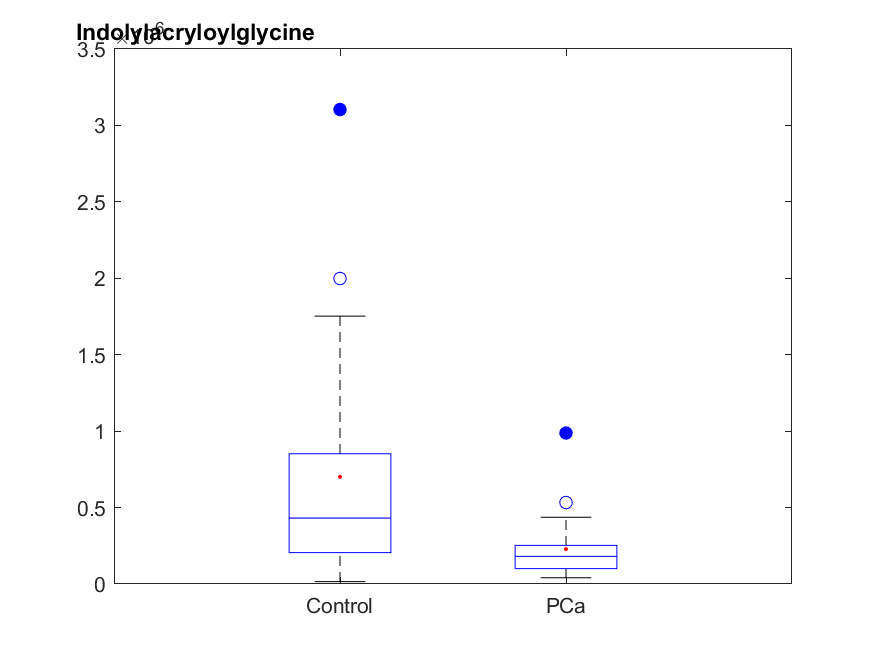


**Figure S39.** Box plot for compound 13 ESI- (Indolylacryloylglycine)


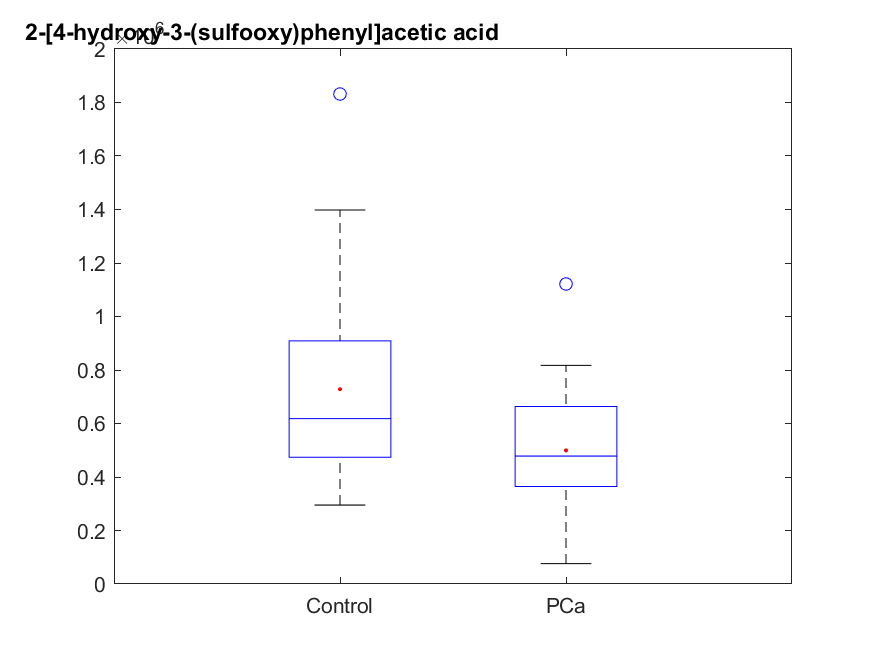


**Figure S40.** Box plot for compound 14 ESI- (2-[4-hydroxy-3-(sulfooxy) phenyl]acetic acid)


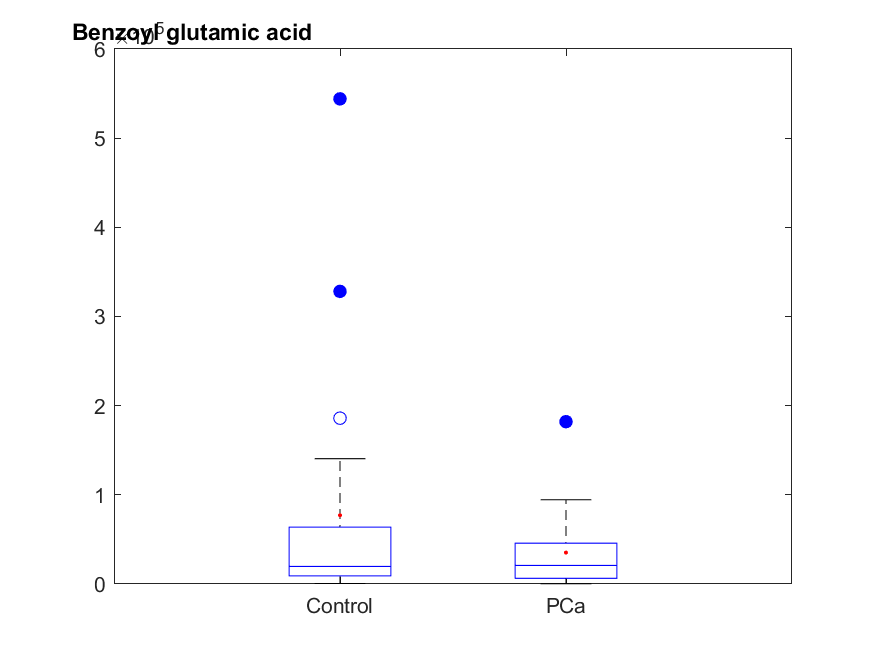


**Figure S41.** Box plot for compound 15 ESI- (Benzoyl glutamic acid)


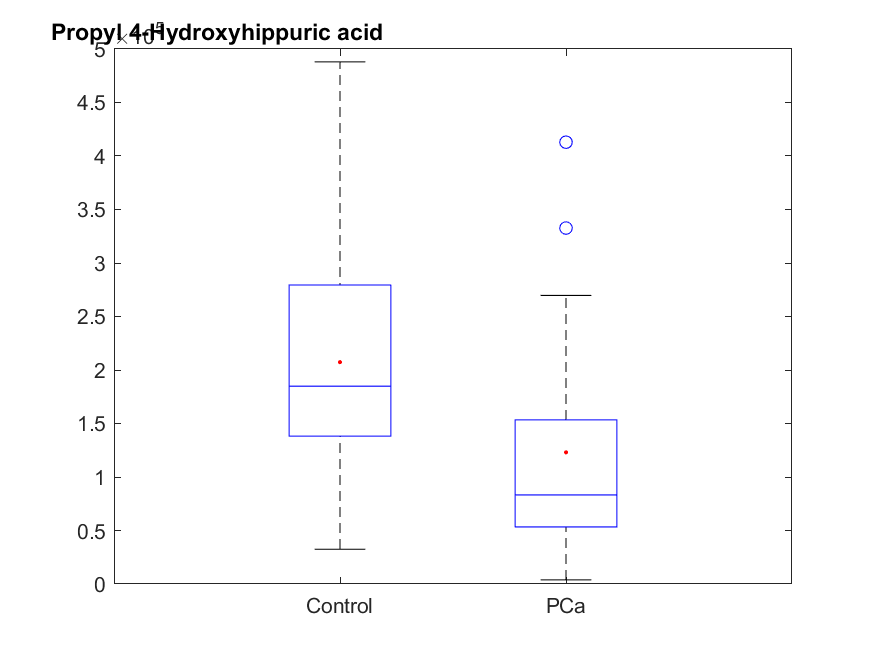


**Figure S42.** Box plot for compound 16 ESI- (Propyl hydroxyhippuric acid)


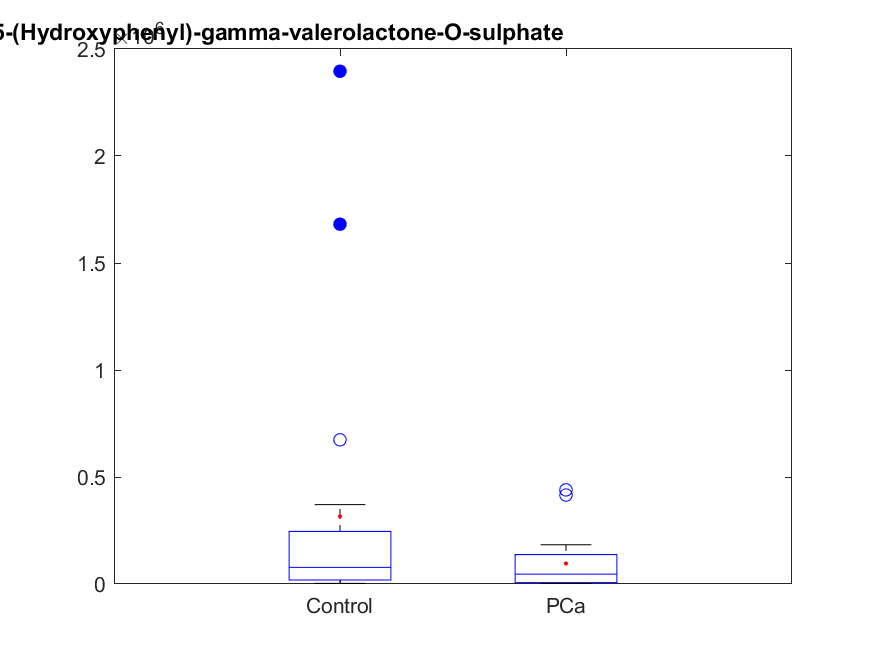


**Figure S43.** Box plot for compound 17 ESI- (5-(Hydroxyphenyl)-gamma-valerolactone-O-sulphate)


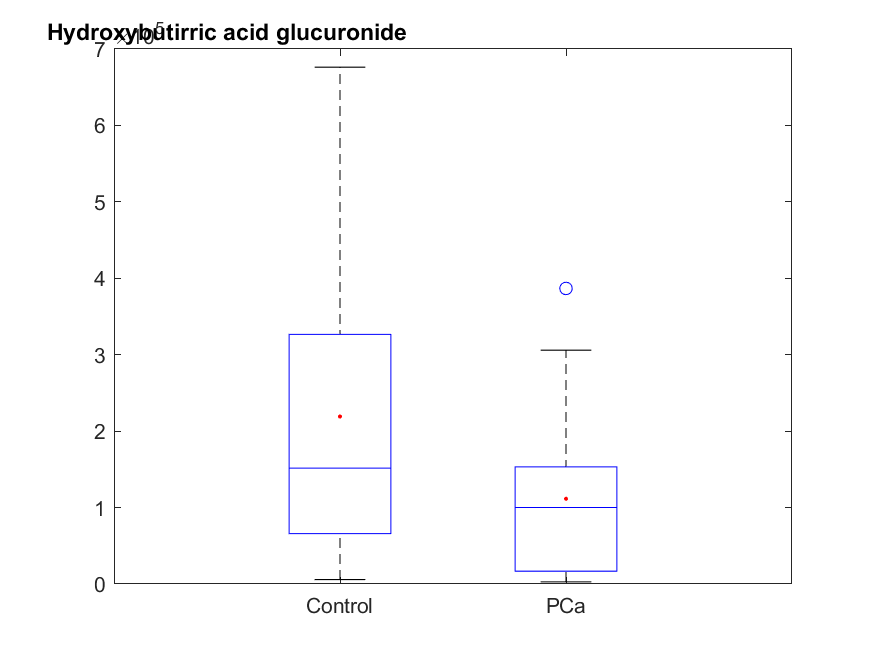


**Figure S44.** Box plot for compound 18 ESI- (Hydroxybutyric acid glucuronide)


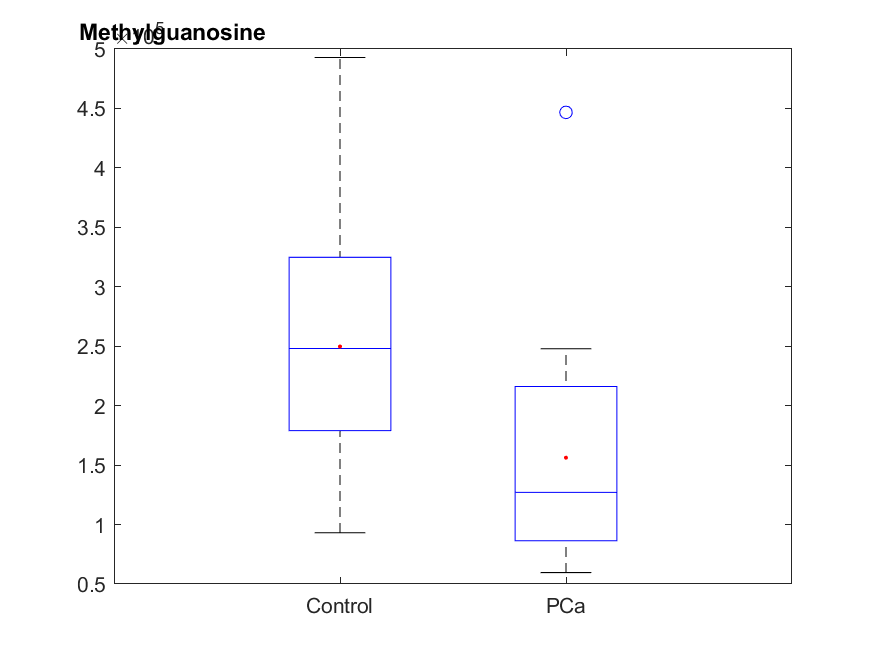


**Figure S45.** Box plot for compound 19 ESI- (Methylguanosine)


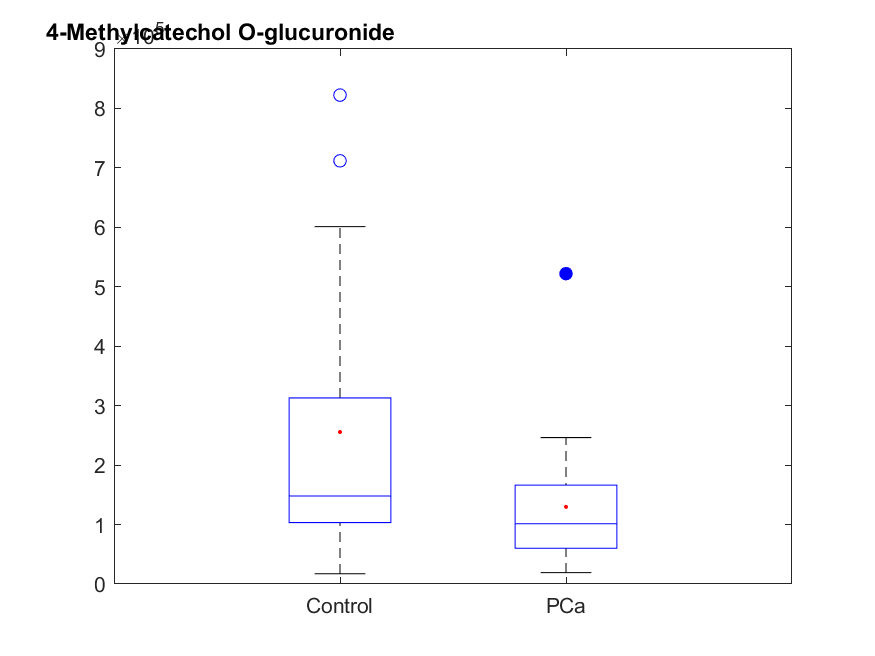


**Figure S46.** Box plot for compound 20 ESI- (4-Methylcatechol O-glucuronide)


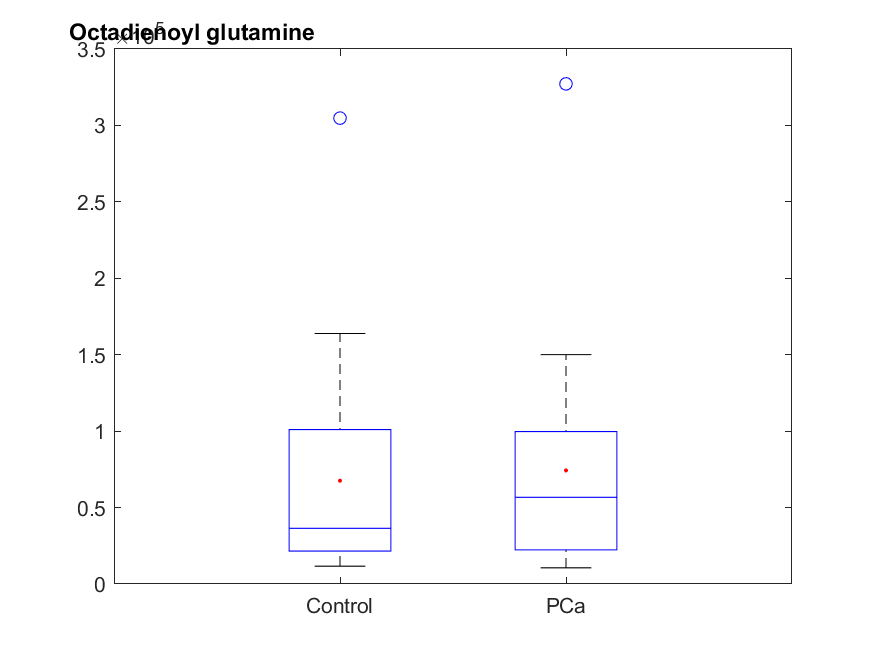


**Figure S47.** Box plot for compound 21 ESI- (Octenedioyl glutamine)


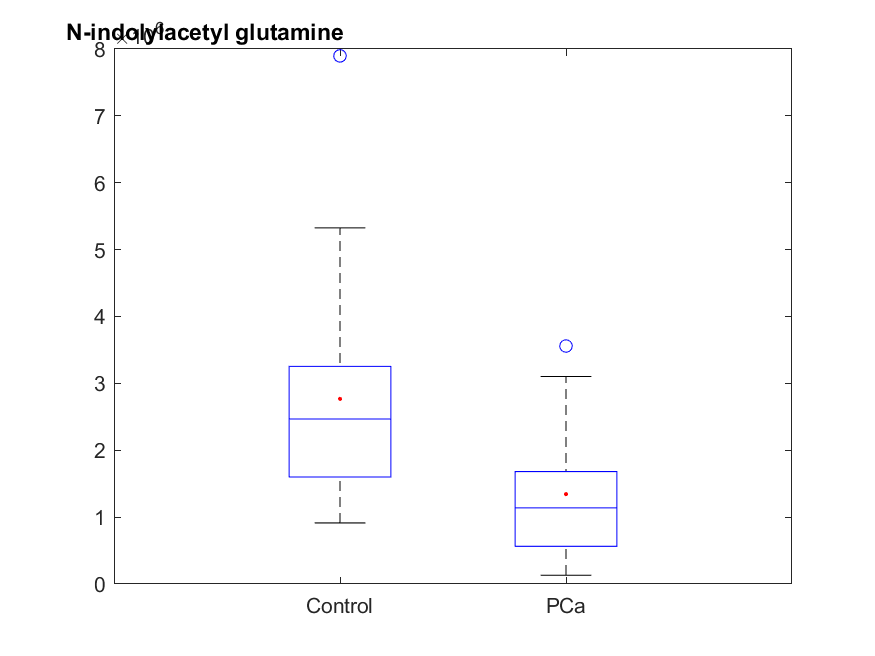


**Figure S48.** Box plot for compound 22 ESI- (N-(indol-3-acetyl) glutamine)


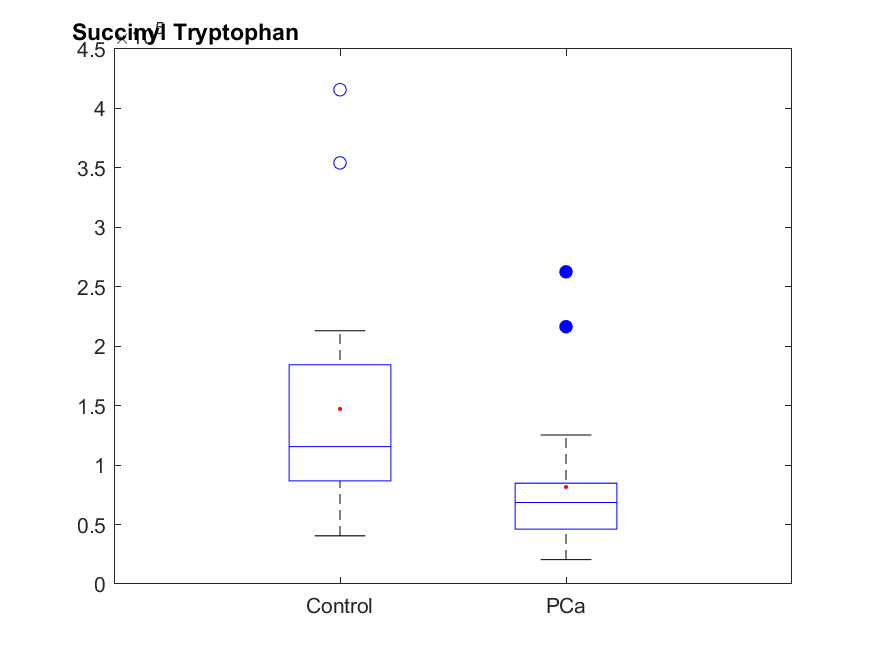


**Figure S49.** Box plot for compound 23 ESI- (Succinyl Tryptophan)


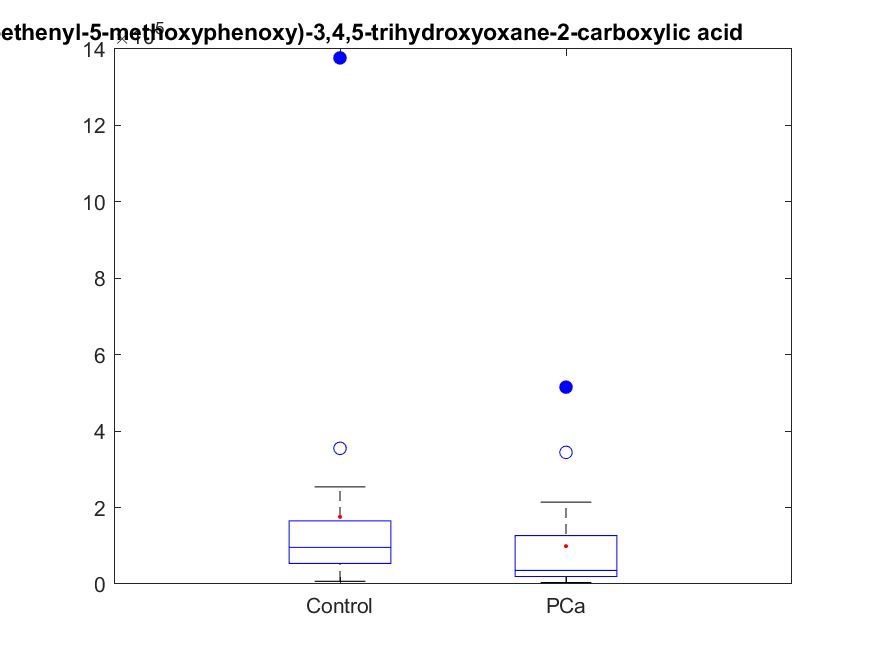


**Figure S50.** Box plot for compound 24 ESI- (2-Methoxy-4-vinylphenol glucuronide)


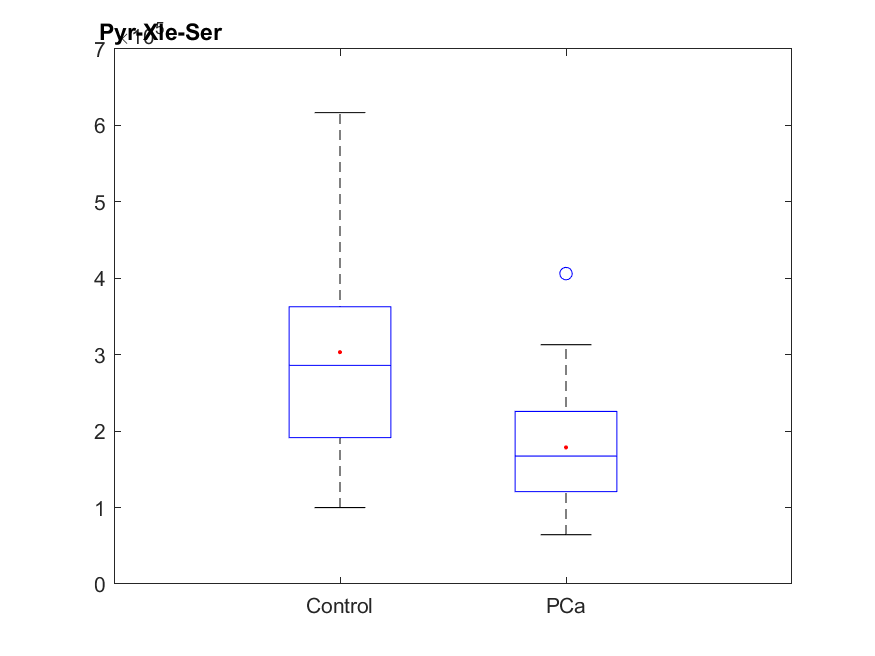


**Figure S51.** Box plot for compound 25 ESI- (Pyr-Xle-Ser)


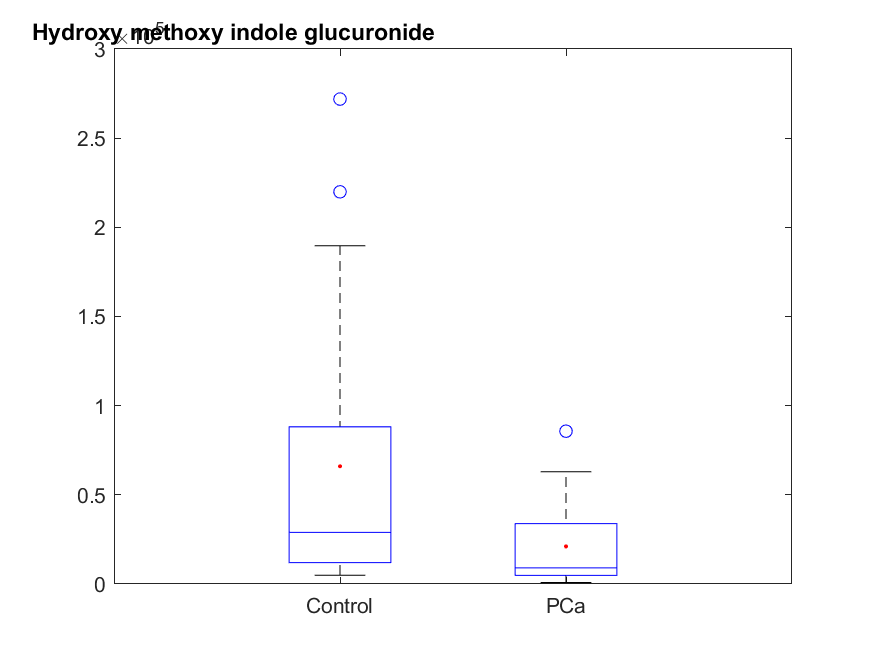


**Figure S52.** Box plot for compound 26 ESI- (Hydroxy methoxy indole glucuronide)


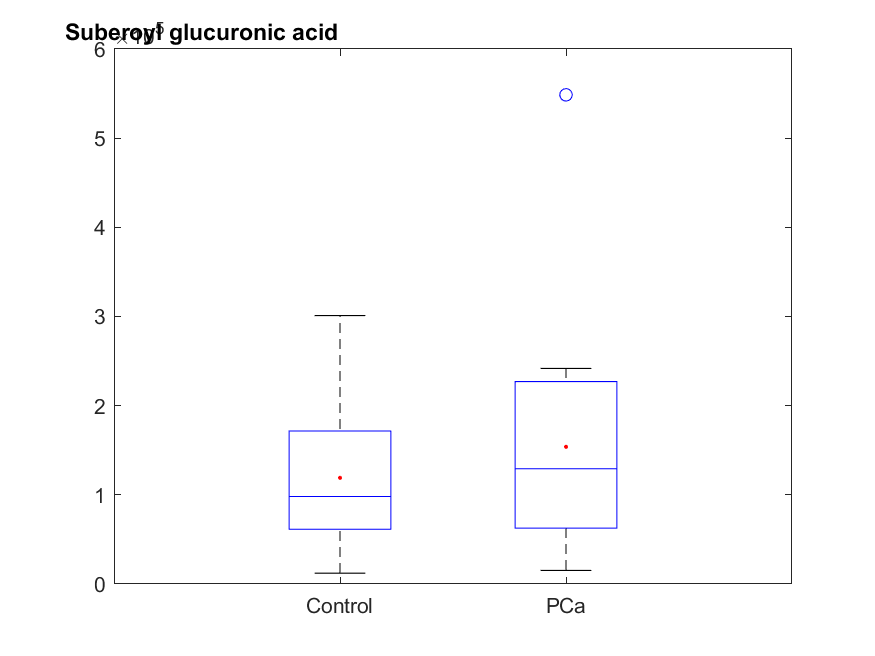


**Figure S53.** Box plot for compound 27 ESI- (Suberoyl glucuronic acid)


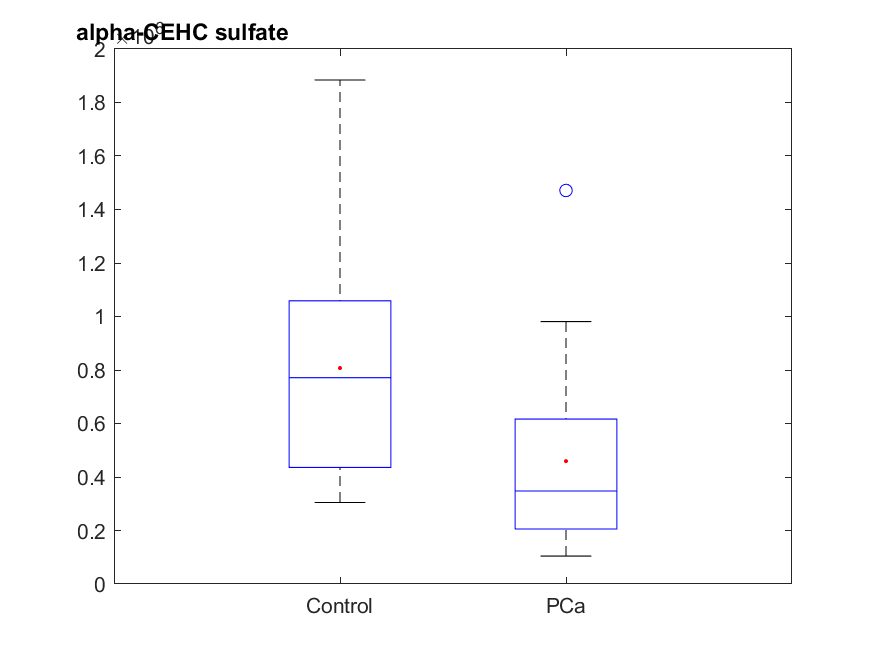


**Figure S54.** Box plot for compound 28 ESI- (alpha-CEHC sulfate)


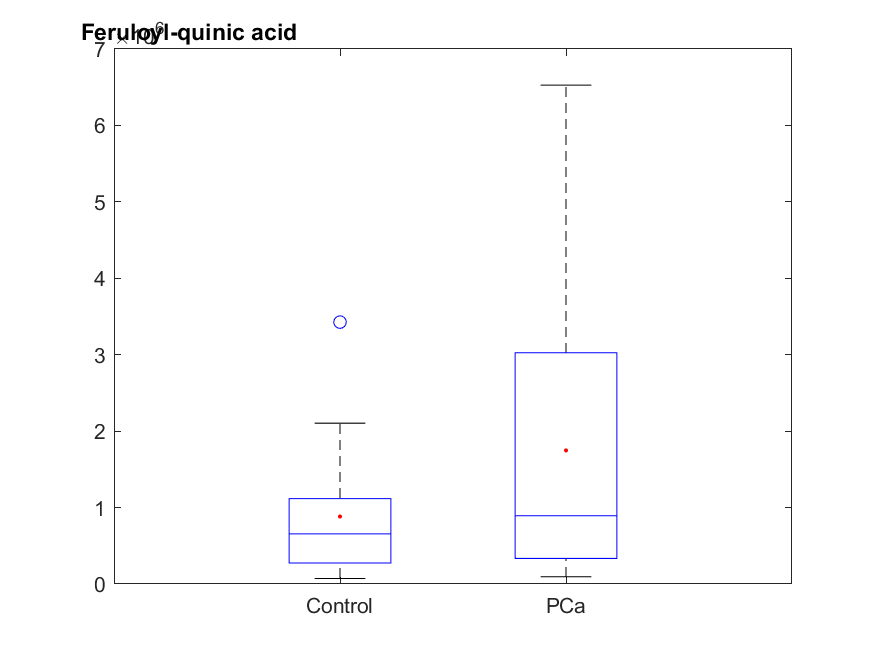


**Figure S55.** Box plot for compound 29 ESI- (Feruloyl-quinic acid)


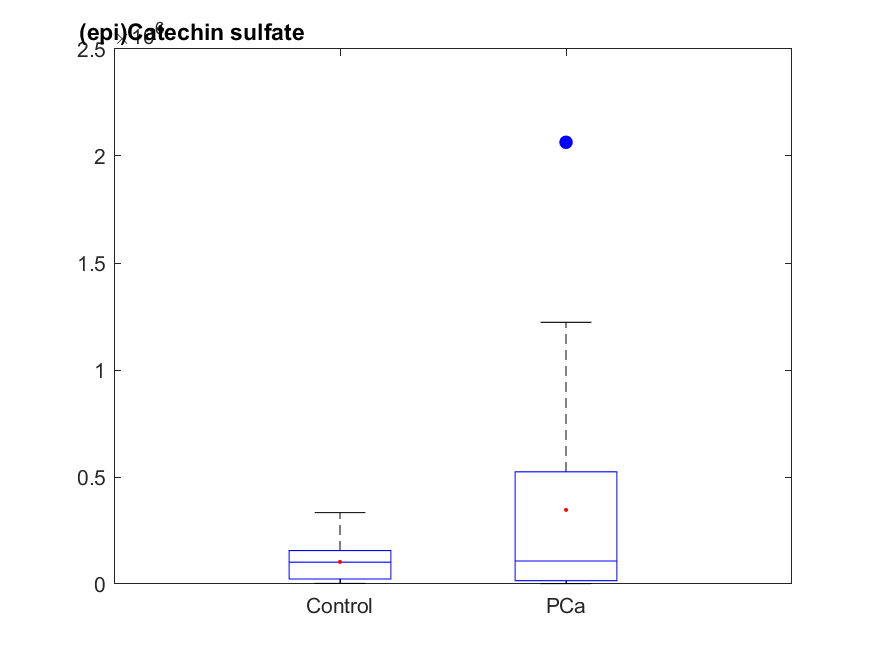


**Figure S56.** Box plot for compound 30 ESI- ((epi)catechin sulfate)


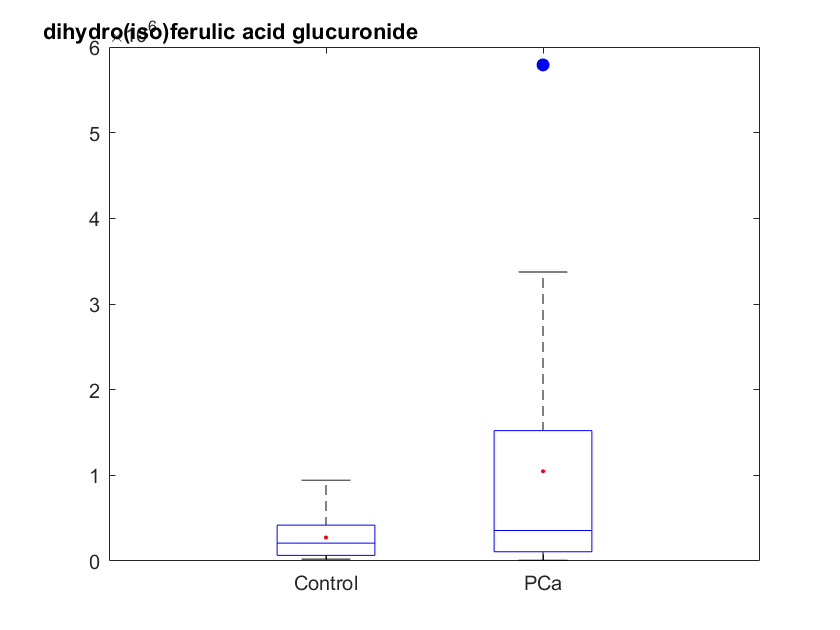


**Figure S57.** Box plot for compound 31 ESI- (dihydro(iso)ferulic acid glucuronide)


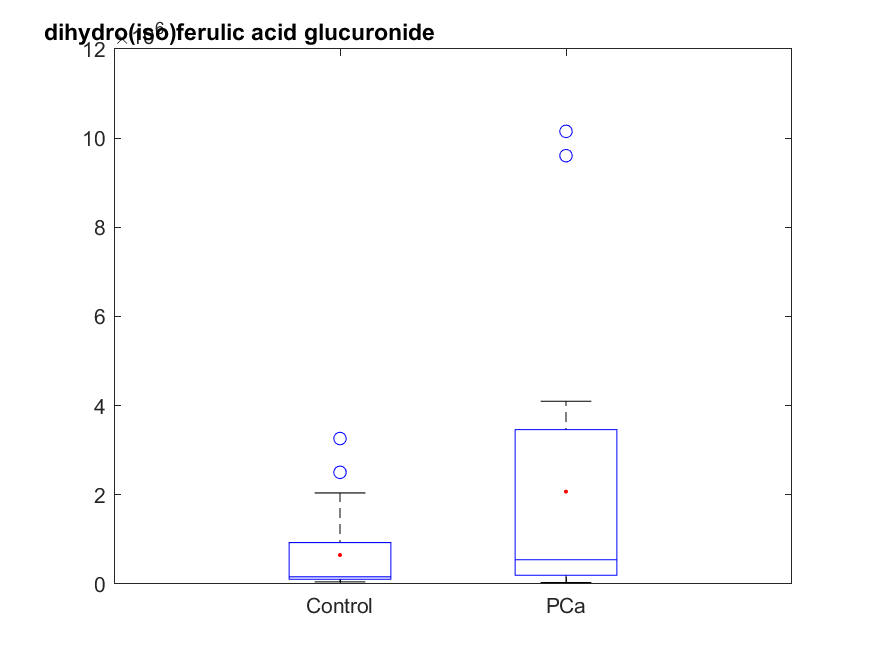


**Figure S58.** Box plot for compound 32 ESI- (dihydro(iso)ferulic acid glucuronide)


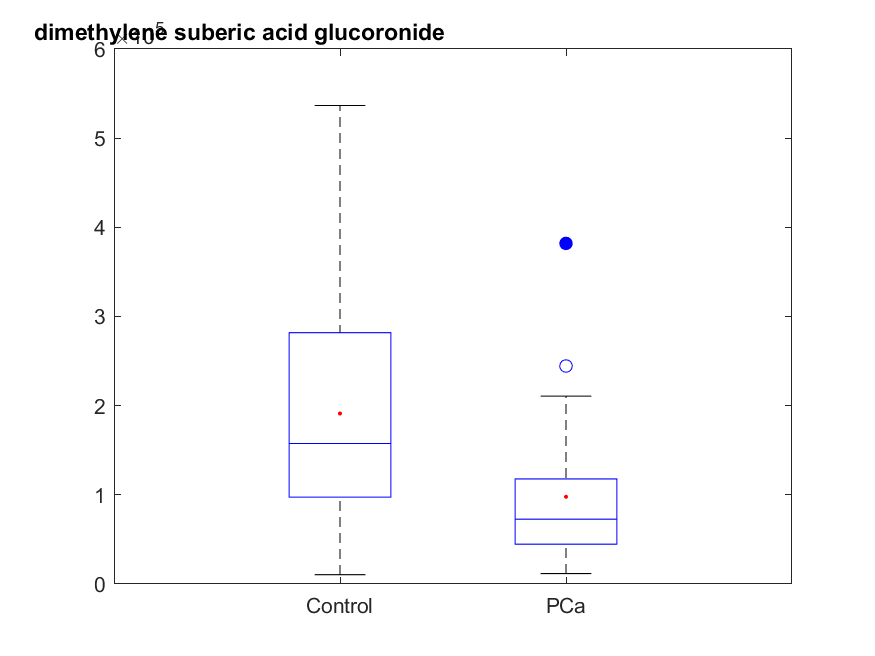


**Figure S59.** Box plot for compound 33 ESI- (dimethylene suberic acid glucoronide)


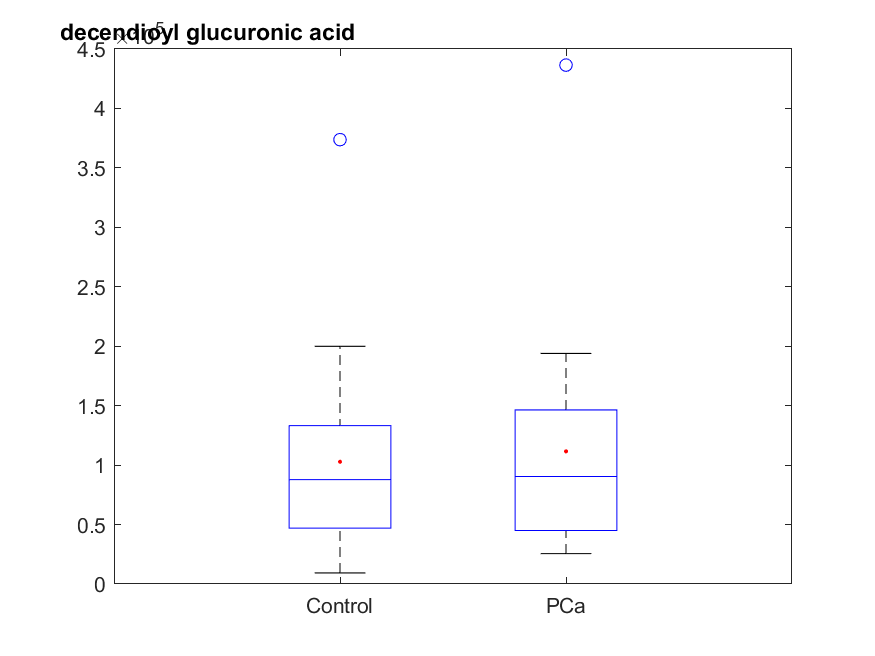


**Figure S60.** Box plot for compound 35 ESI- (decenedioyl glucuronic acid)


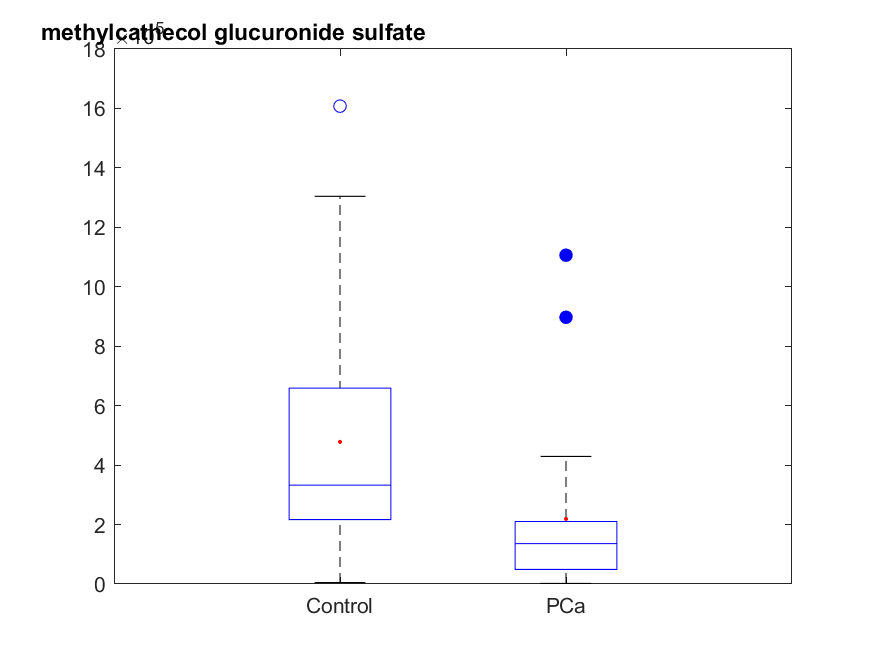


**Figure S61.** Box plot for compound 36 ESI- (methylcathecol glucuronide sulfate)


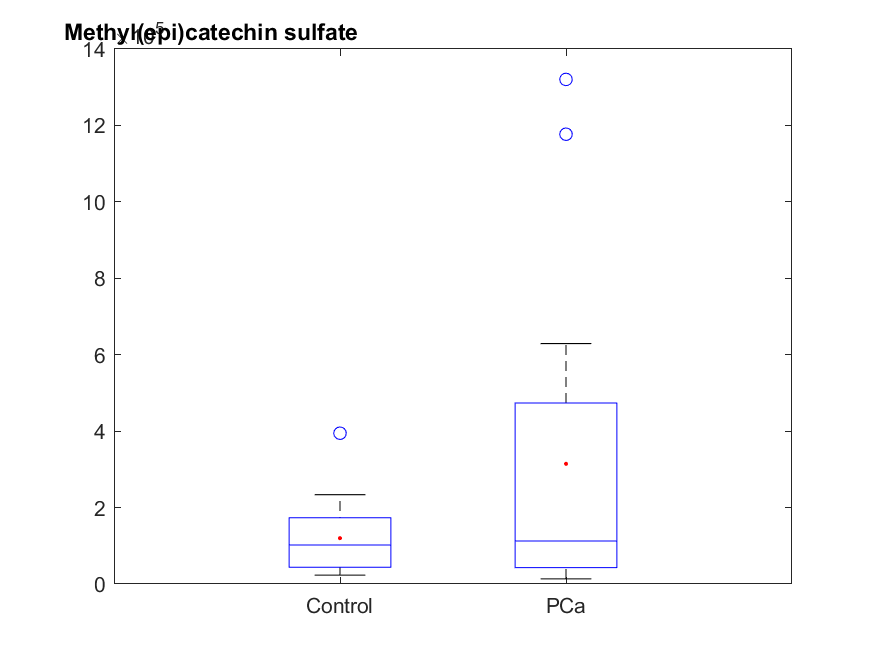


**Figure S62.** Box plot for compound 37 ESI- (Methyl(epi)catechin sulfate)


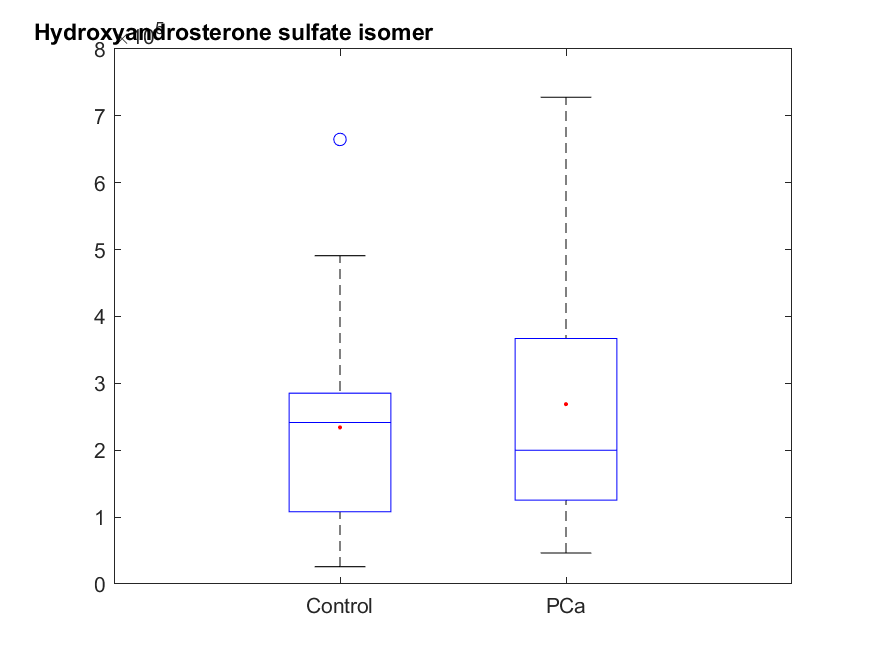


**Figure S63.** Box plot for compound 38 ESI- (Hydroxyandrosterone sulfate isomer)


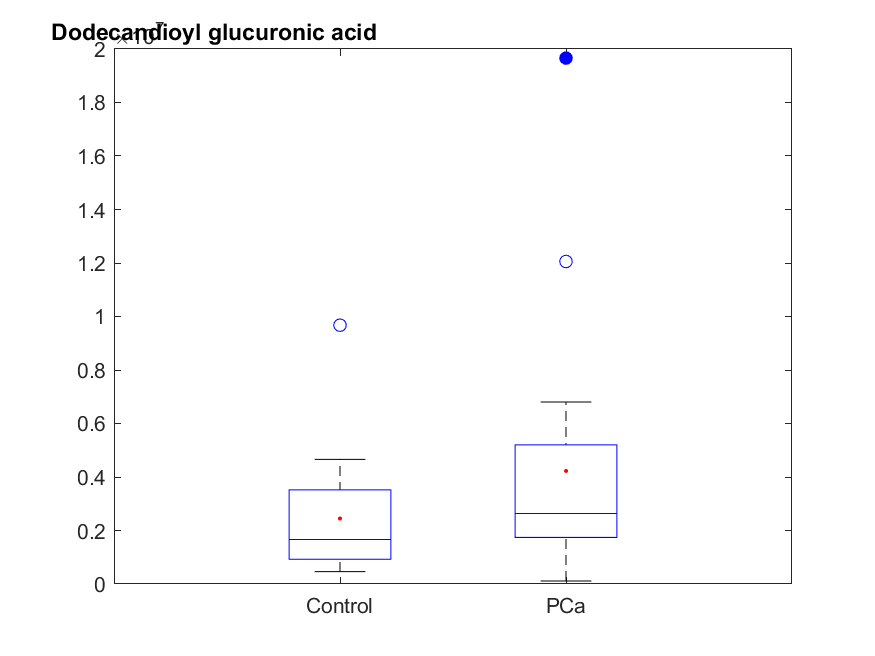


**Figure S64.** Box plot for compound 39 ESI- (Dodecanedioyl glucuronic acid)


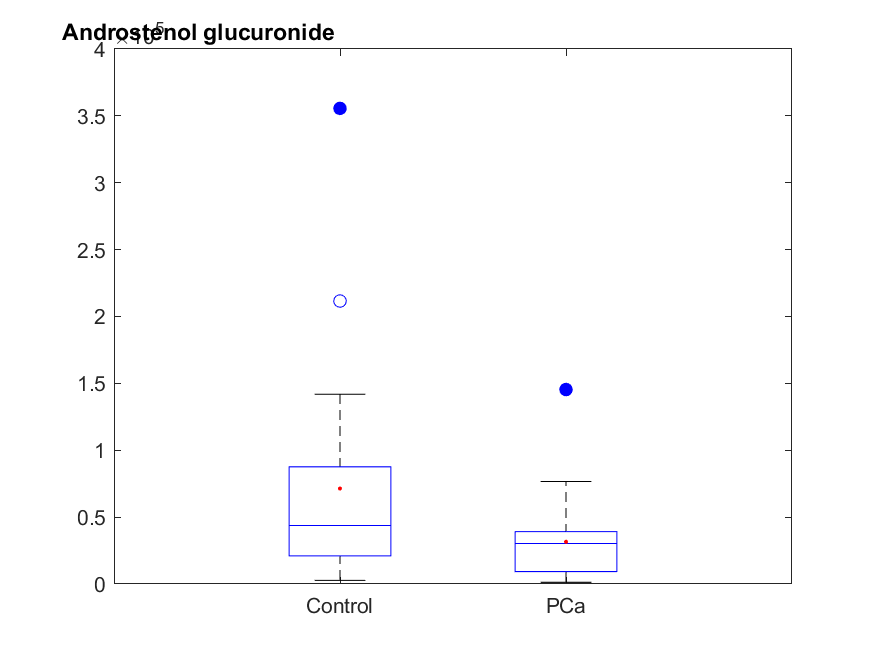


**Figure S65.** Box plot for compound 40 ESI- (Androstenol glucuronide)


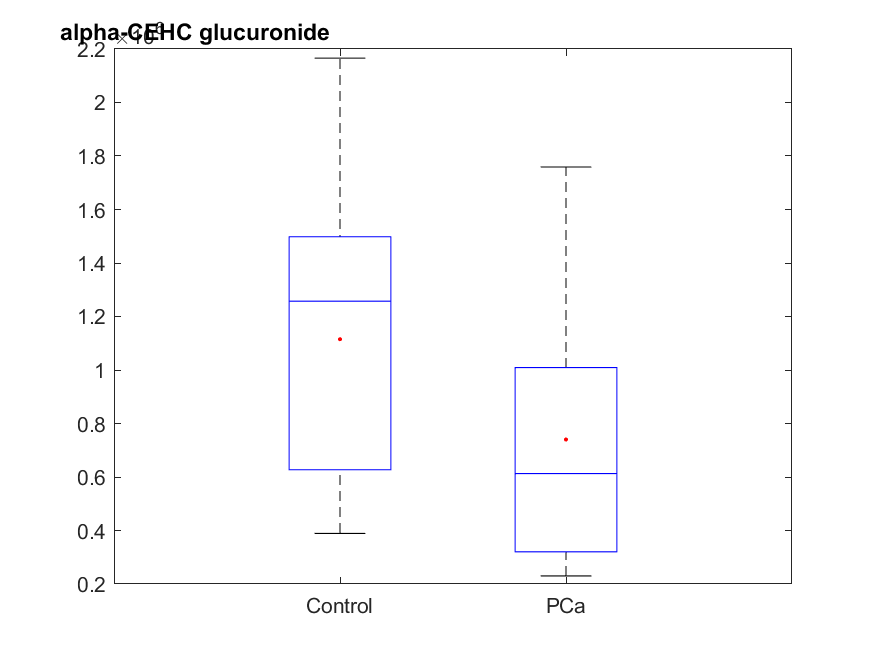


**Figure S66.** Box plot for compound 41 ESI- (alpha-CEHC glucuronide)


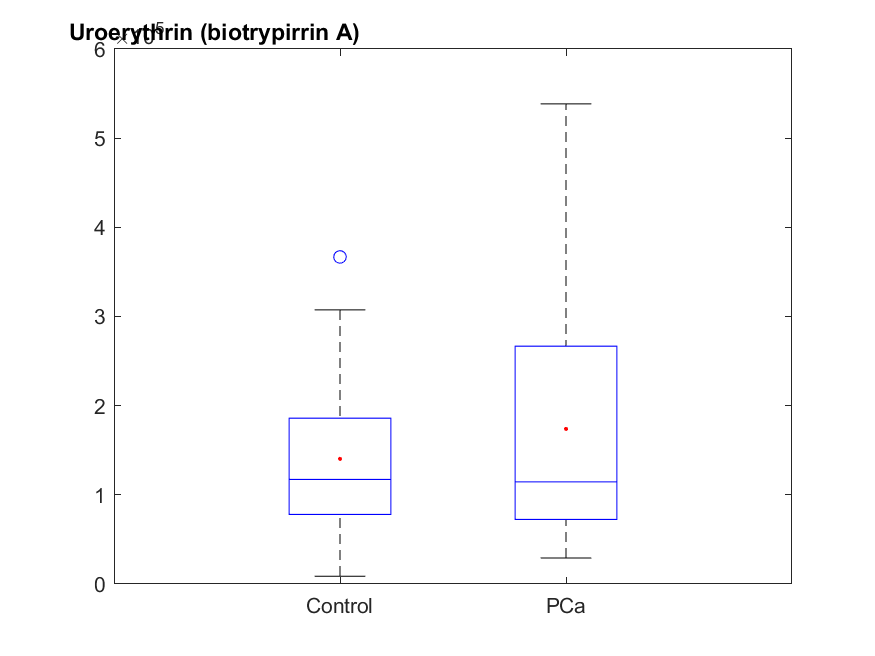


**Figure S67.** Box plot for compound 42 ESI- (Uroerythrin (biotrypirrin A))


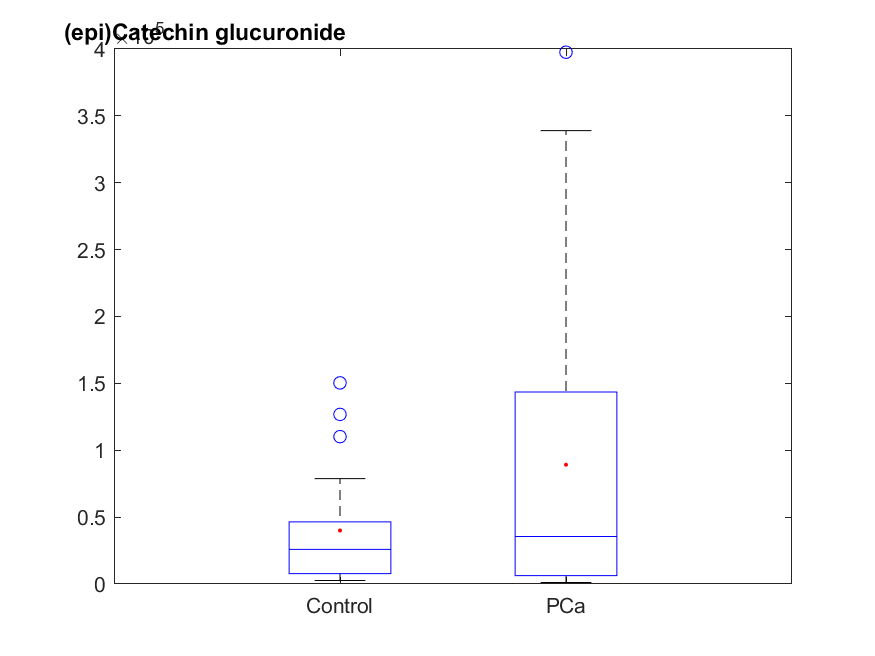


**Figure S68.** Box plot for compound 43 ESI- ((epi)Catechin glucuronide)


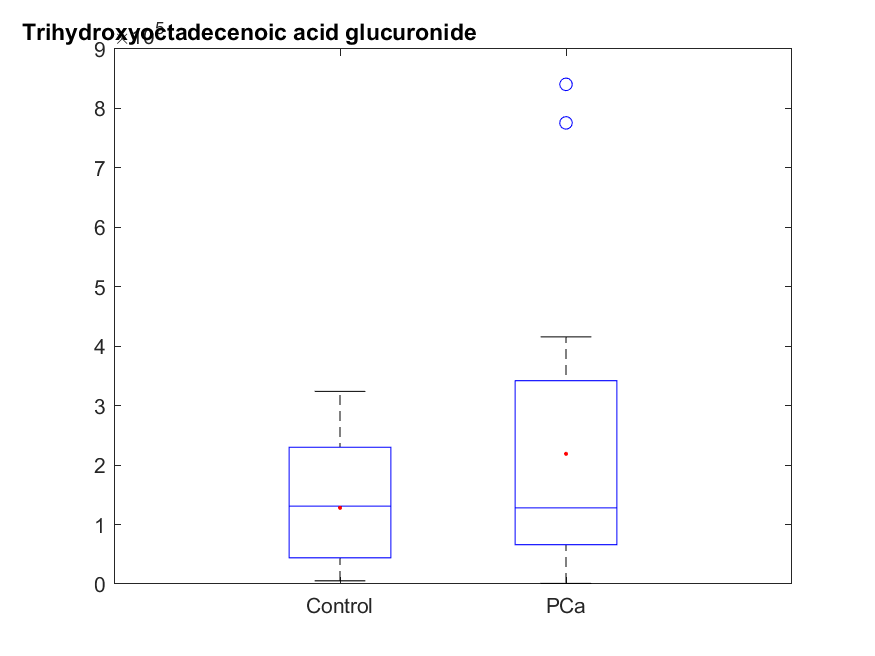


**Figure S69.** Box plot for compound 44 ESI- (Trihydroxyoctadecenoic acid glucuronide)


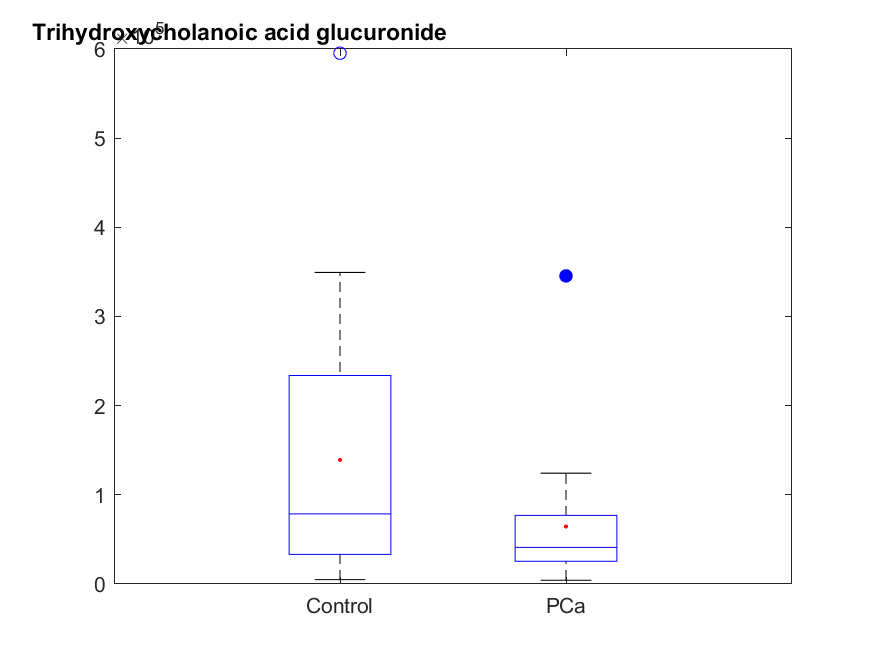


**Figure S70.** Box plot for compound 46 ESI- (Trihydroxycholanoic acid glucuronide)


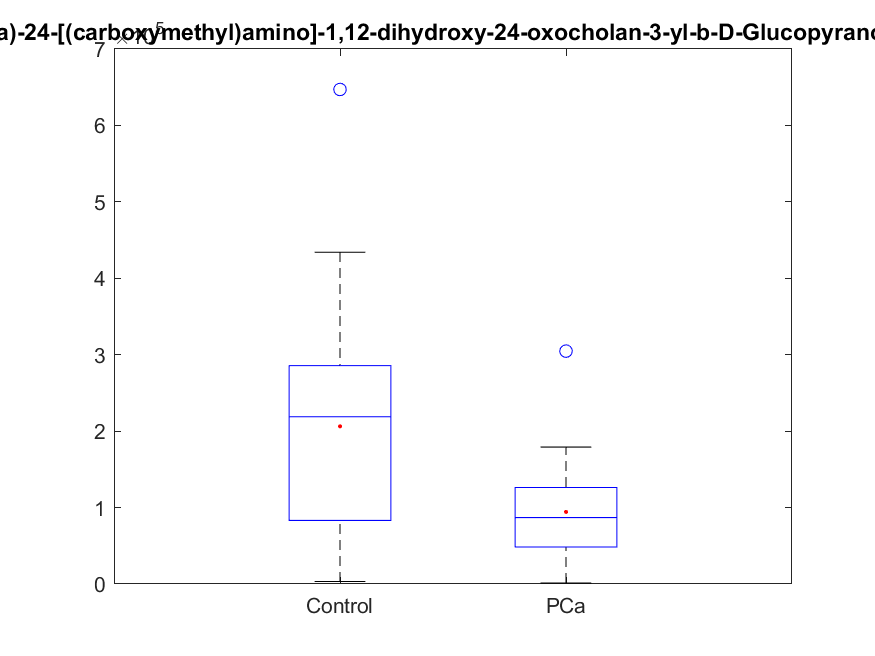


**Figure S71.** Box plot for compound 47 ESI- ((3a,5b,7a,12a)-24-[(carboxymethyl)amino]-1,12-dihydroxy-24-oxocholan-3-yl-b-D-Glucopyranosiduronic acid)
